# Validation of SNP markers for thermotolerance adaptation in *Ovis* *aries* adapted to different climatic regions using KASP-PCR technique

**DOI:** 10.1038/s41598-022-26909-1

**Published:** 2022-12-26

**Authors:** Putri Kusuma Astuti, Daniela Elena Ilie, Dinu Gavojdian, George Wanjala, Bouabid Badaoui, Husein Ohran, Eva Pasic-Juhas, Zoltán Bagi, András Jávor, Szilvia Kusza

**Affiliations:** 1 Centre of Agricultural Genomics and Biotechnology, University of Debrecen, Debrecen, 4032 Hungary; 2 Doctoral School of Animal Science, University of Debrecen, Debrecen, 4032 Hungary; 3 Research and Development Station for Bovine, 310059 Arad, Romania; 4 Research and Development Institute for Bovine Balotesti, 077015 Balotesti, Ilfov Romania; 5 Mohammed V University in Rabat, Morocco and African Sustainable Agriculture Research Institute (ASARI), Mohammed VI Polytechnic University (UM6P), Laâyoune, Morocco; 6 Department of Physiology, University of Sarajevo, Veterinary Faculty, 71 000 Sarajevo, Bosnia and Herzegovina

**Keywords:** Biodiversity, Conservation biology

## Abstract

A study on 51 SNPs belonging to 29 genes related to heat stress was carried out in 720 sheep from 17 different breeds adapted to different climates from Hungary, Bosnia and Herzegovina, Morocco and Romania, using Kompetitive Allele-Specific Polymerase Chain Reaction. Genotype frequency and the Hardy–Weinberg equilibrium were calculated, followed by a clustering using the Principal Component Analysis. We analyzed the polymorphisms in the following genes analyzed: *HSPA12A,* *HSP90AA1,* *IL33,* *DIO2,* *BTNL2,* *CSN2,* *ABCG1,* *CSN1S1,* *GHR,* *HSPA8,* *STAT3*, and *HCRT*. We emphasized on *HSPA12A* and HSPA8 genes as they were successfully genotyped in all studied flocks in which genotype frequency patterns were identified. Contrary to previous findings, the A allele for HSPA8 SNP was not observed in the heat tolerant breeds, being found exclusively in cold-tolerant breeds. The principal component analysis could not clearly differentiate the breeds, while plot concentration was slightly varied among the three groups, with *HSP90AA1* and *IL33* SNPs’ loading values significantly contributing to PC1 and PC2. We confirmed previous works that the *HSPA12A,* *HSPA8,* *HSP90AA1* and *IL33* SNPs are potential candidate markers for thermotolerance adaptation in sheep. This research contributes to the genetic variability of SNPs for thermotolerance adaptability in sheep.

## Introduction

Climate change is a complex phenomenon with disastrous consequences in almost all aspects of human societies, including in the livestock sector. Human activities would have contributed to a 1.0 °C increase in global temperature above pre-industrial levels. If current trends continue, global warming will likely approach 1.5 °C between 2030 and 2052^1^. Furthermore, according to reports from several international organizations involved in climate change research, practically all sectors of the economy and all regimes around the world will suffer from the negative effects of climate change, although to varying degrees, such as the irreversible loss of some natural ecosystems^2–5^. Particular attention should be paid to temperature variations as the most significant stressor on the efficiency of livestock production systems, affecting animal development, growth, production outputs and reproduction efficiency.

Compared to other ruminants, sheep tend to perform better in harsh environments, nevertheless, heat stress was shown to negatively impact physiology^6–8^, production parameters (milk production quality and quantity^9,10^, growth rates^11^), reproduction (male^12^ and female fertility^13^), sheep health^14^, and sheep welfare^15^, lowering farms economic returns and efficiency. Van Wettere et al.^16^ reported that when temperatures drop below 12 °C or increase over 31 °C, thermoregulatory mechanisms are severely disrupted and sheep's ability to maintain homeothermy is disturbed, with the impacts on performance and welfare being severe.

Heat stress is cytotoxic as it changes biological molecules, disrupts cell activities, affects metabolic responses, causes oxidative cell damage, and activates apoptosis and necrosis pathways^17–19^. In ewes, heat stress reduces estradiol concentration and aromatase activity, negatively influencing estrous occurrence and duration^20–22^. For each additional day of high-temperature exposure (> 32 °C) in a week prior to estrous onset, 2.7% of the fertilization rate and 3.5% of the lambing rate reduction were observed^16^. Heat stress also leads to higher circulating progesterone levels during the luteal phase, implying increased progesterone synthesis and decreased clearance, which impacts the timing and development of pre-ovulatory follicle growth^13^. Research with Malpura sheep housed in a climatic chamber for 6 h at 42 °C and 54% relative humidity found a significant drop in hemoglobin and packed cell volume levels as more water is transported through the thermoregulation circulatory system. Furthermore, higher plasma cortisol levels and increased cholesterol catabolism were involved in improving gluconeogenesis and providing more energy to heat-stressed rams. Because of the lower Gonadotropin releasing hormone (GnRH) release from the hypothalamus, plasma triiodothyronine (T3), thyroxine (T4) and testosterone levels were lowered, as were the sexual behaviour and semen volume, with less progressive sperm motility and sperm concentration^23,24^.

A critical component of adaptation to climate change is represented by the animals' genetic capacity to survive under stressful climatic conditions. Since it results in a permanent and cumulative transformation, the selection of thermoresistant animals could represent an efficient method of increasing livestock productivity during periods of high environmental temperature. Identifying and utilizing thermo-tolerant genotypes in sheep is critical due to the changing climate scenario, having the potential to significantly influence sheep productivity.

Thermotolerant genes in sheep have been studied by employing various genomic tools, for example, in heat stress protein genes; *HSP90* and *HSP70* genes polymorphism were investigated in Indian sheep using PCR–RFLP method^25^, *FGF2*, *GNAI13* and *PLCB1* melanogenesis candidate genes were studied in Egyptian Barki sheep breed, generated by 50K SNPs Beadchips^26^, and *HSP5* and *HSP40* in Brazilian sheep using the 50K SNP Chip^27^. Given that genetics has a complex role in affecting an individual’s capacity to respond to a stressful situation, more research into mechanisms and the development of more appropriate tools is required.

This study aimed to investigate the polymorphism of 51 SNPs in 29 genes involved in thermotolerance throughout the use of Kompetitive Allele Specific PCR (KASP-PCR) technique in 17 sheep breeds originating from different climatic conditions. The main goal was to identify and describe polymorphisms related to climatic adaptation that could be used to develop future thermal resilience in sheep through genomic selection.

## Results

### Allele and genotype frequency

A total of 601 animals (83.47%) from the total 720 animals were successfully genotyped and 32 SNPs (62.74%) among the initial set of 51 SNPs were successfully genotyped in this study; 17 of them were found to be polymorphic (33.33%) (see Table S1 of the Supplementary data), which were *HSPA12A,* *HSP90AA1,* *IL33,* *DIO2,* *BTNL2,* *CSN2,* *ABCG1,* *CSN1S1,* *GHR,* *HSPA8,* *STAT3*, and *HCRT*. Allelic and genotypic frequencies presented in Tables S2 and S3 of the Supplementary data, were different from one population to another. Four SNPs were successfully genotyped in all breeds: rs161504783-*HSP12A*, rs588145625-*HSPA8*, rs588498137-*STAT3* and rs602521720-*HCRT*.

The heterozygote *TC* for rs161504783-*HSPA12A* was dominant, except for Hungarian Racka, Transylvanian Merino, Hungarian Merino, Botosani Karakul and Sardi. The *T* and *C* allele frequencies were almost equally frequent in most breeds, except in Sardi (*T* allele = 0.107 and *C* alleles = 0.828) and Botosani Karakul (*T* allele = 0.735 and *C* alleles = 0.265).

The heterozygote *GA* for SNP rs588145625-*HSPA8* was absent in the heat tolerance breeds, except the Transylvanian Merino, and in breeds with high cold tolerance (Hungarian Racka, Babolna Tetra, Hungarian Tsigai, Romanian Racka, Pramenka and Turcana). The homozygote *GG* was present in all heat-tolerant breeds and some cold-tolerant breeds (Suffolk, Ile de France and Hungarian Merino). The *G* allele was dominant in all breeds, ranging from 0.760 to 1, and the *A* allele has only appeared in cold-tolerant breeds and Transylvanian Merino with frequency ranging from 0 to 0.308.

No patterns have been found for allelic and genotypic frequencies for rs588498137-*STAT3* and rs602521720-*HCRT* SNPs.

The *GG* genotype and *G* allele were dominant in all breeds for SNP rs588498137-*STAT3*, with *G* allelic frequency varying from 0.750 to 1. Similarly, the *CC* genotype and *C* allele for SNP rs602521720-*HCRT* was dominant in all breeds, with *C* allelic frequency varying from 0.546 to 1.

The Hardy–Weinberg equilibrium (HWE) was investigated with Chi-square (*x*^2^) test using allelic frequencies, observed and expected genotypes, and P value of the polymorphic genes are summarized in Table S2. Botosani Karakul breed from Romania was found to be the breed with most SNPs deviated from HWE (P ≤ 0.05); rs416259751-*IL33*, rs411181557-*DIO2*, rs414917134-*BTNL2* and rs420611298-*ABCG1*.

### Genetic diversity and interrelationship between SNPs

**Figure 1:**
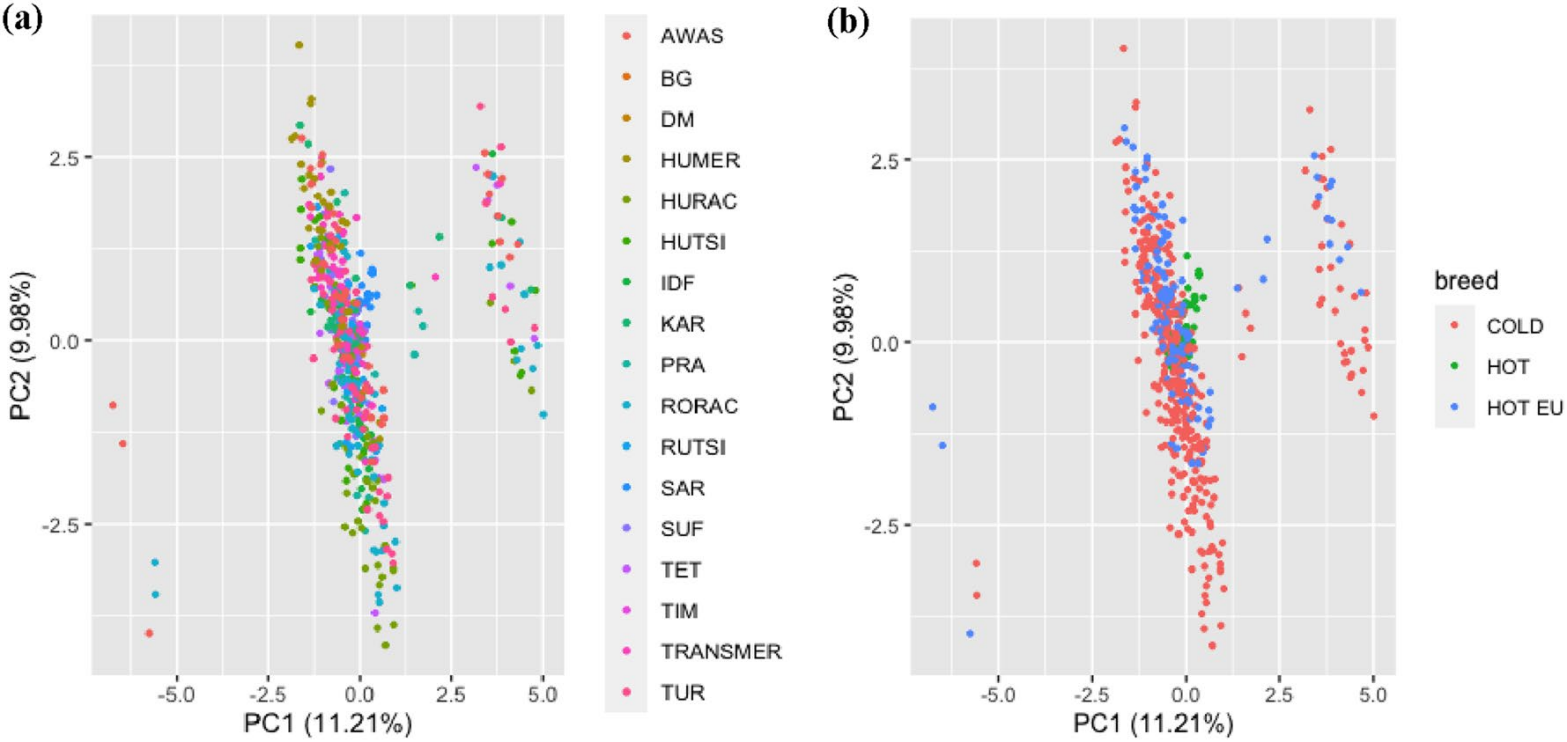
Score biplot of Principal Component Analysis of the 17 SNPs and 601 animals. (**a**) Individuals of different breeds are differently colored; (*AWAS* Hungarian Awassi, *BG* Béni Guil, *DM* D’Man, *HUMER* Hungarian Merino, *HURAC *Hungarian Racka, *HUTSI* Hungarian Tsigai, *IDF* Ile de France, *KAR* Botosani Karakul, *PRA* Pramenka, *RORAC* Romanian Racka, *ROTSI* Romanian Tsigai, *SAR* Sardi, *SUF* Suffolk, *TET* Babolna Tetra, *TIM* Timahdite, *TRANSMER* Transylvanian Merino and *TUR* Turcana). (**b**) Breeds grouped by climatic characteristics; COLD: cold-tolerant breeds (Suffolk, Babolna Tetra, Ile de France, Hungarian Tsigai, Hungarian Racka, Hungarian Merino, Pramenka, Romanian Racka and Turcana), HOT: heat tolerant breeds originated from Morocco (Béni Guil, D’Man, Timahdite and Sardi), HOT EU: Heat tolerant breeds reared in Europe (Hungarian Awassi, Botosani Karakul, Transylvanian Merino and Romanian Tsigai).

SNPs data were used to perform Principal Component Analysis (PCA) to highlight differences between either the breeds (Fig. 1a) or the climatic characteristics (Fig. 1b).

**Figure 2:**
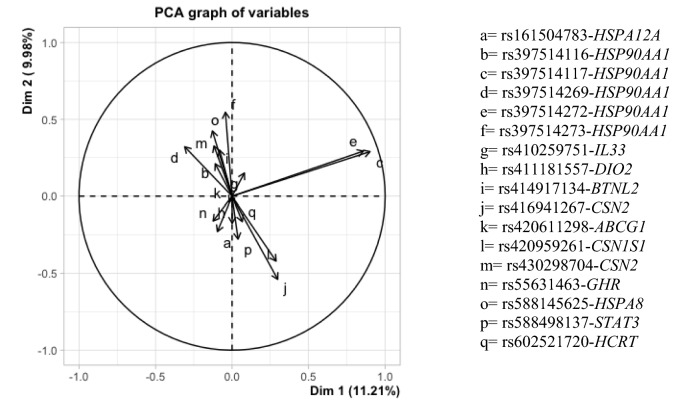
Loading biplot of principal component analysis of the 17 SNPs for 17 sheep breeds.

As displayed in Fig. 1, the PC1 and PC2 account for 11.21% and 9.98% of the total variation in the 17 breeds, respectively. Both PC1 and PC2 were unable to clearly separate neither the breeds (Fig. 1a), nor the climatic regions (Fig. 1b) even after we separated the heat tolerant breeds reared in the EU and in Morocco. All breeds were mostly concentrated in − 2.50 < PC1 < 1.12; cold tolerant breeds were outspread in PC1 score of − 5.593 to − 5.013 and PC2 score of − 4.149 to 4.020, heat-tolerant breeds were outspread in PC1 score of − 0.452 to 0.460 and PC2 score of − 0.157 to 1.183, while heat tolerant breeds reared in Europe had PC1 and PC2 ranging from − 0.881 to 0.686 and − 3.987 to 2.931, respectively. The highest contributions to the principal component were by rs397514117-*HSP90AA1* (c) and rs397514272-*HSP90AA1* (e), whereas rs410259751-*IL33* (g) also had a positive contribution (Fig. 2). The loading value and score value of PCA are available on Tables S4 and S5 of the Supplementary data.

## Discussion

The genetic background of thermotolerance adaptation in sheep represents a great interest for current research, especially when faced with the imminent increase of the earth's temperature because of climate change. Different breeds from different regions were used in this study, with the aim to see the different genetic backgrounds of thermotolerance adaptation according to geographical origins.

The application of KASP-PCR assay in this study did not show a high assay success rate as in previous studies carried out on goats^28^ and dairy cattle^29^. As much as 62.74% of SNPs were successfully genotyped in this study. These relatively high (37.26%) unsuccessful genotypes could be the consequence of sample damage during transportation from the laboratory to the subcontracting laboratory. Furthermore, quality control results in lower data for the analysis.

Only four SNPs were successfully genotyped in all investigated populations, and the focus was given to SNPs *HSPA12A* and *HSPA8* as they are the only ones that showed allelic and genotypic frequency patterns with the climatic characteristics. HSPs are a wide family of chaperone proteins categorized according to their molecular size and amino acid sequence similarity. The *HSPA12A* and *HSPA8* are members of the HSP70 family with a molecular weight of 70 kDa^30^. It is the biggest, most numerous and most conserved protein family throughout evolution, as well as the most extensively studied protein family across a wide range of species^31–33^. It is found on chromosome 15 and is made up of nine exons in sheep. This gene is widely studied for thermal adaptability. It controls cellular survival to heat stress with a highly dynamic nature, and these proteins are responsible for maintaining the organism's equilibrium and acclimating to heat stress^34,35^.

*HSPA12A* affects aspects like cellular senescence and how cells respond to heat stress^36^. It was found to be more active in ruminants during the summer, which helps them adapt to a hostile environment. This gene was found to be highly associated with heat tolerance in indicine cattle (*Bos* *taurus* *indicus*) and also has an important role in the water holding capacity in beef breeds, which might also act as a mechanism in surviving heat stress through the increased adrenaline and pH change in muscles^37,38^. According to the genotype and allele frequency results, the heterozygote *TC* for rs161504783-*HSPA12A* was dominant in most of the breeds, except Hungarian Racka, Hungarian Merino, Transylvanian Merino, Botosani Karakul and Sardi. Béni Guil, D’Man and Timahdite, breeds from a hot region, were not in HWE for HSPA12A.

In *HSP70*, exon 1 is non-coding, while the following eight exons combine to form the HSPA8 protein, with 650 amino acids of 71 kDa. HSPA8 aids in the day-to-day cell functions of protein folding and unfolding, polypeptide aggregation prevention, disassembly of large protein complexes and protein translocation across cellular compartments^35,39^. Given that increasing HSPA8 levels have been discovered to be positively correlated with heat tolerance, this gene has been employed as a candidate for heat resistance in many livestock species^40–42^.

In this study, the homozygote *GG* was carried by all heat-tolerant breeds and some cold-tolerant breeds (Suffolk, Ile de France and Romanian Tsigai), while the heterozygote *GA* for SNP rs588145625-*HSPA8* was only found in cold-tolerant breeds (Hungarian Racka, Babolna Tetra, Hungarian Tsigai, Romanian Racka, Transylvanian Merino, Pramenka and Turcana). The *A* allele was absent in heat tolerant breeds, which is opposed to the observation made by^25^ in a study of gene expression in Indian sheep, which discovered that animals with *A* allele have better hot climate adaptability, compared to animals with the *G* allele. The *AA* genotype is more adaptable to a hot environment and has lower *HSPA8* gene expression, than animals with the *AG* genotype. Similarly, a study by^41^ showed that the *GG* genotype has the least ability to survive heat stress in Awassi and Arabi sheep.

In this study, Botosani Karakul has shown to be the breed with the largest number of SNPs deviating from HWE. Lower heterozygosity was observed in 10 SNPs, and 4 of them deviated from HWE. One possible reason for this deviation of HWE was because Botosani Karakul is one of the Romanian crossbreeds that has undergone extensive genetic mixing from its German and Austria lines, and other Romanian breeds since its introduction from Russia at the beginning of the nineteenth century^43,44^.

PCA was unable to differentiate each breed in a clear and distinct manner; however, a relative clustering was observed based on the three different categories of climatic regions (Fig. 1b). This could be attributed to the fact that heat-tolerant breeds that are kept in the EU have become acclimated to the subtropical environment, in addition to the fact that genetic ad-mixture has occurred as a result of the widespread use of reproductive technologies, which has led to a less distinct genetic divergence between breeds, with previous studies reporting high levels of ad-mixture between different sheep breeds reared in Hungary and Romania, with the aim to increase the productivity^45–49^. We acknowledge that the cold breeds used in this study were not from year-around cold regions (e.g. Iceland, Finland, Norway), which was one of our limitations due to our inability to obtain samples from these regions. However, we strongly believe that the cold tolerance breeds from temperate climates utilized in this study are sufficiently contrasting with the Moroccan heat tolerant breeds.

From the loading biplot, *HSP90AA1* and *IL33* SNPs significantly contributed to PC1 and PC2. *HSP90AA1* has been confirmed to be associated with thermal stress susceptibility of sheep in previous studies^50,51^, while *IL33* was found to affect the sheep immunity and resistance to gastrointestinal intestinal nematode infection^52,53^, which is also associated because heat stress promotes to immune suppression and increases animal vulnerability to illnesses^54^. Our PCA findings validate these two SNPs as potential candidates for heat adaptability across the sheep breeds investigated in the current study, although more research is needed in order to clarify the relationship between the investigated SNPs and heat resistance.

## Conclusion

Based on our 17 SNP polymorphism analyses performed on 601 animals, we found that the KASP-PCR method represents a feasible method for investigating polymorphisms in different sheep breeds. Furthermore, based on allele and genotype frequency, we validated that *HSPA12A* and *HSPA8* SNPs are potential candidate markers for thermotolerance adaptation in sheep, whereas principal component analysis confirmed that *HSP90AA1* and *IL33* SNPs were the primary potential candidates. The results contribute to an increase in knowledge regarding the genetic variability of SNPs for thermotolerance adaptation in sheep. However, more studies are needed in order to clarify the relationship between the studied SNPs and heat resistance in sheep.

## Method

### Genomic DNA extraction

Samples were collected from 720 sheep belonging to17 breeds adapted to different climatic conditions, originating from 4 countries (Table 1). The majority of the breeds studied are indigenous to the country of origin, with some exotic breeds being included to determine if their acclimatization has contributed to adaptation. The breed characteristic (hot or cold tolerant) was determined based on the origin and development history of each breed in that particular country, as well as the environmental conditions under which the sample was collected (Table 2). All research activities were conducted in compliance with the European Union's Animal Experimentation Directive (Directive 2010/63/EU). FAO/IAEA^55^ recommended method from hair follicles and^56^ method from the blood was used for DNA genomic isolation. The DNA was kept at − 20 °C until analysis. The concentration of DNA was determined using a NanoDrop Spectrophotometer (Thermo Scientific, Waltham, MA, USA). All samples were diluted to a uniform concentration, and genotyping was performed using the equivalent of 50 ng of DNA per sample.

**Table 1:** Samples origin and breed characteristics.

Samples origin	Breed	Characteristic	Topographic origin	N	Sampling tissue
Hungary	Suffolk	Cold tolerant	Lowland	30	Blood
	Babolna Tetra	Cold tolerant	Lowland	36	Blood
	Ile de France	Cold tolerant	Lowland	33	Blood
	Hungarian Tsigai	Cold tolerant	Lowland	41	Blood
	Hungarian Racka	Cold tolerant	Lowland	48	Blood
	Hungarian Merino	Cold tolerant	Lowland	35	Blood
	Hungarian Awassi	Heat tolerant	Lowland	40	Blood
Bosnia and Herzegovina	Pramenka	Cold tolerant	Highland	37	Hair follicle
Morocco	Béni Guil	Heat tolerant	Lowland	30	Blood
	D'man	Heat tolerant	Lowland	30	Blood
	Timahdite	Heat tolerant	Highland	30	Blood
	Sardi	Heat tolerant	Highland	30	Blood
Romania	Botosani Karakul	Heat tolerant	Lowland	58	Hair follicle
	Romanian Racka	Cold tolerant	Highland	62	Hair follicle
	Transylvanian Merino	Heat tolerant	Lowland	60	Hair follicle
	Romanian Tsigai	Heat tolerant	Lowland	60	Hair follicle
	Turcana	Cold tolerant	Highland	60	Hair follicle

**Table 2:** Sampling region climatological details.

Sampling region	Breeds	Altitude (m)	Temp. (°C)	
			Min	Max
Szendrő, Hungary	Suffolk Babolna Tetra Ile de France	147	− 7.6	24.9
Hortobágy, Hungary	Hungarian Tsigai Hungarian Racka	85	− 7.9	25.3
Karcag, Hungary	Hungarian Merino	79	− 6.5	25.8
Bakonszeg, Hungary	Hungarian Awassi	82	− 7.9	25.3
Botoșani, Romania	Botosani Karakul	198	− 6.0	27.0
Caraș-Severin, Romania	Romanian Racka Turcana	1251	− 6.0	19.0
Baia Mare, Romania	Transylvanian Merino	256	− 5.0	26.0
Arad, Romania	Romanian Tsigai	90	− 2.0	28.0
Timis, Romania	Turcana	116	− 4.0	28.0
Dub, Mount Vlašić, Bosnia and Herzegovina	Pramenka	654	− 11.0	22.0
Eastern region of Morocco	Béni Guil	542–1706	2.0	38.0
Central plateau of Morocco	Sardi	369–793	− 2.0	37.0
Oases of the South of Morocco	D’man	1026–1133	3.0	39.0
Middle Atlas of Morocco	Timahdite	1818	− 2.0	34.0

### Selection of SNPs

Based on the findings of previous genome-wide association studies (GWAS) and marker-assisted selection studies across the sheep genome^25,41,57–59^, a SNP panel of 51 SNPs from 29 genes related to heat stress was selected, which included loci distributed on 18 chromosomes (Table 3). The Ovis SNP data were obtained from the Single Nucleotide Polymorphism Database (dbSNP) from the National Center for Biotechnology Information (NCBI) or Ensembl a few years ago, some of the RS reference IDs might have changed up to the present time. Due to the frequently changing genotyping database and varying reference sequences from those available at the European Variation Archive (EVA), only 10 SNPs out of 17 SNPs are deposited on EVA.

**Table 3:** Selected SNPs used for studying thermotolerance adaptation genes in various sheep breeds.

	SNP	Locus	Gene name	Allele substitution	Chromosome
1	rs593507294	*LEP*	Leptin	C/T	4
2	rs161110765	*SOCS3*	Suppressor of cytokine signalling 3	A/C	11
3	rs161286575	*PPARG*	Peroxisome proliferator-activated receptor gamma	C/T	19
4	rs603870279	*ASIP*	Agouti signalling protein	C/T	13
5	rs598380853	*ASIP*	Agouti signalling protein	C/G	13
6	rs601650611	*ASIP*	Agouti signalling protein	C/G	13
7	rs420959261	*CSN1S1*	Casein alpha s1	C/T	6
8	rs587905107	*CSN1S1*	Casein alpha s1	C/T	6
9	rs416941267	*CSN2*	Casein beta	G/T	6
10	rs430298704	*CSN2*	Casein beta	C/T	6
11	rs420611298	*ABCG1*	ATP binding cassette subfamily G member 1	G/T	1
12	rs159956881	*ABCG2*	ATP binding cassette subfamily G member 1	A/G	6
13	rs159876394	*IGF1*	Insulin like growth factor 1	C/G	3
14	rs160257833	*ESR1*	Oestrogen receptor 1	A/G	8
15	rs591182158	*ESR1*	Oestrogen receptor 1	A/G	8
16	rs598908205	*GNRH1*	Gonadotropin releasing hormone 1	C/T	2
17	rs411181557	*DIO2*	Deiodinase iodothyronine type II	C/G	7
18	rs414917134	*BTNL2*	Butyrophilin like 2	C/G	20
19	rs405270595	*BTN1A1*	Butyrophilin	A/G	20
20	rs161146164	*GHR*	Growth hormone receptor	G/T	16
21	rs55631463	*GHR*	Growth hormone receptor	A/G	16
22	rs407318935	*STAT1*	Signal transducer and activator of transcription 1	A/G	2
23	rs161691559	*HSP90AB1*	Heat shock protein 90 alpha family class B member 1	A/G	20
24	rs397514115	*HSP90AA1*	Heat shock protein 90 alpha family class A member 1	G/C	18
25	rs397514116	*HSP90AA1*	Heat shock protein 90 alpha family class A member 1	C/G	18
26	rs397514117	*HSP90AA1*	Heat shock protein 90 alpha family class A member 1	A/C	18
27	rs397514269	*HSP90AA1*	Heat shock protein 90 alpha family class A member 1	A/G	18
28	rs397514270	*HSP90AA1*	Heat shock protein 90 alpha family class A member 1	G/T	18
29	rs397514271	*HSP90AA1*	Heat shock protein 90 alpha family class A member 1	A/G	18
30	rs397514268	*HSP90AA1*	Heat shock protein 90 alpha family class A member 1	-/G	18
31	rs397514272	*HSP90AA1*	Heat shock protein 90 alpha family class A member 1	G/T	18
32	rs397514273	*HSP90AA1*	Heat shock protein 90 alpha family class A member 1	A/G	18
33	rs588145625	*HSPA8*	Heat shock protein family A member 8	A/G	15
34	rs161504783	*HSPA12A*	Heat shock protein family A member 12A	C/T	22
35	rs160077209	*HSPA4*	Heat shock protein family A member 4	A/G	5
36	rs589164764	*IL1R1*	Interleukin 1 receptor type 1	C/T	3
37	rs160387232	*IL1R1*	Interleukin 1 receptor type 1	C/T	3
38	rs590620426	*IL2*	Interleukin 2	C/G	17
39	rs596312311	*IL2*	Interleukin 2	C/T	17
40	rs416425182	*TR*	Thyroglobulin	A/C	9
41	rs595200178	*TR*	Thyroglobulin	A/G	9
42	rs418400798	*TR*	Thyroglobulin	C/T	9
43	rs410259751	*IL33*	Interleukin 33	G/T	2
44	rs162295351	*HSP90AB1*	Heat shock protein 90 alpha family class B member 1	A/C	20
45	rs161691552	*HSP90AB1*	Heat shock protein 90 alpha family class B member 1	A/G	20
46	rs597293577	*STAT_PIAS3*	Protein inhibitor of activated STAT, 3	C/T	1
47	rs593155540	*STAT_PIAS3*	Protein inhibitor of activated STAT, 3	A/G	1
48	rs602521720	*HCRT*	Hypocretin neuropeptide precursor	C/G	11
49	rs425706327	*USP19*	Ubiquitin specific peptidase 19	A/G	19
50	rs161274296	*USP19*	Ubiquitin specific peptidase 19	G/T	19
51	rs588498137	*STAT3*	Signal transducer and activator of transcription 3	A/G	11

### Genotyping and quality control

The bi-allelic discrimination of the selected 51 SNPs was performed using Kompetitive Allele Specific PCR (KASPTM, LGC Genomics, Teddington, Middlesex, UK). SNP Viewer software version 1.99 (Hoddesdon, UK) was used to visualize the results. All genotype data were exported for statistical analysis. Only SNPs that appeared in at least 50% of the breeds were considered. Data quality control of genotyped data included discarding animals with a call rate of less than 50% and the SNPs with call rates < 50%. This led to discrepancies in either the number of animals per breed or the number of SNPs per animal.

### Data analysis

The raw allele calls obtained from LGC Genomics were analyzed using LGC Genomics' KlusterCaller program. Gene diversity, allele and genotype frequencies, and their accordance with or deviation from the Hardy–Weinberg equilibrium were determined by POPGENE software version 1.31^60^

The Principal Component Analysis was done using FactoMineR^61^ and ggplot2^62^ packages from the R Program^63^ to visualize the genetic divergences between sheep breeds that were divided according to their climatic characteristics; cold-tolerant breeds (Babolna Tetra, Hungarian Merino, Hungarian Racka, Hungarian Tsigai, Ile de France, Pramenka, Romanian Racka, Suffolk and Turcana), heat tolerance breeds originated from Morocco (Béni Guil, D’Man, Timahdite and Sardi), and heat tolerant breeds reared in Europe (Hungarian Awassi, Botosani Karakul, Transylvanian Merino, and Romanian Tsigai).

### Ethical approval

The authors confirm that the experiment complied with the European Union's Directive on Animal Experimentation (Directive 2010/63/EU) and ARRIVE guidelines. All animals in the experiment underwent standard procedures without experiencing any harm or discomfort, and all procedures were carried out in compliance with applicable guidelines and regulations. The study was approved by the Scientific and Ethics Committee of Centre for Agricultural Genomics and Biotechnology, University of Debrecen (Ethics statement No. 07).

## Supplementary Information

Supplementary Information.

## Data Availability

The 10 SNPs genotype datasets generated and analyzed in this study are only partially available at the EVA under accession number ERZ6760182. The remaining 7 SNPs genotype data is available in the supplementary material file (Table S6) (https://www.ebi.ac.uk/ena/browser/view/ERZ7485042?show=analyses).
