# Supplementary material for: Validation of SNP markers for thermotolerance adaptation in Ovis aries adapted to different climatic regions using KASP-PCR technique
Source: Sci Rep. 2022 Dec 26;12:22348. doi: 10.1038/s41598-022-26909-1 (PMC9792578; doi:10.1038/s41598-022-26909-1)
Supplement: Supplementary file 1 — Supplementary Information. [file 41598_2022_26909_MOESM1_ESM.docx]

**Supplementary Documents**

Table S1. The genotyped SNPs

|  | SNP | Gene | Allele substitution | Chromosome | Genotype |
| --- | --- | --- | --- | --- | --- |
| 1 | [rs159876394*](https://www.ncbi.nlm.nih.gov/SNP/snp_ref.cgi?rs=159876394) | IGF1 | C/G | 3 | CC |
|  |  |  |  |  | GC |
|  |  |  |  |  | GG |
| 2 | rs159956881* | ABCG2 | A/G | 6 | GG |
|  |  |  |  |  | TG |
|  |  |  |  |  | TT |
| 3 | [rs160257833](https://www.ncbi.nlm.nih.gov/SNP/snp_ref.cgi?rs=160257833)* | ESR1 | A/G | 8 | AA |
|  |  |  |  |  | GA |
|  |  |  |  |  | GG |
| 4 | [rs161274296](https://www.ncbi.nlm.nih.gov/SNP/snp_ref.cgi?rs=161274296)* | USP19 | G/T | 19 | GG |
|  |  |  |  |  | GC |
|  |  |  |  |  | CC |
| 5 | [rs161504783](https://www.ncbi.nlm.nih.gov/SNP/snp_ref.cgi?rs=161504783) | HSPA12A | C/T | 22 | CC |
|  |  |  |  |  | TC |
|  |  |  |  |  | TT |
| 6 | rs161691552 * | HSP90AB1 | G/A | 20 | GG |
|  |  |  |  |  | GC |
|  |  |  |  |  | CC |
| 7 | [rs161691559](https://www.ncbi.nlm.nih.gov/SNP/snp_ref.cgi?rs=161691559)* | HSP90AB1 | G/A | 20 | GG |
|  |  |  |  |  | GC |
|  |  |  |  |  | CC |
| 8 | [rs162295351](https://www.ncbi.nlm.nih.gov/projects/SNP/snp_ref.cgi?rs=162295351)* | HSP90AB1 | A/C | 20 | CC |
|  |  |  |  |  | GC |
|  |  |  |  |  | GG |
| 9 | [rs397514115](https://www.ncbi.nlm.nih.gov/projects/SNP/snp_ref.cgi?rs=397514115)* | HSP90AA1 | G/C | 18 | GG |
|  |  |  |  |  | GC |
|  |  |  |  |  | CC |
| 10 | rs397514116 | HSP90AA1 | C/G | 18 | GG |
|  |  |  |  |  | GC |
|  |  |  |  |  | CC |
| 11 | rs397514117 | HSP90AA1 | A/C | 18 | AA |
|  |  |  |  |  | CA |
|  |  |  |  |  | CC |
| 12 | rs397514269 | HSP90AA1 | A/G | 18 | GG |
|  |  |  |  |  | GA |
|  |  |  |  |  | AA |
| 13 | rs397514272 | HSP90AA1 | G/T | 18 | TT |
|  |  |  |  |  | TG |
|  |  |  |  |  | GG |
| 14 | rs397514273 | HSP90AA1 | A/G | 18 | GG |
|  |  |  |  |  | GA |
|  |  |  |  |  | AA |
| 15 | rs410259751 | IL_33 | G/T | 2 | GG |
|  |  |  |  |  | TG |
|  |  |  |  |  | TT |
| 16 | rs411181557 | DIO2 | C/G | 7 | CC |
|  |  |  |  |  | GC |
|  |  |  |  |  | GG |
| 17 | [rs414917134](https://www.ncbi.nlm.nih.gov/SNP/snp_ref.cgi?rs=414917134) | BTNL2 | C/G | 20 | GG |
|  |  |  |  |  | GC |
|  |  |  |  |  | CC |
| 18 | [rs416941267](https://www.ncbi.nlm.nih.gov/SNP/snp_ref.cgi?rs=416941267) | CSN2 | G/T | 6 | TT |
|  |  |  |  |  | TG |
|  |  |  |  |  | GG |
| 19 | [rs420611298](https://www.ncbi.nlm.nih.gov/SNP/snp_ref.cgi?rs=420611298) | ABCG1 | G/T | 1 | TT |
|  |  |  |  |  | TG |
|  |  |  |  |  | GG |
| 20 | rs420959261 | CSN1S1 | C/T | 6 | TT |
|  |  |  |  |  | TC |
|  |  |  |  |  | CC |
| 21 | rs430298704 | CSN2 | C/T | 6 | TT |
|  |  |  |  |  | TC |
|  |  |  |  |  | CC |
| 22 | [rs55631463](https://www.ncbi.nlm.nih.gov/SNP/snp_ref.cgi?rs=55631463) | GHR | A/G | 16 | GG |
|  |  |  |  |  | GA |
|  |  |  |  |  | AA |
| 23 | rs587905107* | CSN1S1 | C/T | 6 | TT |
|  |  |  |  |  | TG |
|  |  |  |  |  | GG |
| 24 | [rs588145625](https://www.ncbi.nlm.nih.gov/SNP/snp_ref.cgi?rs=588145625) | HSPA8 | A/G | 15 | GG |
|  |  |  |  |  | GA |
|  |  |  |  |  | AA |
| 25 | rs588498137 | STAT3 | A/G | 11 | GG |
|  |  |  |  |  | GA |
|  |  |  |  |  | AA |
| 26 | [rs589164764](https://www.ncbi.nlm.nih.gov/SNP/snp_ref.cgi?rs=589164764)* | IL1R1 | C/T | 3 | CC |
|  |  |  |  |  | TC |
|  |  |  |  |  | TT |
| 27 | [rs590620426](https://www.ncbi.nlm.nih.gov/SNP/snp_ref.cgi?rs=590620426)* | IL2 | C/G | 17 | CC |
|  |  |  |  |  | GC |
|  |  |  |  |  | GG |
| 28 | [rs591182158](https://www.ncbi.nlm.nih.gov/SNP/snp_ref.cgi?rs=591182158)* | ESR1 | A/G | 8 | GG |
|  |  |  |  |  | GC |
|  |  |  |  |  | CC |
| 29 | [rs596312311](https://www.ncbi.nlm.nih.gov/SNP/snp_ref.cgi?rs=596312311)* | IL2 | C/T | 17 | GG |
|  |  |  |  |  | GC |
|  |  |  |  |  | CC |
| 30 | [rs597293577](https://www.ncbi.nlm.nih.gov/SNP/snp_ref.cgi?rs=597293577)* | STAT_PIAS3 | C/T | 1 | TT |
|  |  |  |  |  | TC |
|  |  |  |  |  | CC |
| 31 | [rs602521720](https://www.ncbi.nlm.nih.gov/SNP/snp_ref.cgi?rs=602521720) | HCRT | C/G | 19 | CC |
|  |  |  |  |  | GC |
|  |  |  |  |  | GG |
| 32 | rs603870279 * | ASIP | C/T | 11 | CC |
|  |  |  |  |  | CT |
|  |  |  |  |  | TT |

* Monomorphic loci

Table S2. Polymorphic SNP and genotype frequencies in the 15 sheep breeds

|  |  |  | **POP 1: PRAMENKA** | | | | | | | **POP 2: BENI GUIL** | | | | | | | | **POP 3: D'MAN** | | | | | | |
| --- | --- | --- | --- | --- | --- | --- | --- | --- | --- | --- | --- | --- | --- | --- | --- | --- | --- | --- | --- | --- | --- | --- | --- | --- |
|  | **GENO**  **TYPE** | **ALLELE** | **Genotype Freq** | **Allele Freq** | **Fis** | **𝑥²** | **H obs** | **H exp** | **n** | **Genotype Freq** | **Allele Freq** | **Fis** | **𝑥²** | | **H obs** | **H exp** | **n** | **Genotype Freq** | **Allele Freq** | **Fis** | **𝑥²** | **H obs** | **H exp** | **n** |
| **SNP1** | CC | C | 8 | 0,531 | -0,129 | 0,413 | 0,563 | 0,502 | 64 | 1 | 0,500 | -0,833 | 15,834* | | 0,927 | 0,511 | 48 | 0 | 0,432 | -0,760 | 11,970* | 0,864 | 0,502 | 44 |
|  | TC | T | 18 | 0,469 | -0,129 |  |  |  |  | 22 | 0,500 | -0,833 |  |  |  |  |  | 19 | 0,568 | -0,760 |  |  |  |  |
|  | TT |  | 6 |  |  |  |  |  |  | 1 |  |  |  |  |  |  |  | 3 |  |  |  |  |  |  |
| **SNP2** | CC | C | 21 | 0,818 | -0,220 | 1,476 | 0,354 | 0,302 | 66 | NA | NA | NA | NA | | NA | NA | NA | NA | NA | NA | NA | NA | NA | NA |
|  | GC | G | 12 | 0,000 | -0,220 |  |  |  |  | NA | NA | NA |  |  |  |  |  | NA | NA | NA |  |  |  |  |
|  | GG |  | 0 |  |  |  |  |  |  | NA |  |  |  |  |  |  |  | NA |  |  |  |  |  |  |
| **SNP3** | AA | A | 0 | 0,000 | 0,000 | X | 0 | 0 | 54 | NA | NA | NA | NA | | NA | NA | NA | NA | NA | NA | NA | NA | NA | NA |
|  | CA | C | 0 | 0,000 | 0,000 |  |  |  |  | NA | NA | NA |  |  |  |  |  | NA | NA | NA |  |  |  |  |
|  | CC | C | 54 | 1,000 |  |  |  |  |  | NA |  |  |  |  |  |  |  | NA |  |  |  |  |  |  |
| **SNP4** | AA | A | NA | NA | NA | NA | NA | NA | NA | NA | NA | NA | NA | | NA | NA | NA | NA | NA | NA | NA | NA | NA | NA |
|  | GA | G | NA | NA | NA |  |  |  |  | NA | NA | NA |  |  |  |  |  | NA | NA | NA |  |  |  |  |
|  | GG |  | NA |  |  |  |  |  |  | NA |  |  |  |  |  |  |  | NA |  |  |  |  |  |  |
| **SNP5** | GG | G | 0 | 0,050 | -0,526 | 0,055 | 0,1 | 0,097 | 60 | NA | NA | NA | NA | | NA | NA | NA | NA | NA | NA | NA | NA | NA | NA |
|  | TG | T | 3 | 0,950 | -0,526 |  |  |  |  | NA | NA | NA |  |  |  |  |  | NA | NA | NA |  |  |  |  |
|  | TT |  | 27 |  |  |  |  |  |  | NA |  |  |  |  |  |  |  | NA |  |  |  |  |  |  |
| **SNP6** | AA | A | 0 | 0,224 | -0,289 | 2,206 | 0,448 | 0,381 | 58 | NA | NA | NA | NA | | NA | NA | NA | NA | NA | NA | NA | NA | NA | NA |
|  | GA | G | 13 | 0,776 | -0,289 |  |  |  |  | NA | NA | NA |  |  |  |  |  | NA | NA | NA |  |  |  |  |
|  | GG |  | 16 |  |  |  |  |  |  | NA |  |  |  |  |  |  |  | NA |  |  |  |  |  |  |
| **SNP7** | GG | G | 2 | 0,250 | 0,022 | 0,047 | 0,367 | 0,421 | 60 | 2 | 0,538 | -0,587 | 18,254* | | 0,923 | 0,507 | 52 | NA | NA | NA | NA | NA | NA | NA |
|  | TG | T | 11 | 0,750 | 0,022 |  |  |  |  | 24 | 0,462 | -0,587 |  |  |  |  |  | NA | NA | NA |  |  |  |  |
|  | TT |  | 17 |  |  |  |  |  |  | 0 |  |  |  |  |  |  |  | NA |  |  |  |  |  |  |
| **SNP8** | CC | C | 4 | 0,294 | 0,150 | 0,937 | 0,253 | 0,421 | 68 | NA | NA | NA | NA | | NA | NA | NA | NA | NA | NA | NA | NA | NA | NA |
|  | CG | G | 12 | 0,706 | 0,150 |  |  |  |  | NA | NA | NA |  |  |  |  |  | NA | NA | NA |  |  |  |  |
|  | GG |  | 18 |  |  |  |  |  |  | NA |  |  |  |  |  |  |  | NA |  |  |  |  |  |  |
| **SNP9** | CC | C | NA | NA | NA | NA | NA | NA | NA | NA | NA | NA | NA | | NA | NA | NA | NA | NA | NA | NA | NA | NA | NA |
|  | GC | G | NA | NA | NA |  |  |  |  | NA | NA | NA |  |  |  |  |  | NA | NA | NA |  |  |  |  |
|  | GG |  | NA |  |  |  |  |  |  | NA |  |  |  |  |  |  |  | NA |  |  |  |  |  |  |
| **SNP10** | GG | G | 15 | 0,662 | 0,015 | 0,030 | 0,441 | 0,454 | 68 | NA | NA | NA | NA | | NA | NA | NA | NA | NA | NA | NA | NA | NA | NA |
|  | TG | T | 15 | 0,338 | 0,015 |  |  |  |  | NA | NA | NA |  |  |  |  |  | NA | NA | NA |  |  |  |  |
|  | TT |  | 4 |  |  |  |  |  |  | NA |  |  |  |  |  |  |  | NA |  |  |  |  |  |  |
| **SNP11** | GG | G | NA | NA | NA | NA | NA | NA | NA | NA | NA | NA | NA | | NA | NA | NA | NA | NA | NA | NA | NA | NA | NA |
|  | TG | T | NA | NA | NA |  |  |  |  | NA | NA | NA |  |  |  |  |  | NA | NA | NA |  |  |  |  |
|  | TT |  | NA |  |  |  |  |  |  | NA |  |  |  |  |  |  |  | NA |  |  |  |  |  |  |
| **SNP12** | CC | C | NA | NA | NA | NA | NA | NA | NA | NA | NA | NA | NA | | NA | NA | NA | NA | NA | NA | NA | NA | NA | NA |
|  | TC | T | NA | NA | NA |  |  |  |  | NA | NA | NA |  |  |  |  |  | NA | NA | NA |  |  |  |  |
|  | TT |  | NA |  |  |  |  |  |  | NA |  |  |  |  |  |  |  | NA |  |  |  |  |  |  |
| **SNP13** | CC | C | 0 | 0,058 | -0,061 | 0,064 | 0,115 | 0,111 | 52 | NA | NA | NA | NA | | NA | NA | NA | NA | NA | NA | NA | NA | NA | NA |
|  | TC | T | 3 | 0,942 | -0,061 |  |  |  |  | NA | NA | NA |  |  |  |  |  | NA | NA | NA |  |  |  |  |
|  | TT |  | 23 |  |  |  |  |  |  | NA |  |  |  |  |  |  |  | NA |  |  |  |  |  |  |
| **SNP14** | AA | A | 0 | 0,000 | 0,000 | X | 0 | 0 | 44 | NA | NA | NA | NA | | NA | NA | NA | NA | NA | NA | NA | NA | NA | NA |
|  | GA | G | 0 | 1,000 | 0,000 |  |  |  |  | NA | NA | NA |  |  |  |  |  | NA | NA | NA |  |  |  |  |
|  | GG |  | 22 |  |  |  |  |  |  | NA |  |  |  |  |  |  |  | NA |  |  |  |  |  |  |
| **SNP15** | AA | A | 0 | 0,106 | -0,119 | 0,393 | 0,212 | 0,193 | 66 | 0 | 0,000 | 0,000 | X | | 1,000 | 1,000 | 48 | 0 | 0,000 | 0 | X | 0 | 0 | 36 |
|  | GA | G | 7 | 0,894 | -0,119 |  |  |  |  | 0 | 1,000 | 0,000 |  |  |  |  |  | 0 | 1 | 0 |  |  |  |  |
|  | GG |  | 26 |  |  |  |  |  |  | 24 |  |  |  |  |  |  |  | 18 |  |  |  |  |  |  |
| **SNP16** | AA | A | 0 | 0,014 | -0,014 | 0,000 | 0,378 | 0,028 | 72 | 0 | 0,000 | 0,000 | X | | 1,000 | 1,000 | 58 | 0 | 0,000 | 0 | X | 0 | 0 | 58 |
|  | GA | G | 1 | 0,986 | -0,014 |  |  |  |  | 0 | 1,000 | 0,000 |  |  |  |  |  | 0 | 1 | 0 |  |  |  |  |
|  | GG |  | 35 |  |  |  |  |  |  | 29 |  |  |  |  |  |  |  | 29 |  |  |  |  |  |  |
| **SNP17** | CC | C | 32 | 0,985 | -0,154 | 0,000 | 0,030 | 0,030 | 66 | 27 | 1,000 | 0,000 | X | | 1,000 | 1,000 | 54 | 25 | 0,981 | -0,020 | 0 | 0,039 | 0,039 | 52 |
|  | GC | G | 1 | 0,015 | -0,154 |  |  |  |  | 0 | 0,000 | 0,000 |  |  |  |  |  | 1 | 0,019 | -0,020 |  |  |  |  |
|  | GG |  | 0 |  |  |  |  |  |  | 0 |  |  |  |  |  |  |  | 0 |  |  |  |  |  |  |
|  |  |  |  |  |  |  |  |  |  |  |  |  |  | |  |  |  |  |  |  |  |  |  |  |
|  |  |  | **POP 4: TIMAHDITE** | | | | | | | **POP 5: SARDI** | | | | | | | | **POP 6: SUFFOLK** | | | | | | |
|  | GENOTYPE | ALLELE | Geno  type Freq | Allele Freq | Fis | 𝑥² | H obs | H exp | n | Genotype Freq | Allele Freq | Fis | 𝑥² | | H obs | H exp | n | Geno  type Freq | Allele Freq | Fis | 𝑥² | H obs | H exp | n |
| **SNP1** | CC | C | 3 | 0,553 | -0,597 | 6,177* | 0,789 | 0,501 | 38 | 11 | 0,893 | -0,120 | 0,130 | | 0,214 | 0,198 | 28 | 9 | 0,577 | 0,055 | 0,143 | 0,462 | 0,498 | 52 |
|  | TC | T | 15 | 0,448 | -0,597 |  |  |  |  | 3 | 0,107 | -0,120 |  |  |  |  |  | 12 | 0,423 | 0,055 |  |  |  |  |
|  | TT |  | 1 |  |  |  |  |  |  | 0 |  |  |  |  |  |  |  | 5 |  |  |  |  |  |  |
| **SNP2** | CC | C | NA | NA | NA | NA | NA | NA | NA | NA | NA | NA | NA | | NA | NA | NA | 6 | 0,539 | -0,238 | 1,242 | 0,615 | 0,507 | 52 |
|  | GC | G | NA | NA | NA |  |  |  |  | NA | NA | NA |  |  |  |  |  | 26 | 0,461 | -0,238 |  |  |  |  |
|  | GG |  | NA |  |  |  |  |  |  | NA |  |  |  |  |  |  |  | 4 |  |  |  |  |  |  |
| **SNP3** | AA | A | 0 | 1 | 0 | x | 0 | 0 | 44 | 0 | 0,500 | -1,000 | 21,00* | | 1,000 | 0,512 | 44 | 0 | 0,981 | -0,020 | 0,000 | 0,039 | 0,039 | 52 |
|  | CA | C | 0 | 0 | 0 |  |  |  |  | 22 | 0,500 | -1,000 |  |  |  |  |  | 1 | 0,019 | -0,020 |  |  |  |  |
|  | CC | C | 22 |  |  |  |  |  |  | 0 |  |  |  |  |  |  |  | 25 |  |  |  |  |  |  |
| **SNP4** | AA | A | NA | NA | NA | NA | NA | NA | NA | NA | NA | NA | NA | | NA | NA | NA | 6 | 0,019 | -0,325 | 2,433 | 0,654 | 0,503 | 52 |
|  | GA | G | NA | NA | NA |  |  |  |  | NA | NA | NA |  |  |  |  |  | 17 | 0,981 | -0,325 |  |  |  |  |
|  | GG |  | NA |  |  |  |  |  |  | NA |  |  |  |  |  |  |  | 3 |  |  |  |  |  |  |
| **SNP5** | GG | G | 0 | 0 | 0 | x | 0 | 0 | 48 | NA | NA | NA | NA | | NA | NA | NA | 0 | 0,192 | -0,020 | 0,000 | 0,039 | 0,039 | 52 |
|  | TG | T | 0 | 1 | 0 |  |  |  |  | NA | NA | NA |  |  |  |  |  | 1 | 0,981 | -0,020 |  |  |  |  |
|  | TT |  | 24 |  |  |  |  |  |  | NA |  |  |  |  |  |  |  | 25 |  |  |  |  |  |  |
| **SNP6** | AA | A | NA | NA | NA | NA | NA | NA | NA | 0 | 0,000 | 0,000 | x | | 0,000 | 1,000 | 54 | 0 | 0,039 | -0,040 | 0,020 | 0,769 | 0,075 | 52 |
|  | GA | G | NA | NA | NA |  |  |  |  | 0 | 1,000 |  |  |  |  |  |  | 2 | 0,962 | -0,040 |  |  |  |  |
|  | GG |  | NA |  |  |  |  |  |  | 27 |  |  |  |  |  |  |  | 24 |  |  |  |  |  |  |
| **SNP7** | GG | G | 0 | 0,479 | -0,92 | 19,397* | 0,958 | 0,510 | 48 | 0 | 0,480 | -0,923 | 20,382* | | 0,960 | 0,509 | 50 | 8 | 0,460 | 0,436 | 5,227* | 0,280 | 0,503 | 50 |
|  | TG | T | 23 | 0,521 | -0,92 |  |  |  |  | 24 | 0,520 | -0,923 |  |  |  |  |  | 7 | 0,540 | 0,436 |  |  |  |  |
|  | TT |  | 1 |  |  |  |  |  |  | 1 |  |  |  |  |  |  |  | 10 |  |  |  |  |  |  |
| **SNP8** | CC | C | NA | NA | NA | NA | NA | NA | NA | NA | NA | NA | NA | | NA | NA | NA | 3 | 0,327 | 0,039 | 0,089 | 0,423 | 0,039 | 52 |
|  | CG | G | NA | NA | NA |  |  |  |  | NA | NA | NA |  |  |  |  |  | 11 | 0,673 | 0,039 |  |  |  |  |
|  | GG |  | NA |  |  |  |  |  |  | NA |  |  |  |  |  |  |  | 12 |  |  |  |  |  |  |
| **SNP9** | CC | C | NA | NA | NA | NA | NA | NA | NA | NA | NA | NA | NA | | NA | NA | NA | 26 | 1,000 | 0,000 | 0,000 | 0,000 | 0,000 | 52 |
|  | GC | G | NA | NA | NA |  |  |  |  | NA | NA | NA |  |  |  |  |  | 0 | 0,000 |  |  |  |  |  |
|  | GG |  | NA |  |  |  |  |  |  | NA |  |  |  |  |  |  |  | 0 |  |  |  |  |  |  |
| **SNP10** | GG | G | NA | NA | NA | NA | NA | NA | NA | NA | NA | NA | NA | | NA | NA | NA | 14 | 0,750 | -0,128 | 0,315 | 0,423 | 0,382 | 52 |
|  | TG | T | NA | NA | NA |  |  |  |  | NA | NA | NA |  |  |  |  |  | 11 | 0,250 | -0,128 |  |  |  |  |
|  | TT |  | NA |  |  |  |  |  |  | NA |  |  |  |  |  |  |  | 1 |  |  |  |  |  |  |
| **SNP11** | GG | G | NA | NA | NA | NA | NA | NA | NA | NA | NA | NA | NA | | NA | NA | NA | 3 | 0,308 | 0,097 | 0,360 | 0,385 | 0,434 | 52 |
|  | TG | T | NA | NA | NA |  |  |  |  | NA | NA | NA |  |  |  |  |  | 10 | 0,692 | 0,097 |  |  |  |  |
|  | TT |  | NA |  |  |  |  |  |  | NA |  |  |  |  |  |  |  | 13 |  |  |  |  |  |  |
| **SNP12** | CC | C | NA | NA | NA | NA | NA | NA | NA | NA | NA | NA | NA | | NA | NA | NA | 1 | 0,154 | 0,114 | 0,449 | 0,231 | 0,266 | 52 |
|  | TC | T | NA | NA | NA |  |  |  |  | NA | NA | NA |  |  |  |  |  | 6 | 0,847 | 0,114 |  |  |  |  |
|  | TT |  | NA |  |  |  |  |  |  | NA |  |  |  |  |  |  |  | 19 |  |  |  |  |  |  |
| **SNP13** | CC | C | NA | NA | NA | NA | NA | NA | NA | NA | NA | NA | NA | | NA | NA | NA | 0 | 0,019 | -0,020 | 0,000 | 0,039 | 0,039 | 52 |
|  | TC | T | NA | NA | NA |  |  |  |  | NA | NA | NA |  |  |  |  |  | 1 | 0,981 | -0,020 |  |  |  |  |
|  | TT |  | NA |  |  |  |  |  |  | NA |  |  |  |  |  |  |  | 25 |  |  |  |  |  |  |
| **SNP14** | AA | A | NA | NA | NA | NA | NA | NA | NA | NA | NA | NA | NA | | NA | NA | NA | 8 | 0,580 | -0,067 | 0,055 | 0,520 | 0,497 | 50 |
|  | GA | G | NA | NA | NA |  |  |  |  | NA | NA | NA |  |  |  |  |  | 13 | 0,420 | -0,067 |  |  |  |  |
|  | GG |  | NA |  |  |  |  |  |  | NA |  |  |  |  |  |  |  | 4 |  |  |  |  |  |  |
| **SNP15** | AA | A | 0 | 0 | 0 | x | 0 | 0 | 48 | 0 | 0,000 | 0,000 | x | | 0,000 | 0,000 | 56 | 0 | 0,000 | 0,000 | x | 0,000 | 0,000 | 52 |
|  | GA | G | 0 | 1 | 0 |  |  |  |  | 0 | 1,000 |  |  |  |  |  |  | 0 | 1,000 | 0,000 |  |  |  |  |
|  | GG |  | 24 |  |  |  |  |  |  | 28 |  |  |  |  |  |  |  | 26 |  |  |  |  |  |  |
| **SNP16** | AA | A | 0 | 0 | 0 | x | 0 | 0 | 56 | 0 | 0,019 | -0,180 | 0,000 | | 0,037 | 0,037 | 54 | 0 | 0,000 | 0,000 | x | 0,000 | 0,000 | 53 |
|  | GA | G | 0 | 1 | 0 |  |  |  |  | 1 | 0,982 | -0,180 |  |  |  |  |  | 0 | 2,000 | 0,000 |  |  |  |  |
|  | GG |  | 28 |  |  |  |  |  |  | 26 |  |  |  |  |  |  |  | 26 |  |  |  |  |  |  |
| **SNP17** | CC | C | 12 | 0,816 | -0,226 | 0,813 | 0,368 | 0,309 | 38 | 21 | 0,978 | -0,223 | 0,000 | | 0,046 | 0,046 | 47 | 13 | 0,692 | 0,097 | 0,360 | 0,385 | 0,434 | 54 |
|  | GC | G | 7 | 0,184 | -0,226 |  |  |  |  | 1 | 0,923 | -0,223 |  |  |  |  |  | 10 | 0,308 | 0,972 |  |  |  |  |
|  | GG |  | 0 |  |  |  |  |  |  | 0 |  |  |  |  |  |  |  | 3 |  |  |  |  |  |  |
|  |  |  |  |  |  |  |  |  |  |  |  |  |  | |  |  |  |  |  |  |  |  |  |  |
|  |  |  | **POP 7: BABOLNA TETRA** | | | | | | | **POP 8: ILE DE FRANCE** | | | | | | | | **POP 9: HUNGARIAN TSIGAI** | | | | | | |
|  | GENOTYPE | ALLELE | Genotype Freq | Allele Freq | Fis | 𝑥² | H obs | H exp | n | Genotype Freq | Allele Freq | Fis | 𝑥² | | H obs | H exp | n | Genotype Freq | Allele Freq | Fis | 𝑥² | H obs | H exp | n |
| **SNP1** | CC | C | 4 | 0,361 | -0,084 | 0,175 | 0,500 | 0,468 | 72 | 4 | 0,429 | 0,028 | 0,057 | | 0,476 | 0,502 | 42 | 8 | 0,561 | -0,292 | 2,522 | 0,635 | 0,500 | 66 |
|  | TC | T | 18 | 0,639 | -0,084 |  |  |  |  | 10 | 0,571 | 0,028 |  |  |  |  |  | 21 | 0,439 | -0,292 |  |  |  |  |
|  | TT |  | 14 |  |  |  |  |  |  | 7 |  |  |  |  |  |  |  | 4 |  |  |  |  |  |  |
| **SNP2** | CC | C | 17 | 0,681 | 0,042 | 0,113 | 0,417 | 0,441 | 72 | 14 | 0,786 | 0,293 | 2,226 | | 0,238 | 0,345 | 42 | 11 | 0,567 | 0,185 | 1,232 | 0,400 | 0,499 | 60 |
|  | GC | G | 15 | 0,319 | 0,042 |  |  |  |  | 5 | 0,214 | 0,293 |  |  |  |  |  | 12 | 0,433 | 0,185 |  |  |  |  |
|  | GG |  | 4 |  |  |  |  |  |  | 2 |  |  |  |  |  |  |  | 7 |  |  |  |  |  |  |
| **SNP3** | AA | A | 0 | 0,931 | 0,075 | 0,158 | 0,139 | 0,131 | 72 | 0 | 0,976 | -0,024 | 0,000 | | 0,048 | 0,048 | 42 | 0 | 0,906 | -0,103 | 0,281 | 0,188 | 0,173 | 64 |
|  | CA | C | 5 | 0,069 | 0,075 |  |  |  |  | 1 | 0,024 | -0,024 |  |  |  |  |  | 6 | 0,938 | -0,103 |  |  |  |  |
|  | CC |  | 51 |  |  |  |  |  |  | 20 |  |  |  |  |  |  |  | 26 |  |  |  |  |  |  |
| **SNP4** | AA | A | 18 | 0,735 | -0,058 | 0,064 | 0,412 | 0,395 | 68 | 15 | 0,810 | 0,382 | 3,712 | | 0,191 | 0,316 | 42 | 11 | 0,578 | 0,039 | 0,097 | 0,469 | 0,496 | 64 |
|  | GA | G | 14 | 0,265 | -0,058 |  |  |  |  | 4 | 0,191 | 0,382 |  |  |  |  |  | 15 | 0,422 | 0,039 |  |  |  |  |
|  | GG |  | 2 |  |  |  |  |  |  | 2 |  |  |  |  |  |  |  | 6 |  |  |  |  |  |  |
| **SNP5** | GG | G | 0 | 0,069 | -0,075 | 0,158 | 0,139 | 0,131 | 72 | 0 | 0s,024 | -0,024 | 0,000 | | 0,048 | 0,048 | 42 | 0 | 0,106 | -0,119 | 0,393 | 0,212 | 0,193 | 66 |
|  | TG | T | 5 | 0,931 | -0,075 |  |  |  |  | 1 | 0,976 | -0,024 |  |  |  |  |  | 7 | 0,894 | -0,119 |  |  |  |  |
|  | TT |  | 31 |  |  |  |  |  |  | 20 |  |  |  |  |  |  |  | 26 |  |  |  |  |  |  |
| **SNP6** | AA | A | 1 | 0,300 | -0,293 | 2,737 | 0,543 | 0,426 | 70 | 3 | 0,452 | -0,025 | 1,065 | | 0,619 | 0,508 | 42 | 0 | 0,036 | -0,037 | 0,019 | 0,714 | 0,070 | 56 |
|  | GA | G | 19 | 0,700 | -0,293 |  |  |  |  | 13 | 0,548 | -0,025 |  | |  |  |  | 2 | 0,964 | -0,037 |  |  |  |  |
|  | GG |  | 15 |  |  |  |  |  |  | 5 |  |  |  | |  |  |  | 26 |  |  |  |  |  |  |
| **SNP7** | GG | G | 8 | 0,514 | -0,167 | 0,848 | 0,583 | 0,507 | 72 | 4 | 0,333 | 0,357 | 3,098 | | 0,286 | 0,455 | 42 | 9 | 0,454 | 0,027 | 2,620 | 0,364 | 0,504 | 66 |
|  | TG | T | 21 | 0,486 | -0,167 |  |  |  |  | 6 | 0,667 | 0,357 |  | |  |  |  | 12 | 0,964 | 0,027 |  |  |  |  |
|  | TT |  | 7 |  |  |  |  |  |  | 11 |  |  |  | |  |  |  | 12 |  |  |  |  |  |  |
| **SNP8** | CC | C | 1 | 0,235 | -0,155 | 0,732 | 0,417 | 0,366 | 72 | 1 | 0,214 | 0,010 | 0,026 | | 0,333 | 0,345 | 42 | 6 | 0,313 | 0,418 | 6,095* | 0,250 | 0,437 | 64 |
|  | CG | G | 15 | 0,764 | -0,155 |  |  |  |  | 7 | 0,786 | 0,010 |  |  |  |  |  | 8 | 0,688 | 0,418 |  |  |  |  |
|  | GG |  | 20 |  |  |  |  |  |  | 13 |  |  |  |  |  |  |  | 18 |  |  |  |  |  |  |
| **SNP9** | CC | C | 36 | 1,000 | 0,000 | x | 0,000 | 0,000 | 72 | 20 | 0,952 | 1,000 | 41,026* | | 0,000 | 0,093 | 42 | 30 | 0,909 | 1,000 | 39,051* | 0,000 | 0,168 | 66 |
|  | GC | G | 0 | 0,000 | 0,000 |  |  |  |  | 0 | 0,048 | 1,000 |  |  |  |  |  | 0 | 0,091 | 1,000 |  |  |  |  |
|  | GG |  | 0 |  |  |  |  |  |  | 1 |  |  |  |  |  |  |  | 3 |  |  |  |  |  |  |
| **SNP10** | GG | G | 21 | 0,729 | 0,350 | 4,721* | 0,257 | 0,401 | 70 | 7 | 0,595 | 0,213 | 0,083 | | 0,524 | 0,494 | 42 | 18 | 0,719 | 0,227 | 1,919 | 0,313 | 0,411 | 64 |
|  | TG | T | 9 | 0,271 | 0,350 |  |  |  |  | 11 | 0,405 | 0,213 |  |  |  |  |  | 10 | 0,281 | 0,227 |  |  |  |  |
|  | TT |  | 5 |  |  |  |  |  |  | 3 |  |  |  |  |  |  |  | 4 |  |  |  |  |  |  |
| **SNP11** | GG | G | 0 | 0,056 | -0,059 | 0,092 | 0,111 | 0,106 | 72 | 2 | 0,238 | -0,129 | 1,227 | | 0,286 | 0,372 | 40 | 0 | 0,076 | -0,082 | 0,175 | 0,152 | 0,142 | 66 |
|  | TG | T | 4 | 0,944 | -0,059 |  |  |  |  | 6 | 0,762 | -0,129 |  |  |  |  |  | 5 | -0,924 | -0,082 |  |  |  |  |
|  | TT |  | 32 |  |  |  |  |  |  | 13 |  |  |  |  |  |  |  | 28 |  |  |  |  |  |  |
| **SNP12** | CC | C | 1 | 0,208 | -0,095 | 0,241 | 0,361 | 0,335 | 72 | 0 | 0,071 | -0,077 | 0,081 | | 0,143 | 0,136 | 42 | 9 | 0,550 | -0,010 | 0,001 | 0,500 | 0,503 | 60 |
|  | TC | T | 13 | 0,792 | -0,095 |  |  |  |  | 3 | 0,691 | -0,077 |  |  |  |  |  | 15 | 0,450 | -0,010 |  |  |  |  |
|  | TT |  | 22 |  |  |  |  |  |  | 18 |  |  |  |  |  |  |  | 6 |  |  |  |  |  |  |
| **SNP13** | CC | C | 0 | 0,000 | 0,000 | 0,000 | 0,000 | 0,000 | 72 | 1 | 0,310 | -0,226 | 0,865 | | 0,524 | 0,438 | 42 | 0 | 0,032 | -0,033 | 0,017 | 0,065 | 0,064 | 62 |
|  | TC | T | 0 | 1,000 | 0,000 |  |  |  |  | 11 | 0,691 | -0,226 |  |  |  |  |  | 2 | 0,968 | -0,033 |  |  |  |  |
|  | TT |  | 36 |  |  |  |  |  |  | 9 |  |  |  |  |  |  |  | 29 |  |  |  |  |  |  |
| **SNP14** | AA | A | 1 | 0,439 | 0,077 | 0,284 | 0,455 | 0,500 | 66 | 1 | 0,310 | -0,226 | 0,865 | | 0,524 | 0,438 | 42 | 4 | 0,364 | -0,048 | 0,034 | 0,485 | 0,470 | 66 |
|  | GA | G | 11 | 0,561 | 0,077 |  |  |  |  | 11 | 0,691 | -0,226 |  |  |  |  |  | 16 | 0,636 | -0,048 |  |  |  |  |
|  | GG |  | 9 |  |  |  |  |  |  | 9 |  |  |  |  |  |  |  | 13 |  |  |  |  |  |  |
| **SNP15** | AA | A | 0 | 0,028 | -0,029 | 0,014 | 0,056 | 0,055 | 72 | 0 | 0,000 | 0,000 | x | | 0,000 | 0,000 | 42 | 0 | 0,167 | -0,200 | 1,185 | 0,333 | 0,282 | 66 |
|  | GA | G | 2 | 0,972 | -0,029 |  |  |  |  | 0 | 1,000 | 0,000 |  |  |  |  |  | 11 | 0,833 | -0,200 |  |  |  |  |
|  | GG |  | 34 |  |  |  |  |  |  | 21 |  |  |  |  |  |  |  | 22 |  |  |  |  |  |  |
| **SNP16** | AA | A | 0 | 0,083 | -0,091 | 0,244 | 0,167 | 0,155 | 72 | 0 | 0,050 | -0,053 | 0,027 | | 0,100 | 0,097 | 40 | 0 | 0,000 | 0,000 | x | 0,000 | 0,000 | 66 |
|  | GA | G | 2 | 0,917 | -0,091 |  |  |  |  | 2 | 0,950 | -0,053 |  |  |  |  |  | 0 | 1,000 | 0,000 |  |  |  |  |
|  | GG |  | 18 |  |  |  |  |  |  | 18 |  |  |  |  |  |  |  | 33 |  |  |  |  |  |  |
| **SNP17** | CC | C | 16 | 0,958 | -0,044 | 0,045 | 0,083 | 0,081 | 72 | 16 | 0,881 | -0,135 | 0,300 | | 0,238 | 0,215 | 42 | 10 | 0,546 | 0,022 | 0,047 | 0,485 | 0,504 | 66 |
|  | GC | G | 5 | 0,042 | -0,044 |  |  |  |  | 5 | 0,119 | -0,135 |  |  |  |  |  | 16 | 0,455 | 0,022 |  |  |  |  |
|  | GG |  | 0 |  |  |  |  |  |  | 0 |  |  |  |  |  |  |  | 7 |  |  |  |  |  |  |
|  |  |  |  |  |  |  |  |  |  |  |  |  |  | |  |  |  |  |  |  |  |  |  | |
|  |  |  | **POP 10: HUNGARIAN** **RACKA** | | | | | | | **POP 11: BOTOSANI KARAKUL** | | | | | | | | **POP 12: ROMANIAN RACKA** | | | | | | |
|  | GENOTYPE | ALLELE | Genotype Freq | Allele Freq | Fis | 𝑥² | H obs | H exp | n | Genotype Freq | Allele Freq | Fis | 𝑥² | | H obs | H exp | n | Genotype Freq | Allele Freq | Fis | 𝑥² | H obs | H exp | n |
| **SNP1** | CC | C | 8 | 0,333 | 0,250 | 3,276 | 0,333 | 0,449 | 96 | 2 | 0,265 | 0,244 | 1,333 | | 0,294 | 0,401 | 34 | 14 | 0,525 | -0,183 | 1,867 | 0,590 | 0,503 | 112 |
|  | TC | T | 16 | 0,667 | 0,250 |  |  |  |  | 5 | 0,735 | 0,244 |  |  |  |  |  | 36 | 0,475 | -0,183 |  |  |  |  |
|  | TT |  | 24 |  |  |  |  |  |  | 10 |  |  |  |  |  |  |  | 11 |  |  |  |  |  |  |
| **SNP2** | CC | C | 16 | 0,610 | 0,078 | 0,332 | 0,439 | 0,482 | 82 | 3 | 0,667 | 0,250 | 0,744 | | 0,333 | 0,485 | 12 | 39 | 0,814 | -0,006 | 0,000 | 0,305 | 0,306 | 118 |
|  | GC | G | 18 | -0,390 | 0,078 |  |  |  |  | 2 | 0,333 | 0,250 |  |  |  |  |  | 18 | 0,186 | -0,006 |  |  |  |  |
|  | GG |  | 7 |  |  |  |  |  |  | 1 |  |  |  |  |  |  |  | 2 |  |  |  |  |  |  |
| **SNP3** | AA | A | 0 | 0,969 | -0,032 | 0,033 | 0,063 | 0,061 | 96 | 0 | 0,917 | -0,091 | 0,097 | | 0,167 | 0,157 | 36 | 2 | 0,879 | 0,166 | 1,962 | 0,177 | 0,214 | 124 |
|  | CA | C | 3 | 0,031 | -0,032 |  |  |  |  | 3 | 0,083 | -0,091 |  |  |  |  |  | 11 | 0,121 | 0,166 |  |  |  |  |
|  | CC |  | 45 |  |  |  |  |  |  | 15 |  |  |  |  |  |  |  | 49 |  |  |  |  |  |  |
| **SNP4** | AA | A | 24 | 0,734 | -0,144 | 0,849 | 0,447 | 0,395 | 94 | 5 | 0,636 | 0,214 | 0,769 | | 0,364 | 0,485 | 22 | 42 | 0,836 | -0,077 | 0,292 | 0,295 | 0,276 | 122 |
|  | GA | G | 21 | 0,266 | -0,144 |  |  |  |  | 4 | 0,364 | 0,214 |  |  |  |  |  | 18 | 0,164 | -0,077 |  |  |  |  |
|  | GG |  | 2 |  |  |  |  |  |  | 2 |  |  |  |  |  |  |  | 1 |  |  |  |  |  |  |
| **SNP5** | GG | G | 0 | 0,031 | -0,032 | 0,032 | 0,063 | 0,061 | 96 | 0 | 0,115 | -0,130 | 0,142 | | 0,231 | 0,212 | 26 | 2 | 0,121 | 0,166 | 1,962 | 0,177 | 0,214 | 124 |
|  | TG | T | 3 | 0,969 | -0,032 |  |  |  |  | 3 | 0,884 | -0,130 |  |  |  |  |  | 11 | 0,879 | 0,166 |  |  |  |  |
|  | TT |  | 45 |  |  |  |  |  |  | 10 |  |  |  |  |  |  |  | 49 |  |  |  |  |  |  |
| **SNP6** | AA | A | 12 | 0,476 | 0,236 | 2,592 | 0,381 | 0,505 | 94 | 0 | 0,000 | 0,000 | 0,000 | | 0,000 | 0,000 | 12 | 16 | 0,451 | 0,239 | 3,717 | 0,377 | 0,499 | 122 |
|  | GA | G | 16 | 0,524 | 0,236 |  |  |  |  | 0 | 1,000 | 0,000 |  |  |  |  |  | 23 | 0,549 | 0,239 |  |  |  |  |
|  | GG |  | 14 |  |  |  |  |  |  | 6 |  |  |  |  |  |  |  | 22 |  |  |  |  |  |  |
| **SNP7** | GG | G | 26 | 0,719 | 0,124 | 0,878 | 0,354 | 0,409 | 96 | 7 | 0,531 | 0,624 | 6,892* | | 0,119 | 0,514 | 32 | 7 | 0,367 | -0,077 | 0,279 | 0,500 | 0,468 | 120 |
|  | TG | T | 17 | 0,281 | 0,124 |  |  |  |  | 3 | 0,469 | 0,624 |  |  |  |  |  | 30 | 0,633 | -0,077 |  |  |  |  |
|  | TT |  | 5 |  |  |  |  |  |  | 6 |  |  |  |  |  |  |  | 23 |  |  |  |  |  |  |
| **SNP8** | CC | C | 2 | 0,229 | 1,000 | 0,127 | 0,375 | 0,357 | 96 | 2 | 0,167 | 0,760 | 10,752* | | 0,067 | 0,287 | 30 | 5 | 0,328 | -0,116 | 0,709 | 0,492 | 0,444 | 122 |
|  | CG | G | 18 | 0,771 | 1,000 |  |  |  |  | 1 | 0,833 | 0,760 |  |  |  |  |  | 30 | 0,672 | -0,116 |  |  |  |  |
|  | GG |  | 28 |  |  |  |  |  |  | 12 |  |  |  |  |  |  |  | 26 |  |  |  |  |  |  |
| **SNP9** | CC | C | 39 | 0,830 | 1,000 | 49,704* | 0,000 | 0,286 | 94 | 14 | 0,933 | 1,000 | 29,037* | | 0,000 | 0,287 | 31 | 61 | 1,000 | 0,000 | x | 0,000 | 0,000 | 122 |
|  | GC | G | 0 | 0,170 | 1,000 |  |  |  |  | 0 | 0,067 | 1,000 |  |  |  |  |  | 0 | 0,000 | 0,000 |  |  |  |  |
|  | GG |  | 8 |  |  |  |  |  |  | 1 |  |  |  |  |  |  |  | 0 |  |  |  |  |  |  |
| **SNP10** | GG | G | 18 | 0,646 | -0,184 | 1,451 | 0,542 | 0,462 | 96 | 11 | 0,833 | 0,280 | 1,685 | | 0,200 | 0,287 | 32 | 14 | 0,524 | -0,196 | 2,196 | 0,597 | 0,503 | 124 |
|  | TG | T | 26 | 0,354 | -0,184 |  |  |  |  | 3 | 0,167 | 0,280 |  |  |  |  |  | 37 | 0,476 | -0,196 |  |  |  |  |
|  | TT |  | 4 |  |  |  |  |  |  | 1 |  |  |  |  |  |  |  | 11 |  |  |  |  |  |  |
| **SNP11** | GG | G | 0 | 0,073 | -0,079 | 0,252 | 0,146 | 0,137 | 96 | 2 | 0,083 | 1,000 | 31,380* | | 0,000 | 0,156 | 48 | 0 | 0,117 | -0,132 | 0,964 | 0,233 | 0,208 | 120 |
|  | TG | T | 7 | 0,927 | -0,079 |  |  |  |  | 0 | 0,917 | 1,000 |  |  |  |  |  | 14 | 0,883 | -0,132 |  |  |  |  |
|  | TT |  | 41 |  |  |  |  |  |  | 22 |  |  |  |  |  |  |  | 46 |  |  |  |  |  |  |
| **SNP12** | CC | C | 4 | 0,351 | -0,167 | 1,159 | 0,532 | 0,461 | 94 | 0 | 0,091 | -0,100 | 0,053 | | 0,182 | 0,173 | 22 | 12 | 0,444 | -0,013 | 0,001 | 0,500 | 0,498 | 124 |
|  | TC | T | 25 | 0,650 | -0,167 |  |  |  |  | 2 | 0,909 | -0,100 |  |  |  |  |  | 31 | 0,557 | -0,013 |  |  |  |  |
|  | TT |  | 18 |  |  |  |  |  |  | 9 |  |  |  |  |  |  |  | 19 |  |  |  |  |  |  |
| **SNP13** | CC | C | 0 | 0,000 | 0,000 | x | 0,000 | 0,000 | 96 | 0 | 0,083 | -0,100 | 0,000 | | 0,167 | 0,167 | 12 | 0 | 0,008 | -0,008 | 0,000 | 0,016 | 0,016 | 122 |
|  | TC | T | 0 | 1,000 | 0,000 |  |  |  |  | 1 | 0,917 | -0,100 |  |  |  |  |  | 1 | 0,992 | -0,008 |  |  |  |  |
|  | TT |  | 48 |  |  |  |  |  |  | 5 |  |  |  |  |  |  |  | 60 |  |  |  |  |  |  |
| **SNP14** | AA | A | 3 | 0,240 | 0,028 | 0,071 | 0,354 | 0,368 | 96 | 2 | 0,409 | -0,091 | 0,128 | | 0,455 | 0,507 | 22 | 18 | 0,508 | 0,129 | 1,162 | 0,436 | 0,504 | 124 |
|  | GA | G | 17 | 0,760 | 0,028 |  |  |  |  | 5 | 0,591 | -0,091 |  |  |  |  |  | 27 | 0,492 | 0,129 |  |  |  |  |
|  | GG |  | 28 |  |  |  |  |  |  | 4 |  |  |  |  |  |  |  | 17 |  |  |  |  |  |  |
| **SNP15** | AA | A | 0 | 0,208 | -0,263 | 3,133 | 0,417 | 0,333 | 96 | 0 | 0,000 | 0,060 | x | | 0,000 | 0,000 | 24 | 0 | 0,057 | -0,060 | 0,189 | 0,113 | 0,107 | 124 |
|  | GA | G | 20 | 0,760 | -0,263 |  |  |  |  | 0 | 1,000 | 0,060 |  |  |  |  |  | 7 | 0,944 | -0,060 |  |  |  |  |
|  | GG |  | 28 |  |  |  |  |  |  | 12 |  |  |  |  |  |  |  | 55 |  |  |  |  |  |  |
| **SNP16** | AA | A | 0 | 0,000 |  | x | 0,000 | 0,000 | 96 | 2 | 0,250 | 0,429 | 3,210 | | 0,214 | 0,389 | 28 | 0 | 0,032 | -0,033 | 0,051 | 0,065 | 0,063 | 124 |
|  | GA | G | 0 | 1,000 |  |  |  |  |  | 3 | 0,750 | 0,429 |  |  |  |  |  | 4 | 0,968 | -0,033 |  |  |  |  |
|  | GG |  | 48 |  |  |  |  |  |  | 9 |  |  |  |  |  |  |  | 58 |  |  |  |  |  |  |
| **SNP17** | CC | C | 33 | 0,843 | 0,185 | 1,523 | 0,313 | 0,266 | 96 | 13 | 1,000 | 0,000 | x | | 0,000 | 0,000 | 26 | 49 | 0,887 | 0,034 | 0,114 | 0,194 | 0,202 | 124 |
|  | GC | G | 15 | 0,156 | 0,185 |  |  |  |  | 0 | 0,000 | 0,000 |  |  |  |  |  | 12 | 0,113 | 0,034 |  |  |  |  |
|  | GG |  | 0 |  |  |  |  |  |  | 0 |  |  |  |  |  |  |  | 1 |  |  |  |  |  |  |
|  |  | |  |  |  |  |  |  |  |  |  |  |  | |  |  |  |  |  |  |  |  |  |  |
|  |  | | **POP 13: TRANSYLVANIAN MERINO** | | | | | | | **POP 14: ROMANIAN TSIGAI** | | | | | | | | **POP 15: TURCANA** | | | | | | |
|  | GENOTYPE | ALLELE | Genotype Freq | Allele Freq | Fis | 𝑥² | H obs | H exp | n | Genotype Freq | Allele Freq | Fis | 𝑥² | | H obs | H exp | n | Genotype Freq | Allele Freq | Fis | 𝑥² | H obs | H exp | n |
| **SNP1** | CC | C | 20 | 0,655 | 0,210 | 2,083 | 0,357 | 0,458 | 84 | 1 | 0,367 | -0,292 | 1,006 | | 0,600 | 0,461 | 30 | 8 | 0,412 | -0,122 | 0,734 | 0,544 | 0,489 | 114 |
|  | TC | T | 15 | 0,345 | 0,210 |  |  |  |  | 9 | 0,633 | -0,292 |  |  |  |  |  | 31 | 0,588 | -0,122 |  |  |  |  |
|  | TT |  | 7 |  |  |  |  |  |  | 5 |  |  |  |  |  |  |  | 18 |  |  |  |  |  |  |
| **SNP2** | CC | C | 11 | 0,571 | -0,050 | 0,044 | 0,514 | 0,497 | 70 | 5 | 0,682 | -0,048 | 0,000 | | 0,455 | 0,455 | 22 | 32 | 0,763 | -0,116 | 0,666 | 0,404 | 0,365 | 11 |
|  | GC | G | 8 | 0,429 | -0,050 |  |  |  |  | 5 | 0,318 | -0,048 |  |  |  |  |  | 23 | 0,237 | -0,116 |  |  |  |  |
|  | GG |  | 6 |  |  |  |  |  |  | 1 |  |  |  |  |  |  |  | 2 |  |  |  |  |  |  |
| **SNP3** | AA | A | 9 | 0,977 | -0,023 | 0,012 | 0,046 | 0,050 | 88 | 0 | 1,000 | 0,000 | x | | 0,000 | 0,000 | 34 | 0 | 0,9386 | -0,654 | 0,207 | 0,123 | 0,116 | 114 |
|  | CA | C | 2 | 0,023 | -0,023 |  |  |  |  | 0 | 0,000 | 0,000 |  |  |  |  |  | 7 | 0,614 | -0,654 |  |  |  |  |
|  | CC |  | 42 |  |  |  |  |  |  | 17 |  |  |  |  |  |  |  | 50 |  |  |  |  |  |  |
| **SNP4** | AA | A | 12 | 0,566 | -0,018 | 0,001 | 0,500 | 0,498 | 76 | 7 | 0,692 | 0,278 | 1,355 | | 0,308 | 0,443 | 26 | 37 | 0,810 | -0,122 | 0,759 | 0,345 | 0,310 | 116 |
|  | GA | G | 19 | 0,434 | -0,018 |  |  |  |  | 4 | 0,308 | 0,278 |  |  |  |  |  | 20 | 0,190 | -0,122 |  |  |  |  |
|  | GG |  | 7 |  |  |  |  |  |  | 2 |  |  |  |  |  |  |  | 1 |  |  |  |  |  |  |
| **SNP5** | GG | G | 0 | 0,012 | -0,012 | 0,000 | 0,024 | 0,024 | 84 | 0 | 0,000 | 0,000 | x | | 0,000 | 0,000 | 32 | 0 | 0,060 | -0,064 | 0,203 | 0,121 | 0,114 | 116 |
|  | TG | T | 1 | 0,988 | -0,012 |  |  |  |  | 0 | 1,000 | 0,000 |  |  |  |  |  | 7 | 0,940 | -0,064 |  |  |  |  |
|  | TT |  | 41 |  |  |  |  |  |  | 16 |  |  |  |  |  |  |  | 51 |  |  |  |  |  |  |
| **SNP6** | AA | A | 0 | 0,074 | -0,079 | 0,169 | 0,147 | 0,138 | 68 | 3 | 0,318 | 0,791 | 8,000* | | 0,091 | 0,455 | 22 | 2 | 0,164 | 0,056 | 0,249 | 0,259 | 0,276 | 116 |
|  | GA | G | 5 | 0,927 | -0,079 |  |  |  |  | 1 | 0,682 | 0,791 |  |  |  |  |  | 15 | 0,183 | 0,056 |  |  |  |  |
|  | GG |  | 29 |  |  |  |  |  |  | 7 |  |  |  |  |  |  |  | 41 |  |  |  |  |  |  |
| **SNP7** | GG | G | 5 | 0,361 | -0,059 | 0,097 | 0,488 | 0,467 | 86 | 2 | 0,158 | 0,042 | 8,422* | | 0,105 | 0,273 | 38 | 7 | 0,272 | 0,247 | 3,761 | 0,298 | 0,400 | 114 |
|  | TG | T | 21 | 0,640 | -0,059 |  |  |  |  | 2 | 0,842 | 0,042 |  |  |  |  |  | 17 | 0,728 | 0,247 |  |  |  |  |
|  | TT |  | 17 |  |  |  |  |  |  | 15 |  |  |  |  |  |  |  | 33 |  |  |  |  |  |  |
| **SNP8** | CC | C | 2 | 0,140 | 0,225 | 2,548 | 0,186 | 0,243 | 86 | 3 | 0,375 | 0,200 | 0,872 | | 0,375 | 0,484 | 40 | 9 | 0,371 | 0,076 | 0,419 | 0,431 | 0,471 | 116 |
|  | CG | G | 8 | 0,861 | 0,225 |  |  |  |  | 6 | 0,625 | 0,200 |  |  |  |  |  | 25 | 0,629 | 0,076 |  |  |  |  |
|  | GG |  | 33 |  |  |  |  |  |  | 7 |  |  |  |  |  |  |  | 24 |  |  |  |  |  |  |
| **SNP9** | CC | C | 39 | 0,975 | 1,000 | 79,013* | 0,000 | 0,049 | 80 | 20 | 1,000 | 0,000 | x | | 0,000 | 0,000 | 40 | 53 | 0,930 | 1,000 | 64,610* | 0,000 | 0,132 | 114 |
|  | GC | G | 0 | 0,025 | 1,000 |  |  |  |  | 0 | 0,000 | 0,000 |  |  |  |  |  | 0 | 0,070 | 1,000 |  |  |  |  |
|  | GG |  | 1 |  |  |  |  |  |  | 0 |  |  |  |  |  |  |  | 4 |  |  |  |  |  |  |
| **SNP10** | GG | G | 30 | 0,807 | 0,198 | 1,988 | 0,250 | 0,515 | 88 | 11 | 0,775 | -0,290 | 1,471 | | 0,450 | 0,358 | 40 | 26 | 0,655 | 0,084 | 0,503 | 0,414 | 0,456 | 116 |
|  | TG | T | 11 | 0,1932 | 0,198 |  |  |  |  | 9 | 0,225 | -0,290 |  |  |  |  |  | 24 | 0,345 | 0,084 |  |  |  |  |
|  | TT |  | 3 |  |  |  |  |  |  | 7 |  |  |  |  |  |  |  | 8 |  |  |  |  |  |  |
| **SNP11** | GG | G | 4 | 0,275 | 0,122 | 0,728 | 0,350 | 0,404 | 80 | 0 | 0,056 | -0,059 | 0,030 | | 0,111 | 0,108 | 36 | 2 | 0,098 | 0,294 | 5,523* | 0,125 | 0,179 | 112 |
|  | TG | T | 14 | 0,725 | 0,122 |  |  |  |  | 2 | 0,944 | -0,059 |  |  |  |  |  | 7 | 0,902 | 0,294 |  |  |  |  |
|  | TT |  | 22 |  |  |  |  |  |  | 16 |  |  |  |  |  |  |  | 47 |  |  |  |  |  |  |
| **SNP12** | CC | C | 1 | 0,060 | 0,362 | 7,092* | 0,071 | 0,113 | 84 | 0 | 0,125 | -0,143 | 0,238 | | 0,250 | 0,226 | 32 | 1 | 0,147 | -0,034 | 0,038 | 0,259 | 0,252 | 116 |
|  | TC | T | 3 | 0,9405 | 0,362 |  |  |  |  | 4 | 0,875 | -0,143 |  |  |  |  |  | 15 | 0,853 | -0,034 |  |  |  |  |
|  | TT |  | 38 |  |  |  |  |  |  | 12 |  |  |  |  |  |  |  | 42 |  |  |  |  |  |  |
| **SNP13** | CC | C | 1 | 0,071 | 0,282 | 4,208* | 0,095 | 0,134 | 84 | 0 | 0,000 | 0,000 | x | | 0,000 | 0,000 | 26 | 1 | 0,089 | 0,122 | 1,034 | 0,143 | 0,164 | 112 |
|  | TC | T | 4 | 0,9286 | 0,282 |  |  |  |  | 0 | 1,000 | 0,000 |  |  |  |  |  | 8 | 0,911 | 0,122 |  |  |  |  |
|  | TT |  | 37 |  |  |  |  |  |  | 13 |  |  |  |  |  |  |  | 47 |  |  |  |  |  |  |
| **SNP14** | AA | A | 12 | 0,544 | -0,120 | 0,532 | 0,556 | 0,502 | 90 | 5 | 0,633 | -0,292 | 1,006 | | 0,600 | 0,480 | 30 | 11 | 0,431 | 0,016 | 0,035 | 0,483 | 0,495 | 116 |
|  | GA | G | 25 | 0,4556 | -0,120 |  |  |  |  | 9 | 0,367 | -0,292 |  |  |  |  |  | 28 | 0,569 | 0,016 |  |  |  |  |
|  | GG |  | 8 |  |  |  |  |  |  | 1 |  |  |  |  |  |  |  | 19 |  |  |  |  |  |  |
| **SNP15** | AA | A | 0 | 0,022 | 0,227 | 0,011 | 0,044 | 0,044 | 90 | 0 | 0,000 | 0,000 | x | | 0,000 | 0,000 | 32 | 0 | 0,139 | -0,161 | 1,301 | 0,278 | 0,241 | 108 |
|  | GA | G | 2 | 0,978 | 0,227 |  |  |  |  | 0 | 1,000 | 0,000 |  |  |  |  |  | 15 | 0,861 | -0,161 |  |  |  |  |
|  | GG |  | 43 |  |  |  |  |  |  | 16 |  |  |  |  |  |  |  | 39 |  |  |  |  |  |  |
| **SNP16** | AA | A | 0 | 0,000 | 0 | x | 0 | 0 | 90 | 0 | 0,028 | -0,286 | 0,000 | | 0,056 | 0,056 | 36 | 0 | 0,009 | -0,009 | 0,000 | 0,017 | 0,017 | 116 |
|  | GA | G | 0 | 1 | 0 |  |  |  |  | 1 | 0,972 | -0,286 |  |  |  |  |  | 1 | 0,991 | -0,009 |  |  |  |  |
|  | GG |  | 45 |  |  |  |  |  |  | 17 |  |  |  |  |  |  |  | 57 |  |  |  |  |  |  |
| **SNP17** | CC | C | 29 | 0,829 | -0,206 | 1,718 | 0,341 | 0,286 | 88 | 12 | 0,853 | -0,172 | 0,394 | | 0,294 | 0,259 | 34 | 45 | 0,884 | 0,217 | 3,014 | 0,161 | 0,207 | 112 |
|  | GC | G | 15 | 0,171 | -0,206 |  |  |  |  | 5 | 0,147 | -0,172 |  |  |  |  |  | 9 | 0,116 | 0,217 |  |  |  |  |
|  | GG |  | 0 |  |  |  |  |  |  | 0 |  |  |  |  |  |  |  | 2 |  |  |  |  |  |  |
|  |  |  |  | | | | | | |  | | | | | | | |  |  |  |  |  |  |  |
|  |  | | **POP 16: HUNGARIAN MERINO** | | | | | | | **POP 17: HUNGARIAN AWASSI** | | | | | | | |  |  |  |  |  |  |  |
|  | GENOTYPE | ALLELE | Genotype Freq | Allele Freq | Fis | 𝑥² | H obs | H exp | n | Genotype Freq | Allele Freq | Fis | 𝑥² | H obs | | H exp | n |  |  |  |  |  |  |  |
| SNP1 | CC | C | 16 | 0,686 | -0,061 | 0,075 | 0,457 | 0,437 | 70 | 4 | 0,338 | -0,062 | 0,099 | 0,475 | | 0,453 | 80 |  |  |  |  |  |  |  |
|  | TC | T | 16 | 0,314 | -0,061 |  |  |  |  | 19 | 0,662 |  |  |  |  |  |  |  |  |  |  |  |  |  |
|  | TT |  | 3 |  |  |  |  |  |  | 17 |  |  |  |  |  |  |  |  |  |  |  |  |  |  |
| SNP2 | CC | C | 11 | 0,557 | 0,016 | 0,032 | 0,486 | 0,501 | 70 | 30 | 0,875 | -0,143 | 0,727 | 0,250 | | 0,222 | 80 |  |  |  |  |  |  |  |
|  | GC | G | 17 | 0,443 | 0,016 |  |  |  |  | 10 | 0,125 |  |  |  |  |  |  |  |  |  |  |  |  |  |
|  | GG |  | 7 |  |  |  |  |  |  | 0 |  |  |  |  |  |  |  |  |  |  |  |  |  |  |
| SNP3 | AA | A | 0 | 0,000 | 0,000 | x | 0,000 | 0,000 | 70 | 3 | 0,175 | 0,307 | 4,259* | 0,200 | | 0,292 | 80 |  |  |  |  |  |  |  |
|  | CA | C | 0 | 1 | 0,000 |  |  |  |  | 8 | 0,825 |  |  |  |  |  |  |  |  |  |  |  |  |  |
|  | CC |  | 35 |  |  |  |  |  |  | 29 |  |  |  |  |  |  |  |  |  |  |  |  |  |  |
| SNP4 | AA | A | 11 | 0,588 | -0,093 | 0,649 | 0,529 | 0,492 | 68 | 30 | 0,885 | -0,130 | 0,583 | 0,231 | | 0,207 | 78 |  |  |  |  |  |  |  |
|  | GA | G | 18 | 0,412 | -0,093 |  |  |  |  | 9 | 0,115 |  |  |  |  |  |  |  |  |  |  |  |  |  |
|  | GG |  | 5 |  |  |  |  |  |  | 0 |  |  |  |  |  |  |  |  |  |  |  |  |  |  |
| SNP5 | GG | G | 0 | 0,000 | 0,000 | x | 0,000 | 0,000 | 70 | 3 | 0,175 | 0,307 | 4,259* | 0,200 | | 0,292 | 80 |  |  |  |  |  |  |  |
|  | TG | T | 0 | 1 | 0,000 |  |  |  |  | 8 | 0,825 |  |  |  |  |  |  |  |  |  |  |  |  |  |
|  | TT |  | 35 |  |  |  |  |  |  | 29 |  |  |  |  |  |  |  |  |  |  |  |  |  |  |
| SNP6 | AA | A | 2 | 0,143 | 0,300 | 3,692 | 0,171 | 0m248 | 70 | 0 | 0,063 | -0,067 | 0,141 | 0,125 | | 0,119 | 80  **Notes:**  NA = not available data;  * = deviated from HWE;  SNP1= rs161504783-*HSPA12A*;  SNP2= rs397514116-*HSP90AA1*;  SNP3= rs397514117-*HSP90AA1*;  SNP4= rs397514269 -*HSP90AA1*;  SNP5= rs397514272 -*HSP90AA1*;  SNP6= rs397514273-*HSP90AA1*;  SNP7= rs410259751-*IL33*;  SNP8= rs411181557-*DIO2*;  SNP9= rs414917134-*BTNL2*;  SNP10= rs416941267-*CSN2*;  SNP11= rs420611298-*ABCG1*;  SNP12= rs420959261-*CSN1S1*;  SNP13= rs430298704-*CSN2*;  SNP14= rs55631463-*GHR*;  SNP15= rs588145625-*HSPA8*;  SNP16= rs588498137-*STAT3*;  SNP17= rs602521720-*HCRT* |  |  |  |  |  |  |  |
|  | GA | G | 6 | 0,857 | 0,300 |  |  |  |  | 5 | 0,937 |  |  |  |  |  |  |  |  |  |  |  |  |  |
|  | GG |  | 27 |  |  |  |  |  |  | 35 |  |  |  |  |  |  |  |  |  |  |  |  |  |  |
| SNP7 | GG | G | 2 | 0,243 | -0,010 | 0,001 | 0,371 | 0,373 | 70 | 5 | 0,313 | 0,127 | 0,790 | 0,375 | | 0,435 | 80 |  |  |  |  |  |  |  |
|  | TG | T | 13 | 0,757 | -0,010 |  |  |  |  | 15 | 0,687 |  |  |  |  |  |  |  |  |  |  |  |  |  |
|  | TT |  | 20 |  |  |  |  |  |  | 20 |  |  |  |  |  |  |  |  |  |  |  |  |  |  |
| SNP8 | CC | C | 1 | 0,171 | -0,006 | 0,003 | 0,286 | 0,288 | 70 | 3 | 0,337 | -0,174 | 1,048 | 0,525 | | 0,453 | 80 |  |  |  |  |  |  |  |
|  | CG | G | 10 | 0,829 | -0,006 |  |  |  |  | 21 | 0,663 |  |  |  |  |  |  |  |  |  |  |  |  |  |
|  | GG |  | 24 |  |  |  |  |  |  | 16 |  |  |  |  |  |  |  |  |  |  |  |  |  |  |
| SNP9 | CC | C | 18 | 0,765 | -0,301 | 2,986 | 0,471 | 0,365 | 68 | 24 | 0,811 | -0,057 | 0,072 | 0,324 | | 0,311 | 74 |  |  |  |  |  |  |  |
|  | GC | G | 16 | 0,235 | -0,301 |  |  |  |  | 12 | 0,189 |  |  |  |  |  |  |  |  |  |  |  |  |  |
|  | GG |  | 0 |  |  |  |  |  |  | 1 |  |  |  |  |  |  |  |  |  |  |  |  |  |  |
| SNP10 | GG | G | 26 | 0,857 | 0,067 | 0,246 | 0,229 | 0,248 | 70 | 26 | 0,813 | -0,067 | 0,121 | 0,325 | | 0,309 | 80 |  |  |  |  |  |  |  |
|  | TG | T | 8 | 0,143 | 0,067 |  |  |  |  | 13 | 0,187 |  |  |  |  |  |  |  |  |  |  |  |  |  |
|  | TT |  | 1 |  |  |  |  |  |  | 1 |  |  |  |  |  |  |  |  |  |  |  |  |  |  |
| SNP11 | GG | G | 0 | 0,000 | 0,000 | x | 0,000 | 0,000 | 68 | 0 | 0,000 | 0,000 | x | 0,000 | | 0,000 | 80 |  |  |  |  |  |  |  |
|  | TG | T | 0 | 1 | 0,000 |  |  |  |  | 0 | 1,000 |  |  |  |  |  |  |  |  |  |  |  |  |  |
|  | TT |  | 35 |  |  |  |  |  |  | 40 |  |  |  |  |  |  |  |  |  |  |  |  |  |  |
| SNP12 | CC | C | 1 | 0,129 | 0,108 | 0,562 | 0,200 | 0,227 | 70 | 1 | 0,154 | 0,015 | 0,032 | 0,256 | | 0,264 | 78 |  |  |  |  |  |  |  |
|  | TC | T | 7 | 0,871 | 0,108 |  |  |  |  | 10 | 0,846 |  |  |  |  |  |  |  |  |  |  |  |  |  |
|  | TT |  | 27 |  |  |  |  |  |  | 28 |  |  |  |  |  |  |  |  |  |  |  |  |  |  |
| SNP13 | CC | C | 0 | 0,143 | -0,167 | 0,864 | 0,286 | 0,248 | 79 | 0 | 0,116 | -0,130 | 0,583 | 0,231 | | 0,207 | 78 |  |  |  |  |  |  |  |
|  | TC | T | 10 | 0,857 | -0,167 |  |  |  |  | 9 | 0,885 |  |  |  |  |  |  |  |  |  |  |  |  |  |
|  | TT |  | 25 |  |  |  |  |  |  | 30 |  |  |  |  |  |  |  |  |  |  |  |  |  |  |
| SNP14 | AA | A | 0 | 0,264 | -0,360 | 4,122* | 0,529 | 0,395 | 68 | 11 | 0,563 | -0,168 | 0,969 | 0,575 | | 0,498 | 80 |  |  |  |  |  |  |  |
|  | GA | G | 18 | 0,735 | -0,360 |  |  |  |  | 23 | 0,437 |  |  |  |  |  |  |  |  |  |  |  |  |  |
|  | GG |  | 16 |  |  |  |  |  |  | 6 |  |  |  |  |  |  |  |  |  |  |  |  |  |  |
| SNP15 | AA | A | 0 | 0,000 | 0,000 | x | 0,000 | 0,000 | 70 | 0 | 0,000 | 0,000 | x | 0,000 | | 0,000 | 80 |  |  |  |  |  |  |  |
|  | GA | G | 0 | 1 | 0,000 |  |  |  |  | 0 | 1,000 |  |  |  |  |  |  |  |  |  |  |  |  |  |
|  | GG |  | 35 |  |  |  |  |  |  | 40 |  |  |  |  |  |  |  |  |  |  |  |  |  |  |
| SNP16 | AA | A | 0 | 0,157 | -0,186 | 1,093 | 0,314 | 0,269 | 70 | 0 | 0,013 | -0,013 | 0,000 | 0,025 | | 0,025 | 80 |  |  |  |  |  |  |  |
|  | GA | G | 11 | 0,843 | -0,186 |  |  |  |  | 1 | 0,987 |  |  |  |  |  |  |  |  |  |  |  |  |  |
|  | GG |  | 24 |  |  |  |  |  |  | 39 |  |  |  |  |  |  |  |  |  |  |  |  |  |  |
| SNP17 | CC | C | 35 | 1 | 0,000 | x | 0,000 | 0,000 | 68 | 37 | 0,963 | -0,039 | 0,040 | 0,075 | | 0,073 | 80 |  |  |  |  |  |  |  |
|  | GC | G | 0 | 0 | 0,000 |  |  |  |  | 3 | 0,037 |  |  |  |  |  |  |  |  |  |  |  |  |  |
|  | GG |  | 0 |  |  |  |  |  |  | 0 |  |  |  |  |  |  |  |  |  |  |  |  |  |  |

Table S3. Genotype frequency of polymorphic loci

| **Breed** | **SNP** | **Genotype** | **N** |
| --- | --- | --- | --- |
| Pramenka | rs161504783 | CC | 8 |
|  |  | TC | 18 |
|  |  | TT | 6 |
|  | rs397514116 | CC | 21 |
|  |  | GC | 12 |
|  |  | GG | 0 |
|  | rs397514117 | AA | 0 |
|  |  | CA | 0 |
|  |  | CC | 54 |
|  | rs397514269 | AA | 0 |
|  |  | GA | 0 |
|  |  | GG | 0 |
|  | rs397514272 | GG | 0 |
|  |  | TG | 3 |
|  |  | TT | 27 |
|  | rs397514273 | AA | 0 |
|  |  | GA | 13 |
|  |  | GG | 16 |
|  | rs410259751 | GG | 2 |
|  |  | TG | 11 |
|  |  | TT | 17 |
|  | rs411181557 | CC | 4 |
|  |  | CG | 12 |
|  |  | GG | 18 |
|  | rs414917134 | CC | 0 |
|  |  | GC | 0 |
|  |  | GG | 0 |
|  | rs416941267 | GG | 15 |
|  |  | TG | 15 |
|  |  | TT | 4 |
|  | rs420611298 | GG | 0 |
|  |  | TG | 0 |
|  |  | TT | 0 |
|  | rs420959261 | CC | 0 |
|  |  | TC | 0 |
|  |  | TT | 0 |
|  | rs430298704 | CC | 0 |
|  |  | TC | 3 |
|  |  | TT | 23 |
|  | rs55631463 | AA | 0 |
|  |  | GA | 0 |
|  |  | GG | 22 |
|  | rs588145625 | AA | 0 |
|  |  | GA | 7 |
|  |  | GG | 26 |
|  | rs588498137 | AA | 0 |
|  |  | GA | 1 |
|  |  | GG | 35 |
|  | rs602521720 | CC | 32 |
|  |  | GC | 1 |
|  |  | GG | 0 |
| Béni Guil | rs161504783 | CC | 1 |
|  |  | TC | 22 |
|  |  | TT | 1 |
|  | rs397514116 | CC | 0 |
|  |  | GC | 0 |
|  |  | GG | 0 |
|  | rs397514117 | AA | 0 |
|  |  | CA | 0 |
|  |  | CC | 0 |
|  | rs397514269 | AA | 0 |
|  |  | GA | 0 |
|  |  | GG | 0 |
|  | rs397514272 | GG | 0 |
|  |  | TG | 0 |
|  |  | TT | 0 |
|  | rs397514273 | AA | 0 |
|  |  | GA | 0 |
|  |  | GG | 0 |
|  | rs410259751 | GG | 2 |
|  |  | TG | 24 |
|  |  | TT | 0 |
|  | rs411181557 | CC | 0 |
|  |  | CG | 0 |
|  |  | GG | 0 |
|  | rs414917134 | CC | 0 |
|  |  | GC | 0 |
|  |  | GG | 0 |
|  | rs416941267 | GG | 0 |
|  |  | TG | 0 |
|  |  | TT | 0 |
|  | rs420611298 | GG | 0 |
|  |  | TG | 0 |
|  |  | TT | 0 |
|  | rs420959261 | CC | 0 |
|  |  | TC | 0 |
|  |  | TT | 0 |
|  | rs430298704 | CC | 0 |
|  |  | TC | 0 |
|  |  | TT | 0 |
|  | rs55631463 | AA | 0 |
|  |  | GA | 0 |
|  |  | GG | 0 |
|  | rs588145625 | AA | 0 |
|  |  | GA | 0 |
|  |  | GG | 24 |
|  | rs588498137 | AA | 0 |
|  |  | GA | 0 |
|  |  | GG | 29 |
|  | rs602521720 | CC | 27 |
|  |  | GC | 0 |
|  |  | GG | 0 |
| D'man | rs161504783 | CC | 0 |
|  |  | TC | 19 |
|  |  | TT | 3 |
|  | rs397514116 | CC | 0 |
|  |  | GC | 0 |
|  |  | GG | 0 |
|  | rs397514117 | AA | 0 |
|  |  | CA | 0 |
|  |  | CC | 0 |
|  | rs397514269 | AA | 0 |
|  |  | GA | 0 |
|  |  | GG | 0 |
|  | rs397514272 | GG | 0 |
|  |  | TG | 0 |
|  |  | TT | 0 |
|  | rs397514273 | AA | 0 |
|  |  | GA | 0 |
|  |  | GG | 0 |
|  | rs410259751 | GG | 0 |
|  |  | TG | 0 |
|  |  | TT | 0 |
|  | rs411181557 | CC | 0 |
|  |  | CG | 0 |
|  |  | GG | 0 |
|  | rs414917134 | CC | 0 |
|  |  | GC | 0 |
|  |  | GG | 0 |
|  | rs416941267 | GG | 0 |
|  |  | TG | 0 |
|  |  | TT | 0 |
|  | rs420611298 | GG | 0 |
|  |  | TG | 0 |
|  |  | TT | 0 |
|  | rs420959261 | CC | 0 |
|  |  | TC | 0 |
|  |  | TT | 0 |
|  | rs430298704 | CC | 0 |
|  |  | TC | 0 |
|  |  | TT | 0 |
|  | rs55631463 | AA | 0 |
|  |  | GA | 0 |
|  |  | GG | 0 |
|  | rs588145625 | AA | 0 |
|  |  | GA | 0 |
|  |  | GG | 18 |
|  | rs588498137 | AA | 0 |
|  |  | GA | 0 |
|  |  | GG | 29 |
|  | rs602521720 | CC | 25 |
|  |  | GC | 1 |
|  |  | GG | 0 |
| Timahdite | rs161504783 | CC | 3 |
|  |  | TC | 15 |
|  |  | TT | 1 |
|  | rs397514116 | CC | 0 |
|  |  | GC | 0 |
|  |  | GG | 0 |
|  | rs397514117 | AA | 0 |
|  |  | CA | 0 |
|  |  | CC | 22 |
|  | rs397514269 | AA | 0 |
|  |  | GA | 0 |
|  |  | GG | 0 |
|  | rs397514272 | GG | 0 |
|  |  | TG | 0 |
|  |  | TT | 24 |
|  | rs397514273 | AA | 0 |
|  |  | GA | 0 |
|  |  | GG | 0 |
|  | rs410259751 | GG | 0 |
|  |  | TG | 23 |
|  |  | TT | 1 |
|  | rs411181557 | CC | 0 |
|  |  | CG | 0 |
|  |  | GG | 0 |
|  | rs414917134 | CC | 0 |
|  |  | GC | 0 |
|  |  | GG | 0 |
|  | rs416941267 | GG | 0 |
|  |  | TG | 0 |
|  |  | TT | 0 |
|  | rs420611298 | GG | 0 |
|  |  | TG | 0 |
|  |  | TT | 0 |
|  | rs420959261 | CC | 0 |
|  |  | TC | 0 |
|  |  | TT | 0 |
|  | rs430298704 | CC | 0 |
|  |  | TC | 0 |
|  |  | TT | 0 |
|  | rs55631463 | AA | 0 |
|  |  | GA | 0 |
|  |  | GG | 0 |
|  | rs588145625 | AA | 0 |
|  |  | GA | 0 |
|  |  | GG | 24 |
|  | rs588498137 | AA | 0 |
|  |  | GA | 0 |
|  |  | GG | 28 |
|  | rs602521720 | CC | 12 |
|  |  | GC | 7 |
|  |  | GG | 0 |
| Sardi | rs161504783 | CC | 11 |
|  |  | TC | 3 |
|  |  | TT | 0 |
|  | rs397514116 | CC | 0 |
|  |  | GC | 0 |
|  |  | GG | 0 |
|  | rs397514117 | AA | 0 |
|  |  | CA | 22 |
|  |  | CC | 0 |
|  | rs397514269 | AA | 0 |
|  |  | GA | 0 |
|  |  | GG | 0 |
|  | rs397514272 | GG | 0 |
|  |  | TG | 0 |
|  |  | TT | 0 |
|  | rs397514273 | AA | 0 |
|  |  | GA | 0 |
|  |  | GG | 27 |
|  | rs410259751 | GG | 0 |
|  |  | TG | 24 |
|  |  | TT | 1 |
|  | rs411181557 | CC | 0 |
|  |  | CG | 0 |
|  |  | GG | 0 |
|  | rs414917134 | CC | 0 |
|  |  | GC | 0 |
|  |  | GG | 0 |
|  | rs416941267 | GG | 0 |
|  |  | TG | 0 |
|  |  | TT | 0 |
|  | rs420611298 | GG | 0 |
|  |  | TG | 0 |
|  |  | TT | 0 |
|  | rs420959261 | CC | 0 |
|  |  | TC | 0 |
|  |  | TT | 0 |
|  | rs430298704 | CC | 0 |
|  |  | TC | 0 |
|  |  | TT | 0 |
|  | rs55631463 | AA | 0 |
|  |  | GA | 0 |
|  |  | GG | 0 |
|  | rs588145625 | AA | 0 |
|  |  | GA | 0 |
|  |  | GG | 28 |
|  | rs588498137 | AA | 0 |
|  |  | GA | 1 |
|  |  | GG | 26 |
|  | rs602521720 | CC | 21 |
|  |  | GC | 1 |
|  |  | GG | 0 |
| Suffolk | rs161504783 | CC | 9 |
|  |  | TC | 12 |
|  |  | TT | 5 |
|  | rs397514116 | CC | 6 |
|  |  | GC | 26 |
|  |  | GG | 4 |
|  | rs397514117 | AA | 0 |
|  |  | CA | 1 |
|  |  | CC | 25 |
|  | rs397514269 | AA | 6 |
|  |  | GA | 17 |
|  |  | GG | 3 |
|  | rs397514272 | GG | 0 |
|  |  | TG | 1 |
|  |  | TT | 25 |
|  | rs397514273 | AA | 0 |
|  |  | GA | 2 |
|  |  | GG | 24 |
|  | rs410259751 | GG | 8 |
|  |  | TG | 7 |
|  |  | TT | 10 |
|  | rs411181557 | CC | 3 |
|  |  | CG | 11 |
|  |  | GG | 12 |
|  | rs414917134 | CC | 26 |
|  |  | GC | 0 |
|  |  | GG | 0 |
|  | rs416941267 | GG | 14 |
|  |  | TG | 11 |
|  |  | TT | 1 |
|  | rs420611298 | GG | 3 |
|  |  | TG | 10 |
|  |  | TT | 13 |
|  | rs420959261 | CC | 1 |
|  |  | TC | 6 |
|  |  | TT | 19 |
|  | rs430298704 | CC | 0 |
|  |  | TC | 1 |
|  |  | TT | 25 |
|  | rs55631463 | AA | 8 |
|  |  | GA | 13 |
|  |  | GG | 4 |
|  | rs588145625 | AA | 0 |
|  |  | GA | 0 |
|  |  | GG | 26 |
|  | rs588498137 | AA | 0 |
|  |  | GA | 0 |
|  |  | GG | 26 |
|  | rs602521720 | CC | 13 |
|  |  | GC | 10 |
|  |  | GG | 3 |
| Babolna Tetra | rs161504783 | CC | 4 |
|  |  | TC | 18 |
|  |  | TT | 14 |
|  | rs397514116 | CC | 17 |
|  |  | GC | 15 |
|  |  | GG | 4 |
|  | rs397514117 | AA | 0 |
|  |  | CA | 5 |
|  |  | CC | 51 |
|  | rs397514269 | AA | 18 |
|  |  | GA | 14 |
|  |  | GG | 2 |
|  | rs397514272 | GG | 0 |
|  |  | TG | 5 |
|  |  | TT | 31 |
|  | rs397514273 | AA | 1 |
|  |  | GA | 19 |
|  |  | GG | 15 |
|  | rs410259751 | GG | 8 |
|  |  | TG | 21 |
|  |  | TT | 7 |
|  | rs411181557 | CC | 1 |
|  |  | CG | 15 |
|  |  | GG | 20 |
|  | rs414917134 | CC | 36 |
|  |  | GC | 0 |
|  |  | GG | 0 |
|  | rs416941267 | GG | 21 |
|  |  | TG | 9 |
|  |  | TT | 5 |
|  | rs420611298 | GG | 0 |
|  |  | TG | 4 |
|  |  | TT | 32 |
|  | rs420959261 | CC | 1 |
|  |  | TC | 13 |
|  |  | TT | 22 |
|  | rs430298704 | CC | 0 |
|  |  | TC | 0 |
|  |  | TT | 36 |
|  | rs55631463 | AA | 1 |
|  |  | GA | 11 |
|  |  | GG | 9 |
|  | rs588145625 | AA | 0 |
|  |  | GA | 2 |
|  |  | GG | 34 |
|  | rs588498137 | AA | 0 |
|  |  | GA | 2 |
|  |  | GG | 18 |
|  | rs602521720 | CC | 16 |
|  |  | GC | 5 |
|  |  | GG | 0 |
| Ile de France | rs161504783 | CC | 4 |
|  |  | TC | 10 |
|  |  | TT | 7 |
|  | rs397514116 | CC | 14 |
|  |  | GC | 5 |
|  |  | GG | 2 |
|  | rs397514117 | AA | 0 |
|  |  | CA | 1 |
|  |  | CC | 20 |
|  | rs397514269 | AA | 15 |
|  |  | GA | 4 |
|  |  | GG | 2 |
|  | rs397514272 | GG | 0 |
|  |  | TG | 1 |
|  |  | TT | 20 |
|  | rs397514273 | AA | 3 |
|  |  | GA | 13 |
|  |  | GG | 5 |
|  | rs410259751 | GG | 4 |
|  |  | TG | 6 |
|  |  | TT | 11 |
|  | rs411181557 | CC | 1 |
|  |  | CG | 7 |
|  |  | GG | 13 |
|  | rs414917134 | CC | 20 |
|  |  | GC | 0 |
|  |  | GG | 1 |
|  | rs416941267 | GG | 7 |
|  |  | TG | 11 |
|  |  | TT | 3 |
|  | rs420611298 | GG | 2 |
|  |  | TG | 6 |
|  |  | TT | 13 |
|  | rs420959261 | CC | 0 |
|  |  | TC | 3 |
|  |  | TT | 18 |
|  | rs430298704 | CC | 1 |
|  |  | TC | 11 |
|  |  | TT | 9 |
|  | rs55631463 | AA | 1 |
|  |  | GA | 11 |
|  |  | GG | 9 |
|  | rs588145625 | AA | 0 |
|  |  | GA | 0 |
|  |  | GG | 21 |
|  | rs588498137 | AA | 0 |
|  |  | GA | 2 |
|  |  | GG | 18 |
|  | rs602521720 | CC | 16 |
|  |  | GC | 5 |
|  |  | GG | 0 |
| Hungarian Tsigai | rs161504783 | CC | 8 |
|  |  | TC | 21 |
|  |  | TT | 4 |
|  | rs397514116 | CC | 11 |
|  |  | GC | 12 |
|  |  | GG | 7 |
|  | rs397514117 | AA | 0 |
|  |  | CA | 6 |
|  |  | CC | 26 |
|  | rs397514269 | AA | 11 |
|  |  | GA | 15 |
|  |  | GG | 6 |
|  | rs397514272 | GG | 0 |
|  |  | TG | 7 |
|  |  | TT | 26 |
|  | rs397514273 | AA | 0 |
|  |  | GA | 2 |
|  |  | GG | 26 |
|  | rs410259751 | GG | 9 |
|  |  | TG | 12 |
|  |  | TT | 12 |
|  | rs411181557 | CC | 6 |
|  |  | CG | 8 |
|  |  | GG | 18 |
|  | rs414917134 | CC | 30 |
|  |  | GC | 0 |
|  |  | GG | 3 |
|  | rs416941267 | GG | 18 |
|  |  | TG | 10 |
|  |  | TT | 4 |
|  | rs420611298 | GG | 0 |
|  |  | TG | 5 |
|  |  | TT | 28 |
|  | rs420959261 | CC | 9 |
|  |  | TC | 15 |
|  |  | TT | 6 |
|  | rs430298704 | CC | 0 |
|  |  | TC | 2 |
|  |  | TT | 29 |
|  | rs55631463 | AA | 4 |
|  |  | GA | 16 |
|  |  | GG | 13 |
|  | rs588145625 | AA | 0 |
|  |  | GA | 11 |
|  |  | GG | 22 |
|  | rs588498137 | AA | 0 |
|  |  | GA | 0 |
|  |  | GG | 33 |
|  | rs602521720 | CC | 10 |
|  |  | GC | 16 |
|  |  | GG | 7 |
| Hungarian Racka | rs161504783 | CC | 8 |
|  |  | TC | 16 |
|  |  | TT | 24 |
|  | rs397514116 | CC | 16 |
|  |  | GC | 18 |
|  |  | GG | 7 |
|  | rs397514117 | AA | 0 |
|  |  | CA | 3 |
|  |  | CC | 45 |
|  | rs397514269 | AA | 24 |
|  |  | GA | 21 |
|  |  | GG | 2 |
|  | rs397514272 | GG | 0 |
|  |  | TG | 3 |
|  |  | TT | 45 |
|  | rs397514273 | AA | 12 |
|  |  | GA | 16 |
|  |  | GG | 14 |
|  | rs410259751 | GG | 26 |
|  |  | TG | 17 |
|  |  | TT | 5 |
|  | rs411181557 | CC | 2 |
|  |  | CG | 18 |
|  |  | GG | 28 |
|  | rs414917134 | CC | 39 |
|  |  | GC | 0 |
|  |  | GG | 8 |
|  | rs416941267 | GG | 18 |
|  |  | TG | 26 |
|  |  | TT | 4 |
|  | rs420611298 | GG | 0 |
|  |  | TG | 7 |
|  |  | TT | 41 |
|  | rs420959261 | CC | 4 |
|  |  | TC | 25 |
|  |  | TT | 18 |
|  | rs430298704 | CC | 0 |
|  |  | TC | 0 |
|  |  | TT | 48 |
|  | rs55631463 | AA | 3 |
|  |  | GA | 17 |
|  |  | GG | 28 |
|  | rs588145625 | AA | 0 |
|  |  | GA | 20 |
|  |  | GG | 28 |
|  | rs588498137 | AA | 0 |
|  |  | GA | 0 |
|  |  | GG | 48 |
|  | rs602521720 | CC | 33 |
|  |  | GC | 15 |
|  |  | GG | 0 |
| Botosani Karakul | rs161504783 | CC | 2 |
|  |  | TC | 5 |
|  |  | TT | 10 |
|  | rs397514116 | CC | 3 |
|  |  | GC | 2 |
|  |  | GG | 1 |
|  | rs397514117 | AA | 0 |
|  |  | CA | 3 |
|  |  | CC | 15 |
|  | rs397514269 | AA | 5 |
|  |  | GA | 4 |
|  |  | GG | 2 |
|  | rs397514272 | GG | 0 |
|  |  | TG | 3 |
|  |  | TT | 10 |
|  | rs397514273 | AA | 0 |
|  |  | GA | 0 |
|  |  | GG | 6 |
|  | rs410259751 | GG | 7 |
|  |  | TG | 3 |
|  |  | TT | 6 |
|  | rs411181557 | CC | 2 |
|  |  | CG | 1 |
|  |  | GG | 12 |
|  | rs414917134 | CC | 14 |
|  |  | GC | 0 |
|  |  | GG | 1 |
|  | rs416941267 | GG | 11 |
|  |  | TG | 3 |
|  |  | TT | 1 |
|  | rs420611298 | GG | 2 |
|  |  | TG | 0 |
|  |  | TT | 22 |
|  | rs420959261 | CC | 0 |
|  |  | TC | 2 |
|  |  | TT | 9 |
|  | rs430298704 | CC | 0 |
|  |  | TC | 1 |
|  |  | TT | 5 |
|  | rs55631463 | AA | 2 |
|  |  | GA | 5 |
|  |  | GG | 4 |
|  | rs588145625 | AA | 0 |
|  |  | GA | 0 |
|  |  | GG | 12 |
|  | rs588498137 | AA | 2 |
|  |  | GA | 3 |
|  |  | GG | 9 |
|  | rs602521720 | CC | 13 |
|  |  | GC | 0 |
|  |  | GG | 0 |
| Romanian Racka | rs161504783 | CC | 14 |
|  |  | TC | 36 |
|  |  | TT | 11 |
|  | rs397514116 | CC | 39 |
|  |  | GC | 18 |
|  |  | GG | 2 |
|  | rs397514117 | AA | 2 |
|  |  | CA | 11 |
|  |  | CC | 49 |
|  | rs397514269 | AA | 42 |
|  |  | GA | 18 |
|  |  | GG | 1 |
|  | rs397514272 | GG | 2 |
|  |  | TG | 11 |
|  |  | TT | 49 |
|  | rs397514273 | AA | 16 |
|  |  | GA | 23 |
|  |  | GG | 22 |
|  | rs410259751 | GG | 7 |
|  |  | TG | 30 |
|  |  | TT | 23 |
|  | rs411181557 | CC | 5 |
|  |  | CG | 30 |
|  |  | GG | 26 |
|  | rs414917134 | CC | 61 |
|  |  | GC | 0 |
|  |  | GG | 0 |
|  | rs416941267 | GG | 14 |
|  |  | TG | 37 |
|  |  | TT | 11 |
|  | rs420611298 | GG | 0 |
|  |  | TG | 14 |
|  |  | TT | 46 |
|  | rs420959261 | CC | 12 |
|  |  | TC | 31 |
|  |  | TT | 19 |
|  | rs430298704 | CC | 0 |
|  |  | TC | 1 |
|  |  | TT | 60 |
|  | rs55631463 | AA | 18 |
|  |  | GA | 27 |
|  |  | GG | 17 |
|  | rs588145625 | AA | 0 |
|  |  | GA | 7 |
|  |  | GG | 55 |
|  | rs588498137 | AA | 0 |
|  |  | GA | 4 |
|  |  | GG | 58 |
|  | rs602521720 | CC | 49 |
|  |  | GC | 12 |
|  |  | GG | 1 |
| Transylvanian Merino | rs161504783 | CC | 20 |
|  |  | TC | 15 |
|  |  | TT | 7 |
|  | rs397514116 | CC | 11 |
|  |  | GC | 8 |
|  |  | GG | 6 |
|  | rs397514117 | AA | 9 |
|  |  | CA | 2 |
|  |  | CC | 42 |
|  | rs397514269 | AA | 12 |
|  |  | GA | 19 |
|  |  | GG | 7 |
|  | rs397514272 | GG | 0 |
|  |  | TG | 1 |
|  |  | TT | 41 |
|  | rs397514273 | AA | 0 |
|  |  | GA | 5 |
|  |  | GG | 29 |
|  | rs410259751 | GG | 5 |
|  |  | TG | 21 |
|  |  | TT | 17 |
|  | rs411181557 | CC | 2 |
|  |  | CG | 8 |
|  |  | GG | 33 |
|  | rs414917134 | CC | 39 |
|  |  | GC | 0 |
|  |  | GG | 1 |
|  | rs416941267 | GG | 30 |
|  |  | TG | 11 |
|  |  | TT | 3 |
|  | rs420611298 | GG | 4 |
|  |  | TG | 14 |
|  |  | TT | 22 |
|  | rs420959261 | CC | 1 |
|  |  | TC | 3 |
|  |  | TT | 38 |
|  | rs430298704 | CC | 1 |
|  |  | TC | 4 |
|  |  | TT | 37 |
|  | rs55631463 | AA | 12 |
|  |  | GA | 25 |
|  |  | GG | 8 |
|  | rs588145625 | AA | 0 |
|  |  | GA | 2 |
|  |  | GG | 43 |
|  | rs588498137 | AA | 0 |
|  |  | GA | 0 |
|  |  | GG | 45 |
|  | rs602521720 | CC | 29 |
|  |  | GC | 15 |
|  |  | GG | 0 |
| Romanian Tsigai | rs161504783 | CC | 1 |
|  |  | TC | 9 |
|  |  | TT | 5 |
|  | rs397514116 | CC | 5 |
|  |  | GC | 5 |
|  |  | GG | 1 |
|  | rs397514117 | AA | 0 |
|  |  | CA | 0 |
|  |  | CC | 17 |
|  | rs397514269 | AA | 7 |
|  |  | GA | 4 |
|  |  | GG | 2 |
|  | rs397514272 | GG | 0 |
|  |  | TG | 0 |
|  |  | TT | 16 |
|  | rs397514273 | AA | 3 |
|  |  | GA | 1 |
|  |  | GG | 7 |
|  | rs410259751 | GG | 2 |
|  |  | TG | 2 |
|  |  | TT | 15 |
|  | rs411181557 | CC | 3 |
|  |  | CG | 6 |
|  |  | GG | 7 |
|  | rs414917134 | CC | 20 |
|  |  | GC | 0 |
|  |  | GG | 0 |
|  | rs416941267 | GG | 11 |
|  |  | TG | 9 |
|  |  | TT | 7 |
|  | rs420611298 | GG | 0 |
|  |  | TG | 2 |
|  |  | TT | 16 |
|  | rs420959261 | CC | 0 |
|  |  | TC | 4 |
|  |  | TT | 12 |
|  | rs430298704 | CC | 0 |
|  |  | TC | 0 |
|  |  | TT | 13 |
|  | rs55631463 | AA | 5 |
|  |  | GA | 9 |
|  |  | GG | 1 |
|  | rs588145625 | AA | 0 |
|  |  | GA | 0 |
|  |  | GG | 16 |
|  | rs588498137 | AA | 0 |
|  |  | GA | 1 |
|  |  | GG | 17 |
|  | rs602521720 | CC | 12 |
|  |  | GC | 5 |
|  |  | GG | 0 |
| Turcana | rs161504783 | CC | 8 |
|  |  | TC | 31 |
|  |  | TT | 18 |
|  | rs397514116 | CC | 32 |
|  |  | GC | 23 |
|  |  | GG | 2 |
|  | rs397514117 | AA | 0 |
|  |  | CA | 7 |
|  |  | CC | 50 |
|  | rs397514269 | AA | 37 |
|  |  | GA | 20 |
|  |  | GG | 1 |
|  | rs397514272 | GG | 0 |
|  |  | TG | 7 |
|  |  | TT | 51 |
|  | rs397514273 | AA | 2 |
|  |  | GA | 15 |
|  |  | GG | 41 |
|  | rs410259751 | GG | 7 |
|  |  | TG | 17 |
|  |  | TT | 33 |
|  | rs411181557 | CC | 9 |
|  |  | CG | 25 |
|  |  | GG | 24 |
|  | rs414917134 | CC | 53 |
|  |  | GC | 0 |
|  |  | GG | 4 |
|  | rs416941267 | GG | 26 |
|  |  | TG | 24 |
|  |  | TT | 8 |
|  | rs420611298 | GG | 2 |
|  |  | TG | 7 |
|  |  | TT | 47 |
|  | rs420959261 | CC | 1 |
|  |  | TC | 15 |
|  |  | TT | 42 |
|  | rs430298704 | CC | 1 |
|  |  | TC | 8 |
|  |  | TT | 47 |
|  | rs55631463 | AA | 11 |
|  |  | GA | 28 |
|  |  | GG | 19 |
|  | rs588145625 | AA | 0 |
|  |  | GA | 15 |
|  |  | GG | 39 |
|  | rs588498137 | AA | 0 |
|  |  | GA | 1 |
|  |  | GG | 57 |
|  | rs602521720 | CC | 45 |
|  |  | GC | 9 |
|  |  | GG | 2 |
| Hungarian Merino | rs161504783 | CC | 16 |
|  |  | TC | 16 |
|  |  | TT | 3 |
|  | rs397514116 | CC | 11 |
|  |  | GC | 17 |
|  |  | GG | 7 |
|  | rs397514117 | AA | 0 |
|  |  | CA | 0 |
|  |  | CC | 35 |
|  | rs397514269 | AA | 11 |
|  |  | GA | 18 |
|  |  | GG | 5 |
|  | rs397514272 | GG | 0 |
|  |  | TG | 0 |
|  |  | TT | 35 |
|  | rs397514273 | AA | 2 |
|  |  | GA | 6 |
|  |  | GG | 27 |
|  | rs410259751 | \| GG \| \| --- \| | 2 |
|  |  | TG | 13 |
|  |  | TT | 20 |
|  | rs411181557 | CC | 1 |
|  |  | CG | 10 |
|  |  | GG | 24 |
|  | rs414917134 | CC | 18 |
|  |  | GC | 16 |
|  |  | GG | 0 |
|  | rs416941267 | GG | 26 |
|  |  | TG | 8 |
|  |  | TT | 1 |
|  | rs420611298 | GG | 0 |
|  |  | TG | 0 |
|  |  | TT | 35 |
|  | rs420959261 | CC | 1 |
|  |  | TC | 7 |
|  |  | TT | 27 |
|  | rs430298704 | CC | 0 |
|  |  | TC | 10 |
|  |  | TT | 25 |
|  | rs55631463 | AA | 0 |
|  |  | GA | 18 |
|  |  | GG | 16 |
|  | rs588145625 | AA | 0 |
|  |  | GA | 0 |
|  |  | GG | 35 |
|  | rs588498137 | AA | 0 |
|  |  | GA | 11 |
|  |  | GG | 24 |
|  | rs602521720 | CC | 35 |
|  |  | GC | 0 |
|  |  | GG | 0 |
| Hungarian Awassi | rs161504783 | CC | 4 |
|  |  | TC | 19 |
|  |  | TT | 17 |
|  | rs397514116 | CC | 30 |
|  |  | GC | 10 |
|  |  | GG | 0 |
|  | rs397514117 | AA | 3 |
|  |  | CA | 8 |
|  |  | CC | 29 |
|  | rs397514269 | AA | 30 |
|  |  | GA | 9 |
|  |  | GG | 0 |
|  | rs397514272 | GG | 3 |
|  |  | TG | 8 |
|  |  | TT | 29 |
|  | rs397514273 | AA | 0 |
|  |  | GA | 5 |
|  |  | GG | 35 |
|  | rs410259751 | GG | 5 |
|  |  | TG | 15 |
|  |  | TT | 20 |
|  | rs411181557 | CC | 3 |
|  |  | CG | 21 |
|  |  | GG | 16 |
|  | rs414917134 | CC | 24 |
|  |  | GC | 12 |
|  |  | GG | 1 |
|  | rs416941267 | GG | 26 |
|  |  | TG | 13 |
|  |  | TT | 1 |
|  | rs420611298 | GG | 0 |
|  |  | TG | 0 |
|  |  | TT | 40 |
|  | rs420959261 | CC | 1 |
|  |  | TC | 10 |
|  |  | TT | 28 |
|  | rs430298704 | CC | 0 |
|  |  | TC | 9 |
|  |  | TT | 30 |
|  | rs55631463 | AA | 11 |
|  |  | GA | 23 |
|  |  | GG | 6 |
|  | rs588145625 | AA | 0 |
|  |  | GA | 0 |
|  |  | GG | 40 |
|  | rs588498137 | AA | 0 |
|  |  | GA | 1 |
|  |  | GG | 39 |
|  | rs602521720 | CC | 37 |
|  |  | GC | 3 |
|  |  | GG | 0 |

Table S4. PCA Loading values

| Dim.1 Dim.2 Dim.3 Dim.4 Dim.5 |
| --- |
| a -0.0957268773 -0.22803507 -0.220966246 0.17833003 0.30942823 |
| b -0.1098545778 0.21088758 0.190115964 0.01645028 -0.49373068 |
| c 0.9018327643 0.28972654 0.006134796 0.04065301 0.04521233 |
| d -0.3097836631 0.32183409 0.636760466 -0.22322588 0.09160223 |
| e 0.8583257228 0.29329530 0.072939161 0.04691957 0.03959363 |
| f -0.0433820120 0.54614718 0.485507841 -0.03031388 0.16065120 |
| g 0.0809617873 0.15106806 -0.228951900 0.37115209 0.23628456 |
| h 0.0008438631 -0.17435902 -0.113039462 0.06780223 0.02349125 |
| i -0.0808817477 0.29922171 -0.267107244 -0.38865221 0.35386320 |
| j 0.2983367091 -0.53908887 0.148228003 -0.34371408 -0.02507619 |
| k -0.0190776933 -0.03781087 0.338941220 0.48522345 0.16514847 |
| l 0.2875181213 -0.42048722 0.235140415 -0.40438354 0.12178878 |
| m -0.1179450424 0.32688286 -0.165418159 0.16382153 0.37643513 |
| n -0.1235961207 -0.15956549 0.262046228 -0.06285451 0.53594740 |
| o -0.1283062908 0.42438807 -0.220599976 -0.11924405 -0.36220706 |
| p 0.0393344866 -0.27721318 0.242586815 0.51990040 -0.10647321 |
| q 0.0670516758 -0.16383538 0.247734410 0.20590467 -0.15792399 |

| eigenvalue percentage of variance |
| --- |
| comp 1 1.9059304 11.211355 |
| comp 2 1.6960113 9.976537 |
| comp 3 1.3251130 7.794782 |
| comp 4 1.2548843 7.381672 |
| comp 5 1.1980777 7.047516 |
| comp 6 1.1098815 6.528715 |
| comp 7 1.0280500 6.047353 |
| comp 8 1.0236191 6.021289 |
| comp 9 0.9971809 5.865770 |
| comp 10 0.9322347 5.483733 |
| comp 11 0.8981264 5.283096 |
| comp 12 0.8386303 4.933119 |
| comp 13 0.7894121 4.643600 |
| comp 14 0.6888379 4.051987 |
| comp 15 0.6020308 3.541358 |
| comp 16 0.5385607 3.168004 |
| comp 17 0.1734191 1.020112 |
| cumulative percentage of variance |
| comp 1 11.21136 |
| comp 2 21.18789 |
| comp 3 28.98267 |
| comp 4 36.36435 |
| comp 5 43.41186 |
| comp 6 49.94058 |
| comp 7 55.98793 |
| comp 8 62.00922 |
| comp 9 67.87499 |
| comp 10 73.35872 |
| comp 11 78.64182 |
| comp 12 83.57494 |
| comp 13 88.21854 |
| comp 14 92.27053 |
| comp 15 95.81188 |
| comp 16 98.97989 |
| comp 17 100.00000 |

Table S5. PCA Score values

|  | **breed** | **Characteristic** | **pca1** | **pca2** |
| --- | --- | --- | --- | --- |
| PRA 1 | PRAMENKA | COLD | -0,415607925 | 2,005842275 |
| PRA 2 | PRAMENKA | COLD | -0,228404635 | 0,13477435 |
| PRA 3 | PRAMENKA | COLD | -0,018777046 | -1,008203937 |
| PRA 4 | PRAMENKA | COLD | 0,028482393 | -0,037063831 |
| PRA 5 | PRAMENKA | COLD | -0,430075936 | 1,334005038 |
| PRA 6 | PRAMENKA | COLD | -0,073255595 | -2,109754132 |
| PRA 7 | PRAMENKA | COLD | -0,491988925 | -0,127640756 |
| PRA 8 | PRAMENKA | COLD | -0,032256603 | -1,043445607 |
| PRA 9 | PRAMENKA | COLD | 0,507646951 | -1,38845705 |
| PRA 10 | PRAMENKA | COLD | -0,354420922 | 0,129341152 |
| PRA 11 | PRAMENKA | COLD | 1,595279355 | 0,396832115 |
| PRA 12 | PRAMENKA | COLD | -1,213576232 | 1,367590945 |
| PRA 13 | PRAMENKA | COLD | -0,233806037 | -0,824072246 |
| PRA 14 | PRAMENKA | COLD | -0,278864954 | -0,083346219 |
| PRA 15 | PRAMENKA | COLD | -0,601692592 | 0,080856033 |
| PRA 16 | PRAMENKA | COLD | -0,226718705 | -1,198943552 |
| PRA 17 | PRAMENKA | COLD | -0,619191851 | -0,515526486 |
| PRA 18 | PRAMENKA | COLD | -0,821246135 | 0,947141067 |
| PRA 19 | PRAMENKA | COLD | -0,548134682 | -1,407469958 |
| PRA 20 | PRAMENKA | COLD | -0,064086413 | 0,01332179 |
| PRA 21 | PRAMENKA | COLD | 0,243888132 | -2,137218047 |
| PRA 22 | PRAMENKA | COLD | -0,579718943 | 0,55660855 |
| PRA 23 | PRAMENKA | COLD | 0,41370938 | -0,465407141 |
| PRA 24 | PRAMENKA | COLD | 1,725294481 | 0,193018388 |
| PRA 25 | PRAMENKA | COLD | -0,169279349 | 0,427327971 |
| PRA 26 | PRAMENKA | COLD | -0,382104038 | -0,764818766 |
| PRA 27 | PRAMENKA | COLD | -0,215381631 | -0,052944425 |
| PRA 28 | PRAMENKA | COLD | -1,034244587 | 0,485630093 |
| PRA 29 | PRAMENKA | COLD | 0,15113485 | -2,594946563 |
| PRA 30 | PRAMENKA | COLD | -0,252662316 | -0,54811174 |
| PRA 31 | PRAMENKA | COLD | -0,762363927 | 0,205980361 |
| PRA 32 | PRAMENKA | COLD | -0,350495321 | -0,559932055 |
| PRA 33 | PRAMENKA | COLD | -0,574771374 | 0,522227845 |
| PRA 34 | PRAMENKA | COLD | 1,494132034 | -0,193991868 |
| PRA 35 | PRAMENKA | COLD | -0,696707751 | 0,956165286 |
| PRA 36 | PRAMENKA | COLD | -0,101747216 | -1,225831541 |
| PRA 37 | PRAMENKA | COLD | -0,52178537 | -0,118616673 |
| BG 1 | BENI GUIL | HOT | -0,028539759 | -0,068678003 |
| BG 2 | BENI GUIL | HOT | -0,063456557 | 0,05375226 |
| BG 3 | BENI GUIL | HOT | -0,063456557 | 0,05375226 |
| BG 4 | BENI GUIL | HOT | -0,044356357 | 0,004278476 |
| BG 5 | BENI GUIL | HOT | -0,063456557 | 0,05375226 |
| BG 6 | BENI GUIL | HOT | -0,063456557 | 0,05375226 |
| BG 7 | BENI GUIL | HOT | -0,063456557 | 0,05375226 |
| BG 8 | BENI GUIL | HOT | -0,109925377 | -0,038164218 |
| BG 9 | BENI GUIL | HOT | 0,034259665 | 0,089907166 |
| BG 10 | BENI GUIL | HOT | -0,000657133 | 0,212337429 |
| BG 11 | BENI GUIL | HOT | -0,063456557 | 0,05375226 |
| BG 12 | BENI GUIL | HOT | -0,063456557 | 0,05375226 |
| BG 13 | BENI GUIL | HOT | -0,044356357 | 0,004278476 |
| BG 14 | BENI GUIL | HOT | -0,063456557 | 0,05375226 |
| BG 15 | BENI GUIL | HOT | -0,063456557 | 0,05375226 |
| BG 16 | BENI GUIL | HOT | -0,063456557 | 0,05375226 |
| BG 17 | BENI GUIL | HOT | -0,047125953 | 0,120420952 |
| BG 18 | BENI GUIL | HOT | -0,237674156 | -0,290854465 |
| BG 19 | BENI GUIL | HOT | -0,028539759 | -0,068678003 |
| BG 20 | BENI GUIL | HOT | -0,019879642 | 0,189225099 |
| BG 21 | BENI GUIL | HOT | -0,063456557 | 0,05375226 |
| BG 22 | BENI GUIL | HOT | -0,028539759 | -0,068678003 |
| BG 23 | BENI GUIL | HOT | -0,063456557 | 0,05375226 |
| BG 24 | BENI GUIL | HOT | 0,116634913 | 0,508530894 |
| BG 25 | BENI GUIL | HOT | -0,000657133 | 0,212337429 |
| BG 26 | BENI GUIL | HOT | -0,063456557 | 0,05375226 |
| BG 27 | BENI GUIL | HOT | -0,028539759 | -0,068678003 |
| BG 28 | BENI GUIL | HOT | -0,237674156 | -0,290854465 |
| BG 29 | BENI GUIL | HOT | -0,000657133 | 0,212337429 |
| DM 1 | D'MAN | HOT | -0,109925377 | -0,038164218 |
| DM 2 | D'MAN | HOT | -0,047125953 | 0,120420952 |
| DM 3 | D'MAN | HOT | -0,109925377 | -0,038164218 |
| DM 4 | D'MAN | HOT | -0,012209155 | -0,002009312 |
| DM 5 | D'MAN | HOT | -0,075008579 | -0,160594481 |
| DM 6 | D'MAN | HOT | -0,075008579 | -0,160594481 |
| DM 7 | D'MAN | HOT | -0,075008579 | -0,160594481 |
| DM 8 | D'MAN | HOT | -0,075008579 | -0,160594481 |
| DM 9 | D'MAN | HOT | -0,047125953 | 0,120420952 |
| DM 10 | D'MAN | HOT | -0,028025753 | 0,070947167 |
| DM 11 | D'MAN | HOT | -0,109925377 | -0,038164218 |
| DM 12 | D'MAN | HOT | -0,075008579 | -0,160594481 |
| DM 13 | D'MAN | HOT | -0,075008579 | -0,160594481 |
| DM 14 | D'MAN | HOT | -0,075008579 | -0,160594481 |
| DM 15 | D'MAN | HOT | -0,075008579 | -0,160594481 |
| DM 16 | D'MAN | HOT | -0,109925377 | -0,038164218 |
| DM 17 | D'MAN | HOT | -0,028025753 | 0,070947167 |
| DM 18 | D'MAN | HOT | -0,019879642 | 0,189225099 |
| DM 19 | D'MAN | HOT | 0,006891045 | -0,051483096 |
| DM 20 | D'MAN | HOT | -0,109925377 | -0,038164218 |
| DM 21 | D'MAN | HOT | -0,109925377 | -0,038164218 |
| DM 22 | D'MAN | HOT | -0,109925377 | -0,038164218 |
| DM 23 | D'MAN | HOT | -0,109925377 | -0,038164218 |
| DM 24 | D'MAN | HOT | -0,019879642 | 0,189225099 |
| DM 25 | D'MAN | HOT | 0,071083065 | -0,185766803 |
| DM 26 | D'MAN | HOT | -0,019879642 | 0,189225099 |
| DM 27 | D'MAN | HOT | -0,075008579 | -0,160594481 |
| DM 28 | D'MAN | HOT | -0,109925377 | -0,038164218 |
| DM 29 | D'MAN | HOT | -0,109925377 | -0,038164218 |
| TIM 1 | TIMAHDITE | HOT | 0,116634913 | 0,508530894 |
| TIM 2 | TIMAHDITE | HOT | -0,115277138 | 0,170817888 |
| TIM 3 | TIMAHDITE | HOT | -0,405824065 | -0,065330361 |
| TIM 4 | TIMAHDITE | HOT | -0,308107843 | -0,029175455 |
| TIM 5 | TIMAHDITE | HOT | -0,452292885 | -0,157246838 |
| TIM 6 | TIMAHDITE | HOT | -0,225732595 | 0,389448273 |
| TIM 7 | TIMAHDITE | HOT | -0,044356357 | 0,004278476 |
| TIM 8 | TIMAHDITE | HOT | -0,059867544 | -0,293955036 |
| TIM 9 | TIMAHDITE | HOT | -0,000657133 | 0,212337429 |
| TIM 10 | TIMAHDITE | HOT | -0,398275887 | -0,329150886 |
| TIM 11 | TIMAHDITE | HOT | -0,224815623 | -0,212932945 |
| TIM 12 | TIMAHDITE | HOT | -0,224815623 | -0,212932945 |
| TIM 13 | TIMAHDITE | HOT | -0,287615047 | -0,371518115 |
| TIM 14 | TIMAHDITE | HOT | -0,225732595 | 0,389448273 |
| TIM 15 | TIMAHDITE | HOT | -0,386723865 | -0,114804145 |
| TIM 16 | TIMAHDITE | HOT | -0,386723865 | -0,114804145 |
| TIM 17 | TIMAHDITE | HOT | -0,287615047 | -0,371518115 |
| TIM 18 | TIMAHDITE | HOT | -0,370907267 | -0,187760624 |
| TIM 19 | TIMAHDITE | HOT | -0,343024641 | 0,093254809 |
| TIM 20 | TIMAHDITE | HOT | -0,433192685 | -0,206720623 |
| TIM 21 | TIMAHDITE | HOT | -0,343024641 | 0,093254809 |
| TIM 22 | TIMAHDITE | HOT | -0,386723865 | -0,114804145 |
| TIM 23 | TIMAHDITE | HOT | -0,287615047 | -0,371518115 |
| TIM 24 | TIMAHDITE | HOT | -0,370393261 | -0,048135453 |
| TIM 25 | TIMAHDITE | HOT | -0,386723865 | -0,114804145 |
| TIM 26 | TIMAHDITE | HOT | -0,405824065 | -0,065330361 |
| TIM 27 | TIMAHDITE | HOT | 0,018443068 | 0,162863645 |
| TIM 28 | TIMAHDITE | HOT | -0,206206913 | -0,352710743 |
| SAR 1 | SARDI | HOT | 0,243373253 | 0,574269171 |
| SAR 2 | SARDI | HOT | 0,224273053 | 0,623742955 |
| SAR 3 | SARDI | HOT | 0,224273053 | 0,623742955 |
| SAR 4 | SARDI | HOT | 0,224273053 | 0,623742955 |
| SAR 5 | SARDI | HOT | 0,341565099 | 0,91993642 |
| SAR 6 | SARDI | HOT | 0,341565099 | 0,91993642 |
| SAR 7 | SARDI | HOT | 0,341565099 | 0,91993642 |
| SAR 8 | SARDI | HOT | 0,278290051 | 0,451838908 |
| SAR 9 | SARDI | HOT | 0,224273053 | 0,623742955 |
| SAR 10 | SARDI | HOT | 0,341565099 | 0,91993642 |
| SAR 11 | SARDI | HOT | 0,341565099 | 0,91993642 |
| SAR 12 | SARDI | HOT | 0,224273053 | 0,623742955 |
| SAR 13 | SARDI | HOT | 0,341565099 | 0,91993642 |
| SAR 14 | SARDI | HOT | 0,224273053 | 0,623742955 |
| SAR 15 | SARDI | HOT | -0,012910719 | 0,540817491 |
| SAR 16 | SARDI | HOT | -0,025119874 | 0,538808179 |
| SAR 17 | SARDI | HOT | 0,045703352 | 0,743085165 |
| SAR 18 | SARDI | HOT | 0,334674053 | 0,971419516 |
| SAR 19 | SARDI | HOT | 0,177804232 | 0,531826478 |
| SAR 20 | SARDI | HOT | -0,025119874 | 0,538808179 |
| SAR 21 | SARDI | HOT | -0,006019673 | 0,489334394 |
| SAR 22 | SARDI | HOT | 0,341565099 | 0,91993642 |
| SAR 23 | SARDI | HOT | -0,006019673 | 0,489334394 |
| SAR 24 | SARDI | HOT | -0,087919298 | 0,38022301 |
| SAR 25 | SARDI | HOT | 0,202266973 | 0,205355728 |
| SAR 26 | SARDI | HOT | 0,341565099 | 0,91993642 |
| SAR 27 | SARDI | HOT | 0,459774117 | 0,613748665 |
| SAR 28 | SARDI | HOT | 0,019122995 | 1,182799756 |
| SAR 29 | SARDI | HOT | 0,16148217 | 0,003061571 |
| SUF 1 | SUFFOLK | COLD | -0,247754652 | -0,915656329 |
| SUF 2 | SUFFOLK | COLD | -0,353324314 | 0,593889988 |
| SUF 3 | SUFFOLK | COLD | -0,195400822 | 0,270717103 |
| SUF 4 | SUFFOLK | COLD | -0,343858655 | 0,796856486 |
| SUF 5 | SUFFOLK | COLD | -0,767242474 | 0,885162373 |
| SUF 6 | SUFFOLK | COLD | -0,956443603 | 0,599680073 |
| SUF 7 | SUFFOLK | COLD | -0,987791088 | 1,719315732 |
| SUF 8 | SUFFOLK | COLD | -0,060723385 | -0,23720826 |
| SUF 9 | SUFFOLK | COLD | -0,551473667 | 0,245795614 |
| SUF 10 | SUFFOLK | COLD | -0,774428241 | 0,140423479 |
| SUF 11 | SUFFOLK | COLD | -0,796579079 | 1,377200401 |
| SUF 12 | SUFFOLK | COLD | -0,058169553 | 0,415354994 |
| SUF 13 | SUFFOLK | COLD | -0,563393766 | -0,403264887 |
| SUF 14 | SUFFOLK | COLD | -0,346204989 | 0,709649933 |
| SUF 15 | SUFFOLK | COLD | 0,238290901 | -0,861030167 |
| SUF 16 | SUFFOLK | COLD | -0,86958207 | 1,413127978 |
| SUF 17 | SUFFOLK | COLD | -0,724124565 | -0,832408308 |
| SUF 18 | SUFFOLK | COLD | 0,048611465 | -0,748561566 |
| SUF 19 | SUFFOLK | COLD | -1,070954753 | 1,112786868 |
| SUF 20 | SUFFOLK | COLD | -0,800070829 | 2,329159094 |
| SUF 21 | SUFFOLK | COLD | 3,568658527 | 2,244676972 |
| SUF 22 | SUFFOLK | COLD | -0,551641748 | 0,599496038 |
| SUF 23 | SUFFOLK | COLD | -0,240594815 | -0,691722505 |
| SUF 24 | SUFFOLK | COLD | -0,854598539 | 1,354003553 |
| SUF 25 | SUFFOLK | COLD | -1,05115441 | 1,282710407 |
| SUF 26 | SUFFOLK | COLD | -0,09480667 | -1,300933908 |
| BTET 1 | BABOLNA TETRA | COLD | 0,300557605 | -1,452473773 |
| BTET 2 | BABOLNA TETRA | COLD | -1,102977495 | 1,492611757 |
| BTET 3 | BABOLNA TETRA | COLD | 4,766131019 | 0,025550834 |
| BTET 4 | BABOLNA TETRA | COLD | 0,168614473 | -0,342035309 |
| BTET 5 | BABOLNA TETRA | COLD | 0,416703694 | -1,62649576 |
| BTET 6 | BABOLNA TETRA | COLD | 0,382308044 | -1,36964942 |
| BTET 7 | BABOLNA TETRA | COLD | 0,415498824 | -3,709225987 |
| BTET 8 | BABOLNA TETRA | COLD | -0,799338551 | 0,729118061 |
| BTET 9 | BABOLNA TETRA | COLD | -0,012690741 | -1,031133909 |
| BTET 10 | BABOLNA TETRA | COLD | -0,569752634 | -0,429824069 |
| BTET 11 | BABOLNA TETRA | COLD | -0,552400872 | 0,121093133 |
| BTET 12 | BABOLNA TETRA | COLD | -0,407641503 | -0,613978939 |
| BTET 13 | BABOLNA TETRA | COLD | -0,518843492 | -0,557861542 |
| BTET 14 | BABOLNA TETRA | COLD | -1,15324674 | 1,16953147 |
| BTET 15 | BABOLNA TETRA | COLD | -0,368351077 | -1,094508316 |
| BTET 16 | BABOLNA TETRA | COLD | 0,651232083 | -1,88478607 |
| BTET 17 | BABOLNA TETRA | COLD | -1,034690009 | 0,899224919 |
| BTET 18 | BABOLNA TETRA | COLD | -0,861998929 | 0,488380236 |
| BTET 19 | BABOLNA TETRA | COLD | 3,490659387 | 1,903077275 |
| BTET 20 | BABOLNA TETRA | COLD | -0,955849895 | 1,367581312 |
| BTET 21 | BABOLNA TETRA | COLD | -0,736925892 | 0,393368374 |
| BTET 22 | BABOLNA TETRA | COLD | 4,105450777 | 0,736797182 |
| BTET 23 | BABOLNA TETRA | COLD | 0,516151078 | -1,873966006 |
| BTET 24 | BABOLNA TETRA | COLD | -0,874476173 | 1,821537072 |
| BTET 25 | BABOLNA TETRA | COLD | -1,095798083 | 0,098244905 |
| BTET 26 | BABOLNA TETRA | COLD | -1,055861424 | 0,958128844 |
| BTET 27 | BABOLNA TETRA | COLD | -0,234904775 | 0,054988849 |
| BTET 28 | BABOLNA TETRA | COLD | -0,077757395 | -1,206041709 |
| BTET 29 | BABOLNA TETRA | COLD | 3,17286869 | 2,352347812 |
| BTET 30 | BABOLNA TETRA | COLD | -0,839868538 | -0,591170593 |
| BTET 31 | BABOLNA TETRA | COLD | 0,084081815 | -0,059407405 |
| BTET 32 | BABOLNA TETRA | COLD | 3,752777399 | 2,117670923 |
| BTET 33 | BABOLNA TETRA | COLD | 0,140755453 | 0,132744515 |
| BTET 34 | BABOLNA TETRA | COLD | -0,539495783 | 0,949572397 |
| BTET 35 | BABOLNA TETRA | COLD | -0,127044201 | 0,370095838 |
| BTET 36 | BABOLNA TETRA | COLD | -1,100896694 | 1,196338225 |
| IDF 1 | ILE DE FRANCE | COLD | 0,17923387 | -1,887826504 |
| IDF 2 | ILE DE FRANCE | COLD | 0,061391644 | -2,300505554 |
| IDF 3 | ILE DE FRANCE | COLD | 0,292777816 | -1,283594607 |
| IDF 4 | ILE DE FRANCE | COLD | 0,247767351 | -1,749193305 |
| IDF 5 | ILE DE FRANCE | COLD | 0,015863742 | -1,157232993 |
| IDF 6 | ILE DE FRANCE | COLD | -0,481146334 | 0,296221433 |
| IDF 7 | ILE DE FRANCE | COLD | -1,339077546 | 0,387038644 |
| IDF 8 | ILE DE FRANCE | COLD | -0,02349922 | -1,092647802 |
| IDF 9 | ILE DE FRANCE | COLD | 0,103838816 | -1,333306537 |
| IDF 10 | ILE DE FRANCE | COLD | -1,19680114 | 1,083379789 |
| IDF 11 | ILE DE FRANCE | COLD | 3,623657385 | 2,541948634 |
| IDF 12 | ILE DE FRANCE | COLD | 0,064815445 | -0,788640955 |
| IDF 13 | ILE DE FRANCE | COLD | 0,220934294 | -0,649972842 |
| IDF 14 | ILE DE FRANCE | COLD | -0,477091998 | -0,235551316 |
| IDF 15 | ILE DE FRANCE | COLD | -0,563806656 | 0,462845212 |
| IDF 16 | ILE DE FRANCE | COLD | -0,672347668 | 0,176709342 |
| IDF 17 | ILE DE FRANCE | COLD | 0,104579252 | -1,495487068 |
| IDF 18 | ILE DE FRANCE | COLD | -1,042695133 | 0,556202274 |
| IDF 19 | ILE DE FRANCE | COLD | -0,490639415 | 0,306281369 |
| IDF 20 | ILE DE FRANCE | COLD | 0,000920221 | -0,875068809 |
| IDF 21 | ILE DE FRANCE | COLD | 0,035324167 | -2,017340775 |
| HUTSI 1 | HU TSIGAI | COLD | 0,014100565 | -0,758903932 |
| HUTSI 2 | HU TSIGAI | COLD | -0,149838365 | -1,825817234 |
| HUTSI 3 | HU TSIGAI | COLD | -0,217071297 | -0,517236185 |
| HUTSI 4 | HU TSIGAI | COLD | -0,645021568 | 0,494846761 |
| HUTSI 5 | HU TSIGAI | COLD | -0,449967869 | 0,369679543 |
| HUTSI 6 | HU TSIGAI | COLD | -0,515200288 | -1,084321216 |
| HUTSI 7 | HU TSIGAI | COLD | -0,828095855 | 0,43573812 |
| HUTSI 8 | HU TSIGAI | COLD | -0,317354116 | -1,73789489 |
| HUTSI 9 | HU TSIGAI | COLD | 0,184515159 | -2,218671358 |
| HUTSI 10 | HU TSIGAI | COLD | 4,382818935 | -0,482976244 |
| HUTSI 11 | HU TSIGAI | COLD | -0,22307114 | -0,176310893 |
| HUTSI 12 | HU TSIGAI | COLD | -0,06893651 | -0,551411695 |
| HUTSI 13 | HU TSIGAI | COLD | -0,713218386 | 1,279517368 |
| HUTSI 14 | HU TSIGAI | COLD | 4,146673707 | 1,612657223 |
| HUTSI 15 | HU TSIGAI | COLD | 4,420291482 | -0,439113137 |
| HUTSI 16 | HU TSIGAI | COLD | 3,611876986 | 1,32076852 |
| HUTSI 17 | HU TSIGAI | COLD | -1,099530545 | 1,684816856 |
| HUTSI 18 | HU TSIGAI | COLD | -1,624318396 | 1,093358567 |
| HUTSI 19 | HU TSIGAI | COLD | -1,609853837 | 1,786219789 |
| HUTSI 20 | HU TSIGAI | COLD | 0,135076707 | -1,203644654 |
| HUTSI 21 | HU TSIGAI | COLD | -1,625058831 | 1,255539098 |
| HUTSI 22 | HU TSIGAI | COLD | 4,226562382 | -0,152153562 |
| HUTSI 23 | HU TSIGAI | COLD | -1,229492402 | 1,634872424 |
| HUTSI 24 | HU TSIGAI | COLD | -0,527070294 | -0,065727794 |
| HUTSI 25 | HU TSIGAI | COLD | -0,148254023 | -0,431266836 |
| HUTSI 26 | HU TSIGAI | COLD | 0,089804352 | -1,535641024 |
| HUTSI 27 | HU TSIGAI | COLD | -0,63232231 | 0,341695912 |
| HUTSI 28 | HU TSIGAI | COLD | -0,709669823 | -0,63518111 |
| HUTSI 29 | HU TSIGAI | COLD | -1,586007913 | 2,198943572 |
| HUTSI 30 | HU TSIGAI | COLD | 4,801689839 | 0,675504591 |
| HUTSI 31 | HU TSIGAI | COLD | -0,372876952 | -1,907265723 |
| HUTSI 32 | HU TSIGAI | COLD | -0,855189141 | 1,386124151 |
| HUTSI 33 | HU TSIGAI | COLD | 1,404708765 | 0,747402334 |
| HURAC 1 | HU RACKA | COLD | 0,185354234 | -1,909187228 |
| HURAC 2 | HU RACKA | COLD | 0,228676964 | -1,916489468 |
| HURAC 3 | HU RACKA | COLD | 0,478645241 | -3,917141546 |
| HURAC 4 | HU RACKA | COLD | 0,150203641 | -3,103712665 |
| HURAC 5 | HU RACKA | COLD | 0,069194042 | 0,094016486 |
| HURAC 6 | HU RACKA | COLD | -1,147372869 | 1,279703379 |
| HURAC 7 | HU RACKA | COLD | -1,235937733 | 0,727952999 |
| HURAC 8 | HU RACKA | COLD | 0,699544689 | -4,149314202 |
| HURAC 9 | HU RACKA | COLD | 0,915643683 | -3,130626098 |
| HURAC 10 | HU RACKA | COLD | -0,28390498 | -1,028771813 |
| HURAC 11 | HU RACKA | COLD | -0,878354023 | 0,507806273 |
| HURAC 12 | HU RACKA | COLD | 0,372842823 | -1,392797787 |
| HURAC 13 | HU RACKA | COLD | -0,035525545 | -0,81527037 |
| HURAC 14 | HU RACKA | COLD | 0,686088248 | -2,79057066 |
| HURAC 15 | HU RACKA | COLD | -0,744199621 | -0,599177549 |
| HURAC 16 | HU RACKA | COLD | -0,221903864 | -0,230813373 |
| HURAC 17 | HU RACKA | COLD | -0,194526336 | -2,621934999 |
| HURAC 18 | HU RACKA | COLD | -0,176504562 | -1,808706373 |
| HURAC 19 | HU RACKA | COLD | -0,363938357 | -2,076709997 |
| HURAC 20 | HU RACKA | COLD | -0,377000511 | -1,449267162 |
| HURAC 21 | HU RACKA | COLD | -0,04922732 | -2,458485584 |
| HURAC 22 | HU RACKA | COLD | -0,574811278 | 0,18906396 |
| HURAC 23 | HU RACKA | COLD | 0,416988848 | -2,183981684 |
| HURAC 24 | HU RACKA | COLD | -1,050978723 | -0,959051675 |
| HURAC 25 | HU RACKA | COLD | -0,374677633 | -0,034169861 |
| HURAC 26 | HU RACKA | COLD | 0,487916674 | -3,064341089 |
| HURAC 27 | HU RACKA | COLD | -0,67158014 | 0,753368898 |
| HURAC 28 | HU RACKA | COLD | 0,021456993 | -1,585226161 |
| HURAC 29 | HU RACKA | COLD | 0,125322377 | -2,283407249 |
| HURAC 30 | HU RACKA | COLD | -1,063201005 | 1,396920787 |
| HURAC 31 | HU RACKA | COLD | -1,237418604 | 1,052314062 |
| HURAC 32 | HU RACKA | COLD | 0,352893733 | -2,001122698 |
| HURAC 33 | HU RACKA | COLD | -0,26193426 | -0,995101205 |
| HURAC 34 | HU RACKA | COLD | -0,335301882 | -0,980830142 |
| HURAC 35 | HU RACKA | COLD | 0,917027938 | -3,09778358 |
| HURAC 36 | HU RACKA | COLD | 0,535380706 | -3,329916659 |
| HURAC 37 | HU RACKA | COLD | -0,307575988 | -1,522589736 |
| HURAC 38 | HU RACKA | COLD | 0,475533614 | -1,905031733 |
| HURAC 39 | HU RACKA | COLD | 0,221014184 | -1,308382839 |
| HURAC 40 | HU RACKA | COLD | 4,225314671 | -0,277106593 |
| HURAC 41 | HU RACKA | COLD | -0,514306155 | -0,879025973 |
| HURAC 42 | HU RACKA | COLD | -0,410254422 | -2,537123597 |
| HURAC 43 | HU RACKA | COLD | 0,929309651 | -3,87507088 |
| HURAC 44 | HU RACKA | COLD | 0,042299286 | -2,55545733 |
| HURAC 45 | HU RACKA | COLD | 0,198360381 | -1,424695469 |
| HURAC 46 | HU RACKA | COLD | 3,558546639 | 0,515615897 |
| HURAC 47 | HU RACKA | COLD | 4,685143357 | -0,677725162 |
| HURAC 48 | HU RACKA | COLD | 0,607701913 | -3,218543808 |
| KAR 1 | BOTOSANI KARAKUL | HOT EU | -0,670812276 | 0,15636841 |
| KAR 2 | BOTOSANI KARAKUL | HOT EU | -0,477182888 | -0,510151517 |
| KAR 3 | BOTOSANI KARAKUL | HOT EU | -0,219137713 | -0,125435062 |
| KAR 4 | BOTOSANI KARAKUL | HOT EU | -0,624385476 | 1,883791992 |
| KAR 5 | BOTOSANI KARAKUL | HOT EU | -0,128451554 | 0,406342563 |
| KAR 6 | BOTOSANI KARAKUL | HOT EU | 0,001104103 | 0,138238393 |
| KAR 7 | BOTOSANI KARAKUL | HOT EU | -0,099398364 | -0,045647706 |
| KAR 8 | BOTOSANI KARAKUL | HOT EU | 1,367108551 | 0,742860406 |
| KAR 9 | BOTOSANI KARAKUL | HOT EU | -0,033198054 | 0,126041377 |
| KAR 10 | BOTOSANI KARAKUL | HOT EU | -0,459009265 | 0,508253766 |
| KAR 11 | BOTOSANI KARAKUL | HOT EU | -1,409548332 | 2,674303579 |
| KAR 12 | BOTOSANI KARAKUL | HOT EU | 2,17142029 | 1,408994217 |
| KAR 13 | BOTOSANI KARAKUL | HOT EU | -1,636926333 | 2,930619289 |
| KAR 14 | BOTOSANI KARAKUL | HOT EU | 0,02909085 | 0,04486201 |
| KAR 15 | BOTOSANI KARAKUL | HOT EU | -0,136054357 | 0,150287422 |
| KAR 16 | BOTOSANI KARAKUL | HOT EU | 3,874913979 | 1,672080222 |
| KAR 17 | BOTOSANI KARAKUL | HOT EU | -0,115863349 | 0,844204636 |
| KAR 18 | BOTOSANI KARAKUL | HOT EU | -0,638347343 | 0,305416584 |
| KAR 19 | BOTOSANI KARAKUL | HOT EU | -0,098957525 | 0,149200765 |
| KAR 20 | BOTOSANI KARAKUL | HOT EU | -0,526609598 | 0,631489254 |
| KAR 21 | BOTOSANI KARAKUL | HOT EU | -0,646009027 | 0,682785561 |
| KAR 22 | BOTOSANI KARAKUL | HOT EU | -0,665723243 | 0,151408427 |
| KAR 23 | BOTOSANI KARAKUL | HOT EU | -0,779066025 | 0,27126743 |
| KAR 24 | BOTOSANI KARAKUL | HOT EU | -0,874840714 | 0,182846628 |
| KAR 25 | BOTOSANI KARAKUL | HOT EU | 4,666608833 | 0,686324656 |
| RORAC 1 | RO RACKA | COLD | -0,347104952 | -0,535626334 |
| RORAC 2 | RO RACKA | COLD | -0,644448046 | -1,435204616 |
| RORAC 3 | RO RACKA | COLD | 4,407418158 | -0,117237859 |
| RORAC 4 | RO RACKA | COLD | 4,493700692 | 0,626482169 |
| RORAC 5 | RO RACKA | COLD | -0,067491296 | -0,741621522 |
| RORAC 6 | RO RACKA | COLD | 1,008513109 | -3,369050449 |
| RORAC 7 | RO RACKA | COLD | 0,617154135 | -2,85910089 |
| RORAC 8 | RO RACKA | COLD | -0,380551505 | -0,14434177 |
| RORAC 9 | RO RACKA | COLD | -0,056437293 | -1,793725013 |
| RORAC 10 | RO RACKA | COLD | -1,245476558 | 0,697461523 |
| RORAC 11 | RO RACKA | COLD | 0,534640566 | -1,431059708 |
| RORAC 12 | RO RACKA | COLD | -0,285061064 | -0,006568354 |
| RORAC 13 | RO RACKA | COLD | 0,971055617 | -2,738568709 |
| RORAC 14 | RO RACKA | COLD | 3,76332053 | 1,685721053 |
| RORAC 15 | RO RACKA | COLD | 0,66193521 | -2,218248946 |
| RORAC 16 | RO RACKA | COLD | 0,142969908 | -1,629350156 |
| RORAC 17 | RO RACKA | COLD | 4,72405749 | -0,384961909 |
| RORAC 18 | RO RACKA | COLD | -0,280042369 | -0,936935346 |
| RORAC 19 | RO RACKA | COLD | 0,385854671 | -2,851287854 |
| RORAC 20 | RO RACKA | COLD | 0,316017433 | -1,650732184 |
| RORAC 21 | RO RACKA | COLD | 5,013324135 | -1,008090406 |
| RORAC 22 | RO RACKA | COLD | 0,407592894 | -0,726095989 |
| RORAC 23 | RO RACKA | COLD | 0,381464603 | -1,855846591 |
| RORAC 24 | RO RACKA | COLD | -0,461498373 | 0,550102216 |
| RORAC 25 | RO RACKA | COLD | -0,227612407 | -1,07785492 |
| RORAC 26 | RO RACKA | COLD | 4,857657625 | -0,071420911 |
| RORAC 27 | RO RACKA | COLD | 4,370061824 | 1,344674427 |
| RORAC 28 | RO RACKA | COLD | -0,485943016 | -0,573145094 |
| RORAC 29 | RO RACKA | COLD | -1,099743999 | 0,487556007 |
| RORAC 30 | RO RACKA | COLD | -0,183520434 | -1,19423783 |
| RORAC 31 | RO RACKA | COLD | 0,649901819 | -2,521430764 |
| RORAC 32 | RO RACKA | COLD | -0,065862158 | -0,598971529 |
| RORAC 33 | RO RACKA | COLD | 3,651853504 | 2,235983227 |
| RORAC 34 | RO RACKA | COLD | -0,327134593 | -0,417081098 |
| RORAC 35 | RO RACKA | COLD | 0,334592751 | -1,61706202 |
| RORAC 36 | RO RACKA | COLD | -0,414110331 | 0,043005901 |
| RORAC 37 | RO RACKA | COLD | -0,053448771 | -1,432048713 |
| RORAC 38 | RO RACKA | COLD | -0,207425348 | -0,928248158 |
| RORAC 39 | RO RACKA | COLD | 0,501119917 | -3,464484257 |
| RORAC 40 | RO RACKA | COLD | -0,304985316 | -0,308100243 |
| RORAC 41 | RO RACKA | COLD | -0,594348528 | 0,518309038 |
| RORAC 42 | RO RACKA | COLD | 0,534655275 | -3,564247996 |
| RORAC 43 | RO RACKA | COLD | -0,156422952 | -0,574505097 |
| RORAC 44 | RO RACKA | COLD | -0,55484227 | -0,316473473 |
| RORAC 45 | RO RACKA | COLD | -5,592730658 | -3,023460907 |
| RORAC 46 | RO RACKA | COLD | -5,579203192 | -3,462032349 |
| RORAC 47 | RO RACKA | COLD | 4,28675051 | -0,26408696 |
| RORAC 48 | RO RACKA | COLD | -0,035613889 | -1,236198017 |
| RORAC 49 | RO RACKA | COLD | -0,014874825 | -1,27581454 |
| RORAC 50 | RO RACKA | COLD | -0,459481722 | 0,389893385 |
| RORAC 51 | RO RACKA | COLD | -0,302870122 | -1,035289405 |
| RORAC 52 | RO RACKA | COLD | -0,432084855 | 1,211411827 |
| RORAC 53 | RO RACKA | COLD | -0,269420674 | -0,286801061 |
| RORAC 54 | RO RACKA | COLD | 3,859835649 | 1,025883076 |
| RORAC 55 | RO RACKA | COLD | -0,591263576 | 0,355835093 |
| RORAC 56 | RO RACKA | COLD | -0,345430635 | 1,138136897 |
| RORAC 57 | RO RACKA | COLD | 3,547367608 | 0,993971242 |
| RORAC 58 | RO RACKA | COLD | -0,719767705 | 0,657841307 |
| RORAC 59 | RO RACKA | COLD | 0,441455665 | -2,879346552 |
| RORAC 60 | RO RACKA | COLD | -0,273613897 | -0,346292976 |
| RORAC 61 | RO RACKA | COLD | -0,291937606 | -1,544005526 |
| RORAC 62 | RO RACKA | COLD | -0,45420155 | 0,062770588 |
| TRANSMER 1 | PENSYLVANIAN MERINO | HOT EU | -0,415007037 | 0,691339851 |
| TRANSMER 2 | PENSYLVANIAN MERINO | HOT EU | 0,331307306 | -0,887075937 |
| TRANSMER 3 | PENSYLVANIAN MERINO | HOT EU | -0,740918842 | 0,996175004 |
| TRANSMER 4 | PENSYLVANIAN MERINO | HOT EU | -0,47859282 | 0,192070989 |
| TRANSMER 5 | PENSYLVANIAN MERINO | HOT EU | -0,624413154 | 0,951684775 |
| TRANSMER 6 | PENSYLVANIAN MERINO | HOT EU | -0,32580233 | 0,49778289 |
| TRANSMER 7 | PENSYLVANIAN MERINO | HOT EU | -0,6835881 | 0,645453314 |
| TRANSMER 8 | PENSYLVANIAN MERINO | HOT EU | -0,334185129 | -0,132173324 |
| TRANSMER 9 | PENSYLVANIAN MERINO | HOT EU | 2,064362194 | 0,862139031 |
| TRANSMER 10 | PENSYLVANIAN MERINO | HOT EU | -0,852487622 | 1,771159205 |
| TRANSMER 11 | PENSYLVANIAN MERINO | HOT EU | -0,006862559 | 0,139241352 |
| TRANSMER 12 | PENSYLVANIAN MERINO | HOT EU | -0,774878794 | 1,172907249 |
| TRANSMER 13 | PENSYLVANIAN MERINO | HOT EU | -0,551239406 | 0,883706402 |
| TRANSMER 14 | PENSYLVANIAN MERINO | HOT EU | -0,524736538 | 0,733088433 |
| TRANSMER 15 | PENSYLVANIAN MERINO | HOT EU | -0,72216199 | 0,499467009 |
| TRANSMER 16 | PENSYLVANIAN MERINO | HOT EU | -1,15176587 | 0,845170408 |
| TRANSMER 17 | PENSYLVANIAN MERINO | HOT EU | -1,384488589 | 1,842425232 |
| TRANSMER 18 | PENSYLVANIAN MERINO | HOT EU | -1,347397399 | 1,683281264 |
| TRANSMER 19 | PENSYLVANIAN MERINO | HOT EU | -0,122510635 | -0,798648249 |
| TRANSMER 20 | PENSYLVANIAN MERINO | HOT EU | -0,716505279 | 1,126307928 |
| TRANSMER 21 | PENSYLVANIAN MERINO | HOT EU | 0,153601063 | -1,65113218 |
| TRANSMER 22 | PENSYLVANIAN MERINO | HOT EU | -0,998134352 | 1,571828586 |
| TRANSMER 23 | PENSYLVANIAN MERINO | HOT EU | 0,073233704 | 0,088817218 |
| TRANSMER 24 | PENSYLVANIAN MERINO | HOT EU | -0,093005224 | 1,672252395 |
| TRANSMER 25 | PENSYLVANIAN MERINO | HOT EU | -0,921998863 | 0,855128088 |
| TRANSMER 26 | PENSYLVANIAN MERINO | HOT EU | -0,105415184 | -0,442063746 |
| TRANSMER 27 | PENSYLVANIAN MERINO | HOT EU | 0,108828154 | -1,050606289 |
| TRANSMER 28 | PENSYLVANIAN MERINO | HOT EU | -0,47613951 | 0,212121284 |
| TRANSMER 29 | PENSYLVANIAN MERINO | HOT EU | -1,145891998 | 0,955342316 |
| TRANSMER 30 | PENSYLVANIAN MERINO | HOT EU | 3,832514274 | 2,137819836 |
| TRANSMER 31 | PENSYLVANIAN MERINO | HOT EU | -1,065327336 | 0,714456496 |
| TRANSMER 32 | PENSYLVANIAN MERINO | HOT EU | -1,097401154 | 0,914353591 |
| TRANSMER 33 | PENSYLVANIAN MERINO | HOT EU | 0,088344131 | -0,128906056 |
| TRANSMER 34 | PENSYLVANIAN MERINO | HOT EU | -0,46445577 | 1,475314612 |
| TRANSMER 35 | PENSYLVANIAN MERINO | HOT EU | -0,485329498 | 1,454078065 |
| TRANSMER 36 | PENSYLVANIAN MERINO | HOT EU | -0,07784396 | -0,148795272 |
| TRANSMER 37 | PENSYLVANIAN MERINO | HOT EU | -0,498971043 | 0,39455937 |
| TRANSMER 38 | PENSYLVANIAN MERINO | HOT EU | -0,358992003 | -0,06503116 |
| TRANSMER 39 | PENSYLVANIAN MERINO | HOT EU | -0,563652793 | 1,716783586 |
| TRANSMER 40 | PENSYLVANIAN MERINO | HOT EU | -1,038961047 | 2,485998116 |
| TRANSMER 41 | PENSYLVANIAN MERINO | HOT EU | -0,63896602 | 0,036900761 |
| TRANSMER 42 | PENSYLVANIAN MERINO | HOT EU | -0,474019236 | 1,075639312 |
| TRANSMER 43 | PENSYLVANIAN MERINO | HOT EU | -1,073405788 | 2,230550101 |
| TRANSMER 44 | PENSYLVANIAN MERINO | HOT EU | -0,453509628 | -0,265622555 |
| TRANSMER 45 | PENSYLVANIAN MERINO | HOT EU | 0,053004466 | -0,314028595 |
| ROTSI 1 | RO TSIGAI | HOT EU | -0,21788679 | 0,214649083 |
| ROTSI 2 | RO TSIGAI | HOT EU | 0,605830021 | -0,927709885 |
| ROTSI 3 | RO TSIGAI | HOT EU | 0,461664162 | -1,451401566 |
| ROTSI 4 | RO TSIGAI | HOT EU | -1,34333538 | 1,281709317 |
| ROTSI 5 | RO TSIGAI | HOT EU | -1,058828117 | 0,973500099 |
| ROTSI 6 | RO TSIGAI | HOT EU | -0,47736903 | 0,395547019 |
| ROTSI 7 | RO TSIGAI | HOT EU | -0,553831602 | 0,260437537 |
| ROTSI 8 | RO TSIGAI | HOT EU | -0,355957359 | -0,695404586 |
| ROTSI 9 | RO TSIGAI | HOT EU | -0,071076639 | -0,682747909 |
| ROTSI 10 | RO TSIGAI | HOT EU | -0,11250734 | -0,345220768 |
| ROTSI 11 | RO TSIGAI | HOT EU | -0,601110547 | 1,398245737 |
| ROTSI 12 | RO TSIGAI | HOT EU | -0,303944061 | 0,762468847 |
| ROTSI 13 | RO TSIGAI | HOT EU | -0,249188665 | -1,445787239 |
| ROTSI 14 | RO TSIGAI | HOT EU | 0,085848212 | -0,537706688 |
| ROTSI 15 | RO TSIGAI | HOT EU | -0,058020424 | -0,767785208 |
| ROTSI 16 | RO TSIGAI | HOT EU | 0,439777519 | -1,50541487 |
| ROTSI 17 | RO TSIGAI | HOT EU | 0,073859114 | -0,816403165 |
| ROTSI 18 | RO TSIGAI | HOT EU | -1,164655554 | 1,817381576 |
| ROTSI 19 | RO TSIGAI | HOT EU | -0,372320249 | -1,385144465 |
| ROTSI 20 | RO TSIGAI | HOT EU | -0,230647121 | 0,451189566 |
| TUR 1 | TURCANA | COLD | -0,896042022 | 1,685007523 |
| TUR 2 | TURCANA | COLD | 4,774252127 | 0,169986219 |
| TUR 3 | TURCANA | COLD | -0,810796874 | 0,799010691 |
| TUR 4 | TURCANA | COLD | -0,174036217 | -0,642759301 |
| TUR 5 | TURCANA | COLD | -0,335380491 | -1,309241304 |
| TUR 6 | TURCANA | COLD | -1,327464339 | 0,824924745 |
| TUR 7 | TURCANA | COLD | -0,151514892 | -0,391827875 |
| TUR 8 | TURCANA | COLD | 0,745379451 | -2,120763989 |
| TUR 9 | TURCANA | COLD | -0,447686272 | 0,402497845 |
| TUR 10 | TURCANA | COLD | 3,971850643 | 0,423256184 |
| TUR 11 | TURCANA | COLD | -1,036756466 | 0,859732602 |
| TUR 12 | TURCANA | COLD | 3,622827685 | 0,589565201 |
| TUR 13 | TURCANA | COLD | -1,00611914 | 1,044501026 |
| TUR 14 | TURCANA | COLD | -0,916991373 | 1,287251604 |
| TUR 15 | TURCANA | COLD | 3,450041923 | 1,872011548 |
| TUR 16 | TURCANA | COLD | -0,716838799 | -0,130498502 |
| TUR 17 | TURCANA | COLD | 3,294597997 | 3,185163422 |
| TUR 18 | TURCANA | COLD | 0,167450397 | -1,027045554 |
| TUR 19 | TURCANA | COLD | -0,344992685 | 0,505611987 |
| TUR 20 | TURCANA | COLD | -1,270015682 | -0,24636182 |
| TUR 21 | TURCANA | COLD | 0,177949796 | -0,60444532 |
| TUR 22 | TURCANA | COLD | 0,357018318 | -1,460430058 |
| TUR 23 | TURCANA | COLD | 0,214745911 | -2,301229223 |
| TUR 24 | TURCANA | COLD | -0,529113869 | 0,122063084 |
| TUR 25 | TURCANA | COLD | 3,858233242 | 2,639727095 |
| TUR 26 | TURCANA | COLD | -1,221780222 | 1,030898272 |
| TUR 27 | TURCANA | COLD | -0,219760975 | -0,634747015 |
| TUR 28 | TURCANA | COLD | -0,098578225 | 0,656329186 |
| TUR 29 | TURCANA | COLD | -0,752286558 | 0,413690635 |
| TUR 30 | TURCANA | COLD | 0,733882282 | -2,840927581 |
| TUR 31 | TURCANA | COLD | 0,634918288 | -2,462306339 |
| TUR 32 | TURCANA | COLD | -0,376098257 | -0,94128223 |
| TUR 33 | TURCANA | COLD | -0,43503842 | 0,27822267 |
| TUR 34 | TURCANA | COLD | 4,120064663 | -0,023471536 |
| TUR 35 | TURCANA | COLD | -0,081434219 | -0,94181208 |
| TUR 36 | TURCANA | COLD | -0,400023383 | 0,946034267 |
| TUR 37 | TURCANA | COLD | 0,5352087 | -2,382704762 |
| TUR 38 | TURCANA | COLD | 0,084523063 | -1,20479617 |
| TUR 39 | TURCANA | COLD | -0,666776971 | -1,05832453 |
| TUR 40 | TURCANA | COLD | 0,422135557 | -1,454361821 |
| TUR 41 | TURCANA | COLD | 0,877969592 | -2,901261143 |
| TUR 42 | TURCANA | COLD | 0,53372783 | -2,0583437 |
| TUR 43 | TURCANA | COLD | -0,961155627 | 1,024820467 |
| TUR 44 | TURCANA | COLD | -0,093344168 | -0,716142164 |
| TUR 45 | TURCANA | COLD | -0,256444621 | -0,284221173 |
| TUR 46 | TURCANA | COLD | -0,989220496 | 0,623832987 |
| TUR 47 | TURCANA | COLD | 0,210983267 | -0,08261464 |
| TUR 48 | TURCANA | COLD | -0,691355062 | 1,569550939 |
| TUR 49 | TURCANA | COLD | 0,767960231 | -1,866612762 |
| TUR 50 | TURCANA | COLD | 0,393472269 | -1,651671457 |
| TUR 51 | TURCANA | COLD | 0,910471793 | -3,032637691 |
| TUR 52 | TURCANA | COLD | 0,174568798 | -0,977406853 |
| TUR 53 | TURCANA | COLD | -0,795405757 | 1,418739444 |
| TUR 54 | TURCANA | COLD | 0,277466491 | -0,681111002 |
| TUR 55 | TURCANA | COLD | -0,521944372 | -0,065427159 |
| TUR 56 | TURCANA | COLD | 0,030036147 | -0,78223173 |
| TUR 57 | TURCANA | COLD | -0,284641846 | 0,127188993 |
| TUR 58 | TURCANA | COLD | -0,752234469 | 0,573560328 |
| HUMER 1 | HU MERINO | COLD | -1,390261423 | 1,523169889 |
| HUMER 2 | HU MERINO | COLD | -0,827508541 | 1,823640722 |
| HUMER 3 | HU MERINO | COLD | -0,783600642 | 2,016105919 |
| HUMER 4 | HU MERINO | COLD | 0,539663303 | -1,109801869 |
| HUMER 5 | HU MERINO | COLD | -0,624239549 | -0,007259629 |
| HUMER 6 | HU MERINO | COLD | -1,380815935 | 2,251255556 |
| HUMER 7 | HU MERINO | COLD | -1,19680114 | 1,083379789 |
| HUMER 8 | HU MERINO | COLD | -1,031706494 | 2,422463168 |
| HUMER 9 | HU MERINO | COLD | -1,866209573 | 2,743502179 |
| HUMER 10 | HU MERINO | COLD | -0,369928964 | 1,592033536 |
| HUMER 11 | HU MERINO | COLD | -0,436519291 | 0,602583733 |
| HUMER 12 | HU MERINO | COLD | -0,146399431 | 0,515508718 |
| HUMER 13 | HU MERINO | COLD | -0,354416932 | 0,386786949 |
| HUMER 14 | HU MERINO | COLD | -0,993774507 | 1,882345146 |
| HUMER 15 | HU MERINO | COLD | -1,246530378 | 2,206367882 |
| HUMER 16 | HU MERINO | COLD | -1,128013322 | 1,506262653 |
| HUMER 17 | HU MERINO | COLD | -0,795755658 | 1,306209135 |
| HUMER 18 | HU MERINO | COLD | -1,771266903 | 2,779258822 |
| HUMER 19 | HU MERINO | COLD | -0,796130366 | 1,231727971 |
| HUMER 20 | HU MERINO | COLD | -1,023538967 | 1,489478064 |
| HUMER 21 | HU MERINO | COLD | -0,47859282 | 0,192070989 |
| HUMER 22 | HU MERINO | COLD | -1,19680114 | 1,083379789 |
| HUMER 23 | HU MERINO | COLD | -0,284896501 | 0,393600393 |
| HUMER 24 | HU MERINO | COLD | -1,662168233 | 4,019715057 |
| HUMER 25 | HU MERINO | COLD | -1,325791863 | 3,288098514 |
| HUMER 26 | HU MERINO | COLD | 0,54228565 | -1,342837286 |
| HUMER 27 | HU MERINO | COLD | 0,098679882 | -0,690410795 |
| HUMER 28 | HU MERINO | COLD | -1,606494792 | 2,395779881 |
| HUMER 29 | HU MERINO | COLD | -1,341616368 | 3,220466242 |
| HUMER 30 | HU MERINO | COLD | -1,530887306 | 2,06927735 |
| HUMER 31 | HU MERINO | COLD | -1,144756678 | 1,959108687 |
| HUMER 32 | HU MERINO | COLD | -0,530121559 | 1,278982012 |
| HUMER 33 | HU MERINO | COLD | -1,04967354 | 0,958349345 |
| HUMER 34 | HU MERINO | COLD | -0,477415987 | 1,622218554 |
| HUMER 35 | HU MERINO | COLD | 0,041802907 | -0,096694934 |
| AWAS 1 | HU AWASSI | HOT EU | 3,827354908 | 1,344662327 |
| AWAS 2 | HU AWASSI | HOT EU | 0,030036147 | -0,78223173 |
| AWAS 3 | HU AWASSI | HOT EU | -0,424105904 | -0,230493451 |
| AWAS 4 | HU AWASSI | HOT EU | -1,589259748 | 2,750170081 |
| AWAS 5 | HU AWASSI | HOT EU | -1,02538663 | 2,53270593 |
| AWAS 6 | HU AWASSI | HOT EU | -6,741431453 | -0,881073826 |
| AWAS 7 | HU AWASSI | HOT EU | 3,493304333 | 2,263726458 |
| AWAS 8 | HU AWASSI | HOT EU | 0,238952448 | -0,351735066 |
| AWAS 9 | HU AWASSI | HOT EU | 3,534213786 | 1,989228957 |
| AWAS 10 | HU AWASSI | HOT EU | -0,63772558 | 0,605606936 |
| AWAS 11 | HU AWASSI | HOT EU | -0,584969109 | 1,507972531 |
| AWAS 12 | HU AWASSI | HOT EU | 4,31103742 | 1,310269626 |
| AWAS 13 | HU AWASSI | HOT EU | -1,337261535 | 2,337407237 |
| AWAS 14 | HU AWASSI | HOT EU | -0,289024899 | -0,241313516 |
| AWAS 15 | HU AWASSI | HOT EU | 0,640274762 | -0,67226187 |
| AWAS 16 | HU AWASSI | HOT EU | -0,436519291 | 0,602583733 |
| AWAS 17 | HU AWASSI | HOT EU | 0,656739163 | -1,055747358 |
| AWAS 18 | HU AWASSI | HOT EU | -6,477386645 | -1,40804705 |
| AWAS 19 | HU AWASSI | HOT EU | -0,38458325 | 0,654205467 |
| AWAS 20 | HU AWASSI | HOT EU | -1,00611914 | 1,044501026 |
| AWAS 21 | HU AWASSI | HOT EU | -1,071877965 | 2,391468295 |
| AWAS 22 | HU AWASSI | HOT EU | -1,316680089 | 2,134044694 |
| AWAS 23 | HU AWASSI | HOT EU | 0,316017433 | -1,650732184 |
| AWAS 24 | HU AWASSI | HOT EU | -0,724114122 | 1,72971893 |
| AWAS 25 | HU AWASSI | HOT EU | 3,770273043 | 1,697082088 |
| AWAS 26 | HU AWASSI | HOT EU | -0,609256019 | -0,066384055 |
| AWAS 27 | HU AWASSI | HOT EU | -0,508283925 | 0,564729866 |
| AWAS 28 | HU AWASSI | HOT EU | -0,764699945 | 1,246767819 |
| AWAS 29 | HU AWASSI | HOT EU | 3,88411728 | 2,207922877 |
| AWAS 30 | HU AWASSI | HOT EU | -5,757452711 | -3,987348395 |
| AWAS 31 | HU AWASSI | HOT EU | 0,595264297 | -1,137860568 |
| AWAS 32 | HU AWASSI | HOT EU | -0,078662481 | 0,502599702 |
| AWAS 33 | HU AWASSI | HOT EU | 4,097008984 | 1,128241949 |
| AWAS 34 | HU AWASSI | HOT EU | 0,306226394 | -1,050690417 |
| AWAS 35 | HU AWASSI | HOT EU | -1,329906793 | 2,123218083 |
| AWAS 36 | HU AWASSI | HOT EU | 0,144215077 | -0,662584744 |
| AWAS 37 | HU AWASSI | HOT EU | -1,315199218 | 1,809683631 |
| AWAS 38 | HU AWASSI | HOT EU | 3,414287886 | 2,554614605 |
| AWAS 39 | HU AWASSI | HOT EU | -0,508562049 | 0,280120548 |
| AWAS 40 | HU AWASSI | HOT EU | -0,662607615 | 1,359946756 |

Table S6. Genotype of 7 SNPs that is not available at the EVA

| #CHROM | 22 | 18 | 18 | 18 | 18 | 6 | 6 |
| --- | --- | --- | --- | --- | --- | --- | --- |
| POSITION | 36604210 | 64577407 | 64577334 | 64577274 | 64577250 | 86204591 | 86190116 |
| ID | rs161504783 | rs397514117 | rs397514269 | rs397514272 | rs397514273 | rs416941267 | rs420959261 |
| REF | T | C | G | T | G | T | T |
| ALT | C | A | A | G | A | G | C |
| PRA 1 | 1/1 | ./. | ./. | ./. | 0/0 | 1/1 | ./. |
| PRA 2 | ./. | 0/0 | ./. | ./. | ./. | ./. | ./. |
| PRA 3 | 1/1 | 0/0 | ./. | 0/0 | 0/1 | 0/1 | ./. |
| PRA 4 | ./. | ./. | ./. | 0/0 | 0/0 | 0/1 | ./. |
| PRA 5 | 0/1 | ./. | ./. | 0/0 | 0/0 | ./. | ./. |
| PRA 6 | 0/1 | 0/0 | ./. | 0/0 | 0/1 | 0/1 | ./. |
| PRA 7 | 0/1 | 0/0 | ./. | 0/0 | 0/0 | 0/0 | ./. |
| PRA 8 | 0/1 | 0/0 | ./. | 0/0 | 0/1 | 0/1 | ./. |
| PRA 9 | 0/1 | ./. | ./. | ./. | ./. | 0/1 | ./. |
| PRA 10 | 1/1 | ./. | ./. | ./. | ./. | 1/1 | ./. |
| PRA 11 | ./. | ./. | ./. | 0/1 | 0/0 | 0/1 | ./. |
| PRA 12 | 0/1 | 0/0 | ./. | 0/0 | ./. | 1/1 | ./. |
| PRA 13 | 0/0 | 0/0 | ./. | 0/0 | 0/1 | 0/0 | ./. |
| PRA 14 | 0/1 | 0/0 | ./. | 0/0 | 0/0 | 0/1 | ./. |
| PRA 15 | 0/0 | 0/0 | ./. | 0/0 | 0/1 | 1/1 | ./. |
| PRA 16 | 0/1 | 0/0 | ./. | 0/0 | ./. | 0/1 | ./. |
| PRA 17 | 0/1 | 0/0 | ./. | ./. | 0/1 | 1/1 | ./. |
| PRA 18 | 0/0 | 0/0 | ./. | 0/0 | 0/0 | 1/1 | ./. |
| PRA 19 | 0/1 | 0/0 | ./. | 0/0 | 0/1 | 1/1 | ./. |
| PRA 20 | 0/0 | ./. | ./. | 0/0 | ./. | ./. | ./. |
| PRA 21 | 1/1 | 0/0 | ./. | 0/0 | 0/1 | 0/1 | ./. |
| PRA 22 | 1/1 | 0/0 | ./. | 0/0 | 0/0 | 1/1 | ./. |
| PRA 23 | 1/1 | ./. | ./. | ./. | ./. | 0/1 | ./. |
| PRA 24 | 0/1 | ./. | ./. | 0/1 | 0/0 | 0/1 | ./. |
| PRA 25 | 1/1 | ./. | ./. | ./. | 0/1 | 1/1 | ./. |
| PRA 26 | ./. | 0/0 | ./. | 0/0 | ./. | 1/1 | ./. |
| PRA 27 | 0/1 | 0/0 | ./. | 0/0 | 0/0 | 0/0 | ./. |
| PRA 28 | 0/1 | 0/0 | ./. | 0/0 | 0/1 | 1/1 | ./. |
| PRA 29 | 0/1 | 0/0 | ./. | 0/0 | 0/1 | 0/1 | ./. |
| PRA 30 | 0/1 | 0/0 | ./. | 0/0 | 0/0 | 0/1 | ./. |
| PRA 31 | 0/0 | 0/0 | ./. | 0/0 | 0/1 | 1/1 | ./. |
| PRA 32 | ./. | 0/0 | ./. | 0/0 | 0/0 | 0/1 | ./. |
| PRA 33 | 1/1 | 0/0 | ./. | 0/0 | 0/0 | 1/1 | ./. |
| PRA 34 | 0/1 | 0/0 | ./. | 0/1 | 0/0 | 0/1 | ./. |
| PRA 35 | 0/0 | 0/0 | ./. | 0/0 | 0/0 | 1/1 | ./. |
| PRA 36 | 0/1 | 0/0 | ./. | 0/0 | 0/0 | 0/0 | ./. |
| PRA 37 | 0/1 | 0/0 | ./. | 0/0 | 0/1 | 1/1 | ./. |
| BG 1 | 0/1 | ./. | ./. | ./. | ./. | ./. | ./. |
| BG 2 | 0/1 | ./. | ./. | ./. | ./. | ./. | ./. |
| BG 3 | 0/1 | ./. | ./. | ./. | ./. | ./. | ./. |
| BG 4 | 0/1 | ./. | ./. | ./. | ./. | ./. | ./. |
| BG 5 | 0/1 | ./. | ./. | ./. | ./. | ./. | ./. |
| BG 6 | 0/1 | ./. | ./. | ./. | ./. | ./. | ./. |
| BG 7 | 0/1 | ./. | ./. | ./. | ./. | ./. | ./. |
| BG 8 | 0/1 | ./. | ./. | ./. | ./. | ./. | ./. |
| BG 9 | ./. | ./. | ./. | ./. | ./. | ./. | ./. |
| BG 10 | ./. | ./. | ./. | ./. | ./. | ./. | ./. |
| BG 11 | 0/1 | ./. | ./. | ./. | ./. | ./. | ./. |
| BG 12 | 0/1 | ./. | ./. | ./. | ./. | ./. | ./. |
| BG 13 | 0/1 | ./. | ./. | ./. | ./. | ./. | ./. |
| BG 14 | 0/1 | ./. | ./. | ./. | ./. | ./. | ./. |
| BG 15 | 0/1 | ./. | ./. | ./. | ./. | ./. | ./. |
| BG 16 | 0/1 | ./. | ./. | ./. | ./. | ./. | ./. |
| BG 17 | ./. | ./. | ./. | ./. | ./. | ./. | ./. |
| BG 18 | 0/1 | ./. | ./. | ./. | ./. | ./. | ./. |
| BG 19 | 0/1 | ./. | ./. | ./. | ./. | ./. | ./. |
| BG 20 | 0/0 | ./. | ./. | ./. | ./. | ./. | ./. |
| BG 21 | 0/1 | ./. | ./. | ./. | ./. | ./. | ./. |
| BG 22 | 0/1 | ./. | ./. | ./. | ./. | ./. | ./. |
| BG 23 | 0/1 | ./. | ./. | ./. | ./. | ./. | ./. |
| BG 24 | 1/1 | ./. | ./. | ./. | ./. | ./. | ./. |
| BG 25 | ./. | ./. | ./. | ./. | ./. | ./. | ./. |
| BG 26 | 0/1 | ./. | ./. | ./. | ./. | ./. | ./. |
| BG 27 | 0/1 | ./. | ./. | ./. | ./. | ./. | ./. |
| BG 28 | 0/1 | ./. | ./. | ./. | ./. | ./. | ./. |
| BG 29 | ./. | ./. | ./. | ./. | ./. | ./. | ./. |
| DM 1 | 0/1 | ./. | ./. | ./. | ./. | ./. | ./. |
| DM 2 | ./. | ./. | ./. | ./. | ./. | ./. | ./. |
| DM 3 | 0/1 | ./. | ./. | ./. | ./. | ./. | ./. |
| DM 4 | ./. | ./. | ./. | ./. | ./. | ./. | ./. |
| DM 5 | 0/1 | ./. | ./. | ./. | ./. | ./. | ./. |
| DM 6 | 0/1 | ./. | ./. | ./. | ./. | ./. | ./. |
| DM 7 | 0/1 | ./. | ./. | ./. | ./. | ./. | ./. |
| DM 8 | 0/1 | ./. | ./. | ./. | ./. | ./. | ./. |
| DM 9 | ./. | ./. | ./. | ./. | ./. | ./. | ./. |
| DM 10 | ./. | ./. | ./. | ./. | ./. | ./. | ./. |
| DM 11 | 0/1 | ./. | ./. | ./. | ./. | ./. | ./. |
| DM 12 | 0/1 | ./. | ./. | ./. | ./. | ./. | ./. |
| DM 13 | 0/1 | ./. | ./. | ./. | ./. | ./. | ./. |
| DM 14 | 0/1 | ./. | ./. | ./. | ./. | ./. | ./. |
| DM 15 | 0/1 | ./. | ./. | ./. | ./. | ./. | ./. |
| DM 16 | 0/1 | ./. | ./. | ./. | ./. | ./. | ./. |
| DM 17 | ./. | ./. | ./. | ./. | ./. | ./. | ./. |
| DM 18 | 0/0 | ./. | ./. | ./. | ./. | ./. | ./. |
| DM 19 | ./. | ./. | ./. | ./. | ./. | ./. | ./. |
| DM 20 | 0/1 | ./. | ./. | ./. | ./. | ./. | ./. |
| DM 21 | 0/1 | ./. | ./. | ./. | ./. | ./. | ./. |
| DM 22 | 0/1 | ./. | ./. | ./. | ./. | ./. | ./. |
| DM 23 | 0/1 | ./. | ./. | ./. | ./. | ./. | ./. |
| DM 24 | 0/0 | ./. | ./. | ./. | ./. | ./. | ./. |
| DM 25 | ./. | ./. | ./. | ./. | ./. | ./. | ./. |
| DM 26 | 0/0 | ./. | ./. | ./. | ./. | ./. | ./. |
| DM 27 | 0/1 | ./. | ./. | ./. | ./. | ./. | ./. |
| DM 28 | 0/1 | ./. | ./. | ./. | ./. | ./. | ./. |
| DM 29 | 0/1 | ./. | ./. | ./. | ./. | ./. | ./. |
| TIM 1 | 1/1 | ./. | ./. | ./. | ./. | ./. | ./. |
| TIM 2 | ./. | ./. | ./. | 0/0 | ./. | ./. | ./. |
| TIM 3 | 0/1 | 0/0 | ./. | 0/0 | ./. | ./. | ./. |
| TIM 4 | ./. | 0/0 | ./. | 0/0 | ./. | ./. | ./. |
| TIM 5 | 0/1 | 0/0 | ./. | 0/0 | ./. | ./. | ./. |
| TIM 6 | 1/1 | 0/0 | ./. | 0/0 | ./. | ./. | ./. |
| TIM 7 | 0/1 | ./. | ./. | ./. | ./. | ./. | ./. |
| TIM 8 | 0/1 | ./. | ./. | 0/0 | ./. | ./. | ./. |
| TIM 9 | ./. | ./. | ./. | ./. | ./. | ./. | ./. |
| TIM 10 | 0/1 | 0/0 | ./. | 0/0 | ./. | ./. | ./. |
| TIM 11 | ./. | 0/0 | ./. | 0/0 | ./. | ./. | ./. |
| TIM 12 | ./. | 0/0 | ./. | 0/0 | ./. | ./. | ./. |
| TIM 13 | 0/1 | 0/0 | ./. | 0/0 | ./. | ./. | ./. |
| TIM 14 | 1/1 | 0/0 | ./. | 0/0 | ./. | ./. | ./. |
| TIM 15 | 0/1 | 0/0 | ./. | 0/0 | ./. | ./. | ./. |
| TIM 16 | 0/1 | 0/0 | ./. | 0/0 | ./. | ./. | ./. |
| TIM 17 | 0/1 | 0/0 | ./. | 0/0 | ./. | ./. | ./. |
| TIM 18 | 0/1 | 0/0 | ./. | 0/0 | ./. | ./. | ./. |
| TIM 19 | ./. | 0/0 | ./. | 0/0 | ./. | ./. | ./. |
| TIM 20 | 0/1 | 0/0 | ./. | 0/0 | ./. | ./. | ./. |
| TIM 21 | ./. | 0/0 | ./. | 0/0 | ./. | ./. | ./. |
| TIM 22 | 0/1 | 0/0 | ./. | 0/0 | ./. | ./. | ./. |
| TIM 23 | 0/1 | 0/0 | ./. | 0/0 | ./. | ./. | ./. |
| TIM 24 | ./. | 0/0 | ./. | 0/0 | ./. | ./. | ./. |
| TIM 25 | 0/1 | 0/0 | ./. | 0/0 | ./. | ./. | ./. |
| TIM 26 | 0/1 | 0/0 | ./. | 0/0 | ./. | ./. | ./. |
| TIM 27 | ./. | ./. | ./. | ./. | ./. | ./. | ./. |
| TIM 28 | 0/0 | 0/0 | ./. | 0/0 | ./. | ./. | ./. |
| SAR1 | ./. | 0/1 | ./. | ./. | 0/0 | ./. | ./. |
| SAR2 | ./. | 0/1 | ./. | ./. | 0/0 | ./. | ./. |
| SAR3 | ./. | 0/1 | ./. | ./. | 0/0 | ./. | ./. |
| SAR4 | ./. | 0/1 | ./. | ./. | 0/0 | ./. | ./. |
| SAR5 | 1/1 | 0/1 | ./. | ./. | 0/0 | ./. | ./. |
| SAR6 | 1/1 | 0/1 | ./. | ./. | 0/0 | ./. | ./. |
| SAR7 | 1/1 | 0/1 | ./. | ./. | 0/0 | ./. | ./. |
| SAR8 | ./. | 0/1 | ./. | ./. | 0/0 | ./. | ./. |
| SAR9 | ./. | 0/1 | ./. | ./. | 0/0 | ./. | ./. |
| SAR10 | 1/1 | 0/1 | ./. | ./. | 0/0 | ./. | ./. |
| SAR11 | 1/1 | 0/1 | ./. | ./. | 0/0 | ./. | ./. |
| SAR12 | ./. | 0/1 | ./. | ./. | 0/0 | ./. | ./. |
| SAR13 | 1/1 | 0/1 | ./. | ./. | 0/0 | ./. | ./. |
| SAR14 | ./. | 0/1 | ./. | ./. | 0/0 | ./. | ./. |
| SAR15 | ./. | ./. | ./. | ./. | 0/0 | ./. | ./. |
| SAR16 | ./. | ./. | ./. | ./. | 0/0 | ./. | ./. |
| SAR17 | 1/1 | ./. | ./. | ./. | 0/0 | ./. | ./. |
| SAR18 | 1/1 | 0/1 | ./. | ./. | 0/0 | ./. | ./. |
| SAR19 | ./. | 0/1 | ./. | ./. | 0/0 | ./. | ./. |
| SAR20 | ./. | ./. | ./. | ./. | 0/0 | ./. | ./. |
| SAR21 | ./. | ./. | ./. | ./. | 0/0 | ./. | ./. |
| SAR22 | 1/1 | 0/1 | ./. | ./. | 0/0 | ./. | ./. |
| SAR23 | ./. | ./. | ./. | ./. | 0/0 | ./. | ./. |
| SAR24 | 0/1 | ./. | ./. | ./. | 0/0 | ./. | ./. |
| SAR25 | ./. | 0/1 | ./. | ./. | ./. | ./. | ./. |
| SAR26 | 1/1 | 0/1 | ./. | ./. | 0/0 | ./. | ./. |
| SAR27 | 1/1 | 0/1 | ./. | ./. | 0/0 | ./. | ./. |
| SAR28 | 0/1 | 0/1 | ./. | ./. | 0/0 | ./. | ./. |
| SAR29 | 0/1 | 0/1 | ./. | ./. | ./. | ./. | ./. |
| SUF 1 | 0/1 | 0/0 | 1/1 | 0/0 | 0/0 | 0/1 | 0/0 |
| SUF 2 | 0/1 | 0/0 | 0/1 | 0/0 | 0/0 | 0/1 | 0/0 |
| SUF 3 | 0/0 | 0/0 | 1/1 | 0/0 | 0/0 | 1/1 | 0/0 |
| SUF 4 | 1/1 | 0/0 | 0/1 | 0/0 | 0/0 | 0/1 | 0/0 |
| SUF 5 | 0/1 | 0/0 | 0/1 | 0/0 | 0/0 | 1/1 | 0/0 |
| SUF 6 | 0/1 | 0/0 | 0/1 | 0/0 | 0/0 | 1/1 | 0/0 |
| SUF 7 | 1/1 | 0/0 | 0/1 | 0/0 | 0/0 | 1/1 | 0/0 |
| SUF 8 | 0/1 | 0/0 | 0/1 | 0/0 | 0/0 | 0/1 | 0/1 |
| SUF 9 | 0/1 | 0/0 | 0/1 | 0/0 | 0/0 | 0/0 | 0/0 |
| SUF 10 | 0/1 | 0/0 | 0/1 | 0/0 | 0/0 | 1/1 | 0/1 |
| SUF 11 | 0/1 | 0/0 | 0/0 | 0/0 | 0/0 | 1/1 | 0/1 |
| SUF 12 | 1/1 | 0/0 | 0/0 | 0/0 | 0/0 | 0/1 | 0/1 |
| SUF 13 | 0/1 | 0/0 | 0/0 | 0/0 | 0/0 | 0/1 | 0/0 |
| SUF 14 | 1/1 | 0/0 | 0/1 | 0/0 | 0/0 | 1/1 | 0/1 |
| SUF 15 | 0/0 | 0/0 | 1/1 | 0/0 | 0/0 | 0/1 | 0/0 |
| SUF 16 | 1/1 | 0/0 | 0/1 | 0/0 | 0/0 | 1/1 | 0/0 |
| SUF 17 | 0/1 | 0/0 | 0/1 | 0/0 | 0/0 | 0/1 | 0/0 |
| SUF 18 | 0/1 | 0/0 | 1/1 | 0/0 | 0/0 | 0/1 | 0/0 |
| SUF 19 | 1/1 | 0/0 | 0/1 | 0/0 | 0/0 | 1/1 | 0/0 |
| SUF 20 | 1/1 | 0/0 | 0/1 | 0/0 | 0/0 | 1/1 | 0/0 |
| SUF 21 | 1/1 | 0/1 | 1/1 | 0/1 | 0/0 | 1/1 | 0/0 |
| SUF 22 | 0/0 | 0/0 | 0/1 | 0/0 | 0/0 | 1/1 | 0/0 |
| SUF 23 | 0/0 | 0/0 | 1/1 | 0/0 | 0/1 | 0/1 | 1/1 |
| SUF 24 | 1/1 | 0/0 | 0/1 | 0/0 | 0/0 | 1/1 | 0/0 |
| SUF 25 | 0/1 | 0/0 | 0/1 | 0/0 | 0/0 | 1/1 | 0/0 |
| SUF 26 | 0/0 | 0/0 | 0/1 | 0/0 | 0/1 | 0/1 | 0/1 |
| BTET 1 | 0/1 | 0/0 | 1/1 | 0/0 | 0/1 | 0/1 | 0/1 |
| BTET 2 | 1/1 | 0/0 | 0/0 | 0/0 | 0/0 | 1/1 | 0/0 |
| BTET 3 | 0/0 | 0/1 | 1/1 | 0/1 | 0/1 | 0/1 | 0/1 |
| BTET 4 | 0/0 | 0/0 | ./. | 0/0 | 0/0 | 0/1 | 0/1 |
| BTET 5 | 0/0 | 0/0 | ./. | 0/0 | 0/1 | 0/1 | 0/1 |
| BTET 6 | 0/0 | 0/0 | 1/1 | 0/0 | 0/1 | ./. | 0/1 |
| BTET 7 | 0/1 | 0/0 | 1/1 | 0/0 | 1/1 | 0/1 | 0/1 |
| BTET 8 | 0/0 | 0/0 | 0/1 | 0/0 | ./. | 0/0 | 0/0 |
| BTET 9 | 0/0 | 0/0 | 1/1 | 0/0 | 0/1 | 1/1 | 0/0 |
| BTET 10 | 0/1 | 0/0 | 1/1 | 0/0 | 0/1 | 1/1 | 0/0 |
| BTET 11 | 0/1 | 0/0 | 1/1 | 0/0 | 0/1 | 1/1 | 0/0 |
| BTET 12 | 0/1 | 0/0 | 1/1 | 0/0 | 0/1 | 1/1 | 0/0 |
| BTET 13 | 1/1 | 0/0 | 1/1 | 0/0 | 0/1 | 1/1 | 0/0 |
| BTET 14 | 0/1 | 0/0 | 0/1 | 0/0 | 0/0 | 1/1 | 0/0 |
| BTET 15 | 0/0 | 0/0 | 0/1 | 0/0 | 0/1 | 0/0 | 0/1 |
| BTET 16 | 0/0 | 0/0 | 1/1 | 0/0 | 0/1 | 0/1 | 0/1 |
| BTET 17 | 0/1 | 0/0 | 0/1 | 0/0 | 0/0 | 1/1 | 0/0 |
| BTET 18 | 0/0 | 0/0 | 0/1 | 0/0 | 0/0 | 0/0 | 1/1 |
| BTET 19 | 0/1 | 0/1 | 1/1 | 0/1 | 0/0 | 1/1 | 0/0 |
| BTET 20 | 1/1 | 0/0 | 0/0 | 0/0 | 0/0 | 1/1 | 0/0 |
| BTET 21 | 0/1 | 0/0 | 0/1 | 0/0 | 0/0 | 1/1 | 0/0 |
| BTET 22 | 0/0 | 0/1 | 1/1 | 0/1 | 0/0 | 0/1 | 0/0 |
| BTET 23 | 0/1 | 0/0 | 1/1 | 0/0 | 0/1 | 0/1 | 0/1 |
| BTET 24 | 0/0 | 0/0 | 0/1 | 0/0 | 0/1 | 1/1 | 0/0 |
| BTET 25 | 0/1 | 0/0 | 0/1 | 0/0 | 0/1 | 1/1 | 0/0 |
| BTET 26 | 0/1 | 0/0 | 0/1 | 0/0 | 0/0 | 1/1 | 0/0 |
| BTET 27 | 0/1 | 0/0 | 1/1 | 0/0 | 0/1 | 1/1 | 0/0 |
| BTET 28 | 0/1 | 0/0 | 1/1 | 0/0 | 0/1 | 1/1 | 0/0 |
| BTET 29 | 0/0 | 0/1 | 0/1 | 0/1 | 0/0 | 1/1 | 0/0 |
| BTET 30 | 0/1 | 0/0 | 0/1 | 0/0 | 0/1 | 1/1 | 0/1 |
| BTET 31 | 1/1 | 0/0 | 1/1 | 0/0 | 0/1 | 1/1 | 0/1 |
| BTET 32 | 0/0 | 0/1 | 0/1 | 0/1 | 0/0 | 0/0 | 0/1 |
| BTET 33 | 0/1 | 0/0 | 1/1 | 0/0 | 0/0 | 1/1 | 0/1 |
| BTET 34 | 0/1 | 0/0 | 0/1 | 0/0 | 0/1 | 0/0 | 0/0 |
| BTET 35 | 0/0 | 0/0 | 1/1 | 0/0 | 0/0 | 0/1 | 0/0 |
| BTET 36 | 0/1 | 0/0 | 0/1 | 0/0 | 0/0 | 1/1 | 0/0 |
| IDF 1 | 0/1 | 0/0 | 1/1 | 0/0 | 0/1 | 0/1 | 0/0 |
| IDF 2 | 0/0 | 0/0 | 1/1 | 0/0 | 1/1 | 0/1 | 0/0 |
| IDF 3 | 0/0 | 0/0 | 1/1 | 0/0 | 0/1 | 0/0 | 0/0 |
| IDF 4 | 0/1 | 0/0 | 1/1 | 0/0 | 0/1 | 0/0 | 0/0 |
| IDF 5 | 0/1 | 0/0 | 1/1 | 0/0 | 0/1 | 0/1 | 0/1 |
| IDF 6 | 0/0 | 0/0 | 1/1 | 0/0 | 0/1 | 0/0 | 0/0 |
| IDF 7 | 0/1 | 0/0 | 0/0 | 0/0 | 0/0 | 0/0 | 0/0 |
| IDF 8 | 0/1 | 0/0 | 1/1 | 0/0 | 0/1 | 0/0 | 0/0 |
| IDF 9 | 0/1 | 0/0 | 1/1 | 0/0 | 0/1 | 0/1 | 0/0 |
| IDF 10 | 0/1 | 0/0 | 0/1 | 0/0 | 0/0 | 0/0 | 0/0 |
| IDF 11 | 0/0 | 0/1 | 1/1 | 0/1 | 0/1 | 0/0 | 0/1 |
| IDF 12 | 0/0 | 0/0 | 1/1 | 0/0 | 1/1 | 1/1 | 0/0 |
| IDF 13 | 1/1 | 0/0 | 1/1 | 0/0 | 0/1 | 0/0 | 0/1 |
| IDF 14 | 1/1 | 0/0 | 0/1 | 0/0 | 0/1 | 0/1 | 0/0 |
| IDF 15 | 1/1 | 0/0 | 0/1 | 0/0 | 0/0 | 1/1 | 0/0 |
| IDF 16 | 0/1 | 0/0 | 0/0 | 0/0 | 0/0 | 0/0 | 0/0 |
| IDF 17 | 0/1 | 0/0 | 1/1 | 0/0 | 0/1 | 0/1 | 0/0 |
| IDF 18 | 0/0 | 0/0 | 0/1 | 0/0 | 0/1 | 0/0 | 0/0 |
| IDF 19 | 0/0 | 0/0 | 1/1 | 0/0 | 0/0 | 0/0 | 0/0 |
| IDF 20 | 0/1 | 0/0 | 1/1 | 0/0 | 0/1 | 0/0 | 0/0 |
| IDF 21 | 1/1 | 0/0 | 1/1 | 0/0 | 1/1 | 0/0 | 0/0 |
| HUTSI 1 | 0/1 | 0/0 | 1/1 | 0/0 | ./. | 0/0 | 0/0 |
| HUTSI 2 | 0/1 | 0/0 | 1/1 | 0/0 | 0/1 | 1/1 | 0/1 |
| HUTSI 3 | 1/1 | 0/0 | 0/1 | 0/0 | ./. | 0/1 | 0/0 |
| HUTSI 4 | 1/1 | 0/0 | 0/1 | 0/0 | 0/0 | 1/1 | 0/0 |
| HUTSI 5 | 0/0 | 0/0 | 1/1 | 0/0 | 0/0 | 1/1 | 1/1 |
| HUTSI 6 | 0/1 | 0/0 | 0/0 | 0/0 | 0/0 | 0/1 | 0/0 |
| HUTSI 7 | 0/0 | 0/0 | 0/1 | 0/0 | ./. | 1/1 | ./. |
| HUTSI 8 | 0/1 | 0/0 | 0/1 | 0/0 | 0/1 | 1/1 | 0/1 |
| HUTSI 9 | 0/1 | 0/0 | 0/1 | 0/0 | 0/0 | 0/1 | 0/1 |
| HUTSI 10 | 0/1 | 0/1 | 1/1 | 0/1 | 0/0 | 0/1 | ./. |
| HUTSI 11 | 0/1 | 0/0 | 0/1 | 0/0 | 0/0 | 0/0 | 0/1 |
| HUTSI 12 | 1/1 | 0/0 | 0/1 | 0/0 | 0/0 | 0/0 | 0/1 |
| HUTSI 13 | 0/1 | 0/0 | 1/1 | 0/0 | 0/0 | ./. | 0/0 |
| HUTSI 14 | 1/1 | 0/1 | 1/1 | 0/1 | 0/0 | 1/1 | 0/1 |
| HUTSI 15 | 0/1 | 0/1 | 1/1 | 0/1 | 0/0 | 0/1 | 0/0 |
| HUTSI 16 | 0/1 | 0/1 | 1/1 | 0/1 | 0/0 | 1/1 | ./. |
| HUTSI 17 | 1/1 | 0/0 | 0/1 | 0/0 | 0/0 | 1/1 | 1/1 |
| HUTSI 18 | 0/1 | 0/0 | 0/0 | 0/0 | 0/0 | 1/1 | 1/1 |
| HUTSI 19 | 0/1 | 0/0 | 0/1 | 0/0 | 0/0 | 1/1 | 1/1 |
| HUTSI 20 | 0/1 | 0/0 | 0/1 | 0/0 | ./. | 0/1 | 0/1 |
| HUTSI 21 | 0/1 | 0/0 | 0/0 | 0/0 | 0/0 | 1/1 | 1/1 |
| HUTSI 22 | 0/1 | 0/1 | 1/1 | 0/1 | 0/0 | 1/1 | 0/1 |
| HUTSI 23 | 0/0 | 0/0 | 0/1 | 0/0 | 0/0 | 1/1 | 1/1 |
| HUTSI 24 | 0/1 | 0/0 | 0/1 | 0/0 | 0/0 | 1/1 | 0/1 |
| HUTSI 25 | 1/1 | 0/0 | 0/0 | 0/0 | 0/0 | 1/1 | 0/1 |
| HUTSI 26 | 0/1 | 0/0 | 0/1 | 0/0 | 0/0 | 0/1 | 0/1 |
| HUTSI 27 | 0/1 | 0/0 | 1/1 | 0/0 | 0/0 | 0/0 | 1/1 |
| HUTSI 28 | 0/1 | 0/0 | 0/0 | 0/0 | 0/0 | 1/1 | 0/1 |
| HUTSI 29 | 0/1 | 0/0 | 0/1 | 0/0 | 0/0 | 1/1 | 1/1 |
| HUTSI 30 | 1/1 | 0/1 | 1/1 | 0/1 | 0/0 | 0/1 | 0/1 |
| HUTSI 31 | 0/1 | 0/0 | 0/0 | 0/0 | ./. | 0/1 | 0/1 |
| HUTSI 32 | 0/0 | 0/0 | 0/1 | 0/0 | 0/0 | 0/1 | 1/1 |
| HUTSI 33 | 1/1 | ./. | ./. | 0/1 | 0/0 | 1/1 | 0/1 |
| HURAC 1 | 0/1 | 0/0 | 1/1 | 0/0 | 0/0 | 0/1 | 0/1 |
| HURAC 2 | 0/0 | 0/0 | 0/1 | 0/0 | 0/1 | 0/1 | 0/1 |
| HURAC 3 | 0/1 | 0/0 | 1/1 | 0/0 | 1/1 | 0/1 | ./. |
| HURAC 4 | 0/1 | 0/0 | 1/1 | 0/0 | 1/1 | 0/1 | 0/1 |
| HURAC 5 | 1/1 | 0/0 | 0/1 | 0/0 | 0/0 | 0/1 | 0/1 |
| HURAC 6 | 1/1 | 0/0 | 0/1 | 0/0 | 0/0 | 1/1 | 0/0 |
| HURAC 7 | 0/0 | 0/0 | 0/1 | 0/0 | 0/0 | 1/1 | 0/0 |
| HURAC 8 | 0/1 | 0/0 | 0/1 | 0/0 | 0/1 | 0/1 | 0/1 |
| HURAC 9 | 0/0 | 0/0 | 1/1 | 0/0 | 1/1 | 0/1 | 0/1 |
| HURAC 10 | 0/1 | 0/0 | 0/1 | 0/0 | 0/0 | 0/1 | 0/1 |
| HURAC 11 | 0/1 | 0/0 | 0/1 | 0/0 | ./. | 1/1 | 0/0 |
| HURAC 12 | 0/0 | 0/0 | 0/1 | 0/0 | 0/1 | 0/1 | 0/1 |
| HURAC 13 | 1/1 | 0/0 | 0/1 | 0/0 | ./. | 0/1 | 0/1 |
| HURAC 14 | 0/1 | 0/0 | 1/1 | 0/0 | ./. | 0/1 | 0/1 |
| HURAC 15 | 0/1 | 0/0 | 1/1 | 0/0 | 0/1 | 1/1 | 0/0 |
| HURAC 16 | 1/1 | 0/0 | 1/1 | 0/0 | ./. | 1/1 | 0/0 |
| HURAC 17 | 0/1 | 0/0 | 1/1 | 0/0 | 1/1 | 1/1 | 0/0 |
| HURAC 18 | 1/1 | 0/0 | 1/1 | 0/0 | 1/1 | 1/1 | 0/0 |
| HURAC 19 | 0/1 | 0/0 | 1/1 | 0/0 | 1/1 | 0/0 | 1/1 |
| HURAC 20 | 0/1 | 0/0 | 1/1 | 0/0 | 1/1 | 1/1 | 0/0 |
| HURAC 21 | 0/1 | 0/0 | 1/1 | 0/0 | 1/1 | 1/1 | 0/0 |
| HURAC 22 | 1/1 | 0/0 | 1/1 | 0/0 | 0/0 | 1/1 | 0/0 |
| HURAC 23 | 1/1 | 0/0 | 1/1 | 0/0 | 0/1 | 0/1 | 0/1 |
| HURAC 24 | 0/1 | 0/0 | 0/1 | 0/0 | 0/1 | 0/0 | 1/1 |
| HURAC 25 | 1/1 | 0/0 | 1/1 | 0/0 | 0/1 | 1/1 | 0/0 |
| HURAC 26 | 0/0 | 0/0 | 0/1 | 0/0 | 0/1 | 0/1 | 0/1 |
| HURAC 27 | 0/0 | 0/0 | 0/1 | 0/0 | ./. | 1/1 | 0/0 |
| HURAC 28 | 0/0 | 0/0 | 0/0 | 0/0 | 0/0 | 0/1 | 0/1 |
| HURAC 29 | 0/0 | 0/0 | 0/1 | 0/0 | 0/0 | 0/1 | 0/1 |
| HURAC 30 | 0/0 | 0/0 | 0/1 | 0/0 | 0/0 | 1/1 | 0/0 |
| HURAC 31 | 0/0 | 0/0 | 0/1 | 0/0 | 0/0 | 1/1 | 0/0 |
| HURAC 32 | 0/0 | 0/0 | 0/1 | 0/0 | 0/1 | 0/1 | 0/1 |
| HURAC 33 | 0/0 | 0/0 | 1/1 | 0/0 | 1/1 | 1/1 | 0/0 |
| HURAC 34 | 0/0 | 0/0 | 0/1 | 0/0 | 0/0 | 0/0 | 1/1 |
| HURAC 35 | 0/1 | 0/0 | 1/1 | 0/0 | 1/1 | 0/1 | 0/1 |
| HURAC 36 | 0/0 | 0/0 | 0/1 | 0/0 | 0/1 | 0/1 | 0/1 |
| HURAC 37 | 0/0 | 0/0 | 1/1 | 0/0 | 0/1 | 1/1 | 0/0 |
| HURAC 38 | 0/0 | 0/0 | 1/1 | 0/0 | 0/1 | 0/1 | 0/1 |
| HURAC 39 | 0/0 | 0/0 | 0/0 | 0/0 | 0/0 | 0/1 | 0/1 |
| HURAC 40 | 0/0 | 0/1 | 0/1 | 0/1 | 0/0 | 0/1 | 0/1 |
| HURAC 41 | 0/0 | 0/0 | ./. | 0/0 | 0/1 | 1/1 | 0/0 |
| HURAC 42 | 0/1 | 0/0 | 1/1 | 0/0 | 1/1 | 0/0 | 1/1 |
| HURAC 43 | 0/0 | 0/0 | 1/1 | 0/0 | ./. | 0/1 | 0/1 |
| HURAC 44 | 0/0 | 0/0 | 1/1 | 0/0 | 1/1 | 1/1 | 0/0 |
| HURAC 45 | 0/0 | 0/0 | 0/1 | 0/0 | 0/1 | 0/1 | 0/1 |
| HURAC 46 | 0/1 | 0/1 | 0/1 | 0/1 | 0/0 | 0/1 | 0/1 |
| HURAC 47 | 0/0 | 0/1 | 1/1 | 0/1 | 0/1 | 0/1 | 0/1 |
| HURAC 48 | 0/0 | 0/0 | 1/1 | 0/0 | 0/1 | 0/1 | 0/1 |
| KAR 1 | 0/1 | ./. | 0/1 | 0/0 | 0/0 | 1/1 | 0/0 |
| KAR 2 | 0/1 | 0/0 | 0/1 | 0/0 | ./. | 0/1 | ./. |
| KAR 3 | ./. | 0/0 | ./. | ./. | ./. | ./. | ./. |
| KAR 4 | 1/1 | 0/0 | ./. | 0/0 | ./. | ./. | ./. |
| KAR 5 | 1/1 | 0/0 | ./. | ./. | ./. | ./. | ./. |
| KAR 6 | ./. | ./. | ./. | ./. | ./. | ./. | ./. |
| KAR 7 | 0/0 | ./. | ./. | ./. | ./. | ./. | ./. |
| KAR 8 | ./. | ./. | ./. | 0/1 | ./. | ./. | ./. |
| KAR 9 | ./. | ./. | ./. | ./. | ./. | ./. | ./. |
| KAR 10 | ./. | 0/0 | ./. | ./. | ./. | 1/1 | ./. |
| KAR 11 | ./. | 0/0 | 0/0 | 0/0 | ./. | 1/1 | ./. |
| KAR 12 | 0/0 | 0/1 | 1/1 | ./. | ./. | 1/1 | 0/0 |
| KAR 13 | 0/0 | 0/0 | 0/0 | 0/0 | 0/0 | 1/1 | 0/0 |
| KAR 14 | 0/0 | ./. | ./. | ./. | ./. | ./. | ./. |
| KAR 15 | 0/0 | 0/0 | ./. | ./. | ./. | ./. | ./. |
| KAR 16 | 0/1 | 0/1 | 1/1 | 0/1 | 0/0 | 0/1 | ./. |
| KAR 17 | 0/0 | 0/0 | 0/1 | 0/0 | 0/0 | 0/0 | 0/1 |
| KAR 18 | 0/1 | 0/0 | 1/1 | 0/0 | ./. | 1/1 | 0/0 |
| KAR 19 | ./. | ./. | ./. | ./. | ./. | ./. | 0/0 |
| KAR 20 | 0/0 | 0/0 | ./. | ./. | ./. | 1/1 | 0/0 |
| KAR 21 | ./. | 0/0 | 0/1 | ./. | ./. | 1/1 | ./. |
| KAR 22 | 0/0 | 0/0 | 1/1 | 0/0 | 0/0 | 1/1 | 0/0 |
| KAR 23 | 0/0 | 0/0 | ./. | 0/0 | ./. | 1/1 | 0/0 |
| KAR 24 | 0/1 | 0/0 | ./. | 0/0 | ./. | 1/1 | 0/0 |
| KAR 25 | 0/0 | 0/1 | 1/1 | 0/1 | 0/0 | 0/1 | 0/1 |
| RORAC 1 | 0/1 | 0/0 | 1/1 | 0/0 | 0/1 | 0/1 | 1/1 |
| RORAC 2 | 0/1 | 0/0 | 1/1 | 0/0 | 1/1 | 1/1 | 0/0 |
| RORAC 3 | 0/1 | 0/1 | 1/1 | 0/1 | 0/0 | 0/1 | 0/1 |
| RORAC 4 | 0/1 | 0/1 | 1/1 | 0/1 | 0/0 | 0/0 | 0/1 |
| RORAC 5 | 1/1 | 0/0 | 1/1 | 0/0 | 1/1 | 0/1 | 1/1 |
| RORAC 6 | 0/0 | 0/0 | 1/1 | 0/0 | 1/1 | 0/1 | 0/1 |
| RORAC 7 | 0/1 | 0/0 | 1/1 | 0/0 | 1/1 | 0/1 | 0/1 |
| RORAC 8 | 0/1 | 0/0 | 1/1 | 0/0 | 0/1 | 1/1 | 0/0 |
| RORAC 9 | 1/1 | 0/0 | 0/1 | 0/0 | 0/1 | 0/1 | 0/1 |
| RORAC 10 | 0/1 | 0/0 | 0/1 | 0/0 | 0/0 | 0/0 | 1/1 |
| RORAC 11 | 0/1 | 0/0 | 1/1 | 0/0 | 0/1 | 0/0 | 0/1 |
| RORAC 12 | 0/1 | 0/0 | 1/1 | 0/0 | 0/1 | 0/0 | 1/1 |
| RORAC 13 | 0/0 | 0/0 | 1/1 | 0/0 | 1/1 | 0/1 | 0/1 |
| RORAC 14 | 0/1 | 0/1 | ./. | 0/1 | 0/0 | 0/1 | 0/0 |
| RORAC 15 | 0/0 | 0/0 | 1/1 | 0/0 | 1/1 | 0/1 | 0/1 |
| RORAC 16 | 1/1 | 0/0 | 1/1 | 0/0 | 1/1 | 1/1 | 0/1 |
| RORAC 17 | 0/0 | 0/1 | 1/1 | 0/1 | 0/1 | 0/1 | 0/1 |
| RORAC 18 | 0/1 | 0/0 | 0/1 | 0/0 | 0/0 | 0/1 | 0/1 |
| RORAC 19 | 0/0 | 0/0 | 1/1 | 0/0 | 1/1 | 0/1 | 0/1 |
| RORAC 20 | 0/1 | 0/0 | 1/1 | 0/0 | 0/0 | 0/1 | 0/1 |
| RORAC 21 | 1/1 | 0/1 | 1/1 | 0/1 | 0/1 | 0/1 | 0/1 |
| RORAC 22 | 1/1 | 0/0 | 0/1 | 0/0 | 0/1 | 0/1 | 0/1 |
| RORAC 23 | 0/0 | 0/0 | 0/1 | 0/0 | 0/1 | 0/1 | 0/1 |
| RORAC 24 | 0/1 | 0/0 | 1/1 | 0/0 | 0/0 | 1/1 | 0/0 |
| RORAC 25 | 0/1 | 0/0 | 1/1 | 0/0 | 1/1 | 0/0 | 1/1 |
| RORAC 26 | 1/1 | 0/1 | 1/1 | 0/1 | 0/1 | 0/1 | 0/1 |
| RORAC 27 | 1/1 | 0/1 | 1/1 | 0/1 | 0/0 | 0/1 | 0/0 |
| RORAC 28 | 0/1 | 0/0 | 0/1 | 0/0 | 0/1 | 0/1 | 0/0 |
| RORAC 29 | 0/1 | 0/0 | 0/1 | 0/0 | 0/0 | 0/1 | 1/1 |
| RORAC 30 | 0/1 | 0/0 | 0/1 | 0/0 | 0/1 | 0/0 | 0/1 |
| RORAC 31 | ./. | 0/0 | 1/1 | 0/0 | 1/1 | 0/1 | 0/1 |
| RORAC 32 | 0/1 | 0/0 | 0/1 | 0/0 | 0/0 | 0/1 | 0/1 |
| RORAC 33 | 1/1 | 0/1 | 0/1 | 0/1 | 0/0 | 0/1 | 0/0 |
| RORAC 34 | 0/1 | 0/0 | 1/1 | 0/0 | 0/1 | 0/0 | 1/1 |
| RORAC 35 | 0/1 | 0/0 | 1/1 | 0/0 | 0/0 | 0/1 | 0/1 |
| RORAC 36 | 1/1 | 0/0 | 0/1 | 0/0 | 0/0 | 0/1 | 0/0 |
| RORAC 37 | 0/1 | 0/0 | 0/1 | 0/0 | 0/1 | 0/1 | 0/1 |
| RORAC 38 | 0/1 | 0/0 | 1/1 | 0/0 | 1/1 | 1/1 | 0/0 |
| RORAC 39 | 0/0 | 0/0 | 1/1 | 0/0 | 1/1 | 0/1 | 0/1 |
| RORAC 40 | 1/1 | 0/0 | 0/1 | 0/0 | 0/1 | 0/1 | 0/0 |
| RORAC 41 | 1/1 | 0/0 | 0/0 | 0/0 | 0/0 | 0/1 | 0/0 |
| RORAC 42 | 0/1 | 0/0 | 0/1 | 0/0 | 0/1 | 0/1 | 0/1 |
| RORAC 43 | 0/1 | 0/0 | 1/1 | 0/0 | 0/1 | 0/1 | 1/1 |
| RORAC 44 | 0/0 | 0/0 | 1/1 | 0/0 | 0/1 | 0/0 | 1/1 |
| RORAC 45 | 0/0 | 1/1 | 1/1 | 1/1 | 0/0 | 0/1 | 0/1 |
| RORAC 46 | 0/1 | 1/1 | 1/1 | 1/1 | 0/0 | 0/1 | 0/1 |
| RORAC 47 | 0/1 | 0/1 | 1/1 | 0/1 | 0/0 | 0/1 | 0/1 |
| RORAC 48 | 0/1 | 0/0 | 0/1 | 0/0 | 0/1 | 0/1 | 0/1 |
| RORAC 49 | 1/1 | 0/0 | 1/1 | 0/0 | 0/1 | 0/0 | 1/1 |
| RORAC 50 | 0/1 | 0/0 | 0/1 | 0/0 | 0/0 | 1/1 | 0/1 |
| RORAC 51 | 0/1 | 0/0 | 1/1 | 0/0 | 0/0 | 1/1 | 0/0 |
| RORAC 52 | 1/1 | 0/0 | 1/1 | 0/0 | 0/0 | 1/1 | 0/0 |
| RORAC 53 | 0/1 | 0/0 | 1/1 | 0/0 | 1/1 | 0/0 | 1/1 |
| RORAC 54 | 0/1 | 0/1 | 0/1 | 0/1 | 0/0 | 0/1 | 0/1 |
| RORAC 55 | 0/1 | 0/0 | 1/1 | 0/0 | 0/1 | 1/1 | 0/0 |
| RORAC 56 | 1/1 | 0/0 | 1/1 | 0/0 | 0/0 | 0/0 | 1/1 |
| RORAC 57 | 0/1 | 0/1 | 1/1 | 0/1 | 0/1 | 1/1 | 0/0 |
| RORAC 58 | 0/0 | 0/0 | 0/1 | 0/0 | ./. | 1/1 | 0/0 |
| RORAC 59 | 0/1 | 0/0 | 1/1 | 0/0 | 1/1 | 0/1 | 0/1 |
| RORAC 60 | 0/0 | 0/0 | 1/1 | 0/0 | 1/1 | 1/1 | 0/0 |
| RORAC 61 | 0/1 | 0/0 | 1/1 | 0/0 | 0/1 | 1/1 | 0/0 |
| RORAC 62 | 0/1 | 0/0 | 1/1 | 0/0 | 1/1 | 1/1 | 0/0 |
| TRANME 1 | 0/0 | 0/0 | 1/1 | 0/0 | 0/0 | 1/1 | 0/0 |
| TRANME 2 | 0/1 | 0/0 | 1/1 | 0/0 | 0/0 | 0/0 | 0/1 |
| TRANME 3 | 0/0 | 0/0 | 0/1 | 0/0 | 0/1 | 1/1 | 0/0 |
| TRANME 4 | 0/1 | 0/0 | 1/1 | 0/0 | 0/0 | 1/1 | 0/0 |
| TRANME 5 | 1/1 | 0/0 | 0/1 | 0/0 | 0/1 | 1/1 | 0/0 |
| TRANME 6 | 0/0 | 0/0 | 1/1 | ./. | ./. | 1/1 | 0/0 |
| TRANME 7 | 1/1 | 0/0 | ./. | 0/0 | ./. | 1/1 | 0/0 |
| TRANME 8 | 0/1 | 0/0 | 1/1 | 0/0 | 0/1 | 1/1 | 0/0 |
| TRANME 9 | ./. | 0/1 | ./. | 0/0 | ./. | 0/0 | 0/0 |
| TRANME 10 | 1/1 | 0/0 | 0/1 | 0/0 | 0/0 | 1/1 | 0/0 |
| TRANME 11 | 1/1 | 0/0 | 0/1 | 0/0 | 0/0 | 0/1 | 0/0 |
| TRANME 12 | 1/1 | 0/0 | 0/1 | 0/0 | ./. | 0/0 | 1/1 |
| TRANME 13 | 1/1 | 0/0 | 0/1 | 0/0 | 0/1 | 1/1 | 0/0 |
| TRANME 14 | ./. | 0/0 | ./. | 0/0 | ./. | 1/1 | 0/0 |
| TRANME 15 | 0/1 | 0/0 | ./. | 0/0 | ./. | 1/1 | 0/0 |
| TRANME 16 | 0/1 | 0/0 | 0/1 | 0/0 | 0/0 | 1/1 | 0/0 |
| TRANME 17 | 0/0 | 0/0 | 0/0 | 0/0 | 0/0 | 1/1 | 0/0 |
| TRANME 18 | 1/1 | 0/0 | 0/0 | 0/0 | 0/0 | 1/1 | 0/0 |
| TRANME 19 | 0/1 | 0/0 | 0/0 | 0/0 | 0/0 | 0/1 | 0/1 |
| TRANME 20 | 0/1 | 0/0 | 1/1 | 0/0 | 0/0 | 1/1 | 0/0 |
| TRANME 21 | 0/0 | 0/0 | 0/0 | 0/0 | 0/0 | 0/1 | 0/1 |
| TRANME 22 | 1/1 | 0/0 | 0/1 | 0/0 | 0/0 | 1/1 | 0/0 |
| TRANME 23 | 1/1 | 0/0 | 1/1 | 0/0 | 0/0 | 1/1 | 0/0 |
| TRANME 24 | 1/1 | ./. | 1/1 | 0/0 | 0/0 | 1/1 | 0/0 |
| TRANME 25 | 1/1 | 0/0 | 0/1 | 0/0 | 0/0 | 1/1 | 0/0 |
| TRANME 26 | 0/1 | 0/0 | 0/0 | 0/0 | 0/0 | 0/1 | 0/0 |
| TRANME 27 | 0/1 | 0/0 | ./. | 0/0 | ./. | 0/1 | ./. |
| TRANME 28 | 1/1 | 0/0 | 0/0 | 0/0 | 0/0 | 0/1 | 0/0 |
| TRANME 29 | 1/1 | 0/0 | 0/1 | 0/0 | 0/0 | 1/1 | 0/0 |
| TRANME 30 | 1/1 | 0/1 | 1/1 | 0/1 | 0/0 | 1/1 | 0/0 |
| TRANME 31 | 0/1 | 0/0 | 0/1 | 0/0 | 0/0 | 1/1 | 0/0 |
| TRANME 32 | 0/1 | 0/0 | 0/1 | 0/0 | ./. | 1/1 | 0/0 |
| TRANME 33 | 0/0 | 0/0 | ./. | 0/0 | ./. | 0/1 | ./. |
| TRANME 34 | 1/1 | 0/0 | 0/1 | ./. | ./. | 1/1 | 0/0 |
| TRANME 35 | 1/1 | 0/0 | 0/1 | 0/0 | 0/0 | 1/1 | 0/0 |
| TRANME 36 | ./. | 0/0 | ./. | ./. | ./. | 0/1 | 0/0 |
| TRANME 37 | 0/1 | 0/0 | 0/1 | 0/0 | 0/0 | 0/1 | 0/0 |
| TRANME 38 | 0/0 | 0/0 | 0/0 | 0/0 | 0/0 | 0/1 | ./. |
| TRANME 39 | 1/1 | 0/0 | 0/1 | 0/0 | 0/0 | 1/1 | 0/0 |
| TRANME 40 | 1/1 | 0/0 | 0/1 | 0/0 | 0/0 | 1/1 | 0/0 |
| TRANME 41 | 0/1 | 0/0 | 1/1 | 0/0 | 0/0 | 1/1 | 0/0 |
| TRANME 42 | 1/1 | 0/0 | 0/1 | 0/0 | 0/0 | ./. | 0/0 |
| TRANME 43 | 0/1 | 0/0 | 0/1 | 0/0 | 0/0 | 1/1 | 0/0 |
| TRANME 44 | 0/1 | 0/0 | 1/1 | 0/0 | 0/1 | 1/1 | 0/0 |
| TRANME 45 | 1/1 | 0/0 | 1/1 | 0/0 | 0/0 | 0/1 | 0/0 |
| ROTSI 1 | 1/1 | 0/0 | 0/1 | 0/0 | ./. | 0/1 | ./. |
| ROTSI 2 | 0/1 | 0/0 | 1/1 | 0/0 | 0/0 | 0/1 | 0/1 |
| ROTSI 3 | 0/1 | 0/0 | 1/1 | 0/0 | 0/0 | 0/1 | 0/1 |
| ROTSI 4 | ./. | 0/0 | 0/0 | 0/0 | ./. | 1/1 | 0/0 |
| ROTSI 5 | 0/1 | 0/0 | 0/0 | 0/0 | 0/0 | 1/1 | 0/0 |
| ROTSI 6 | ./. | 0/0 | ./. | ./. | ./. | 1/1 | ./. |
| ROTSI 7 | 0/0 | 0/0 | 0/1 | 0/0 | 0/0 | 0/1 | 0/0 |
| ROTSI 8 | 0/1 | 0/0 | ./. | 0/0 | ./. | 0/1 | 0/0 |
| ROTSI 9 | 0/1 | 0/0 | ./. | 0/0 | ./. | 0/1 | ./. |
| ROTSI 10 | 0/0 | 0/0 | 1/1 | 0/0 | 0/1 | 1/1 | 0/0 |
| ROTSI 11 | ./. | ./. | ./. | 0/0 | 0/0 | 1/1 | 0/0 |
| ROTSI 12 | 0/0 | ./. | ./. | ./. | ./. | 1/1 | 0/0 |
| ROTSI 13 | 0/1 | 0/0 | 1/1 | 0/0 | 1/1 | 1/1 | 0/0 |
| ROTSI 14 | ./. | 0/0 | 1/1 | 0/0 | ./. | 0/1 | 0/0 |
| ROTSI 15 | 0/0 | 0/0 | 1/1 | 0/0 | 1/1 | 1/1 | 0/0 |
| ROTSI 16 | 0/1 | 0/0 | ./. | ./. | ./. | 0/1 | 0/1 |
| ROTSI 17 | 0/0 | 0/0 | 0/1 | 0/0 | 0/0 | 0/1 | 0/1 |
| ROTSI 18 | 0/1 | 0/0 | 0/1 | 0/0 | 0/0 | 1/1 | 0/0 |
| ROTSI 19 | 0/1 | 0/0 | 1/1 | 0/0 | 1/1 | 1/1 | 0/0 |
| ROTSI 20 | ./. | ./. | ./. | ./. | ./. | 1/1 | ./. |
| TUR 1 | 1/1 | 0/0 | 0/1 | 0/0 | 0/0 | 1/1 | 0/0 |
| TUR 2 | 1/1 | 0/1 | 1/1 | 0/1 | 0/0 | 0/1 | 0/1 |
| TUR 3 | 0/1 | 0/0 | 0/1 | 0/0 | 0/0 | 1/1 | 0/0 |
| TUR 4 | 0/1 | 0/0 | 1/1 | 0/0 | 0/0 | 1/1 | 0/1 |
| TUR 5 | 0/1 | 0/0 | 1/1 | 0/0 | 0/0 | 1/1 | 0/0 |
| TUR 6 | 0/1 | 0/0 | 0/1 | 0/0 | 0/0 | 1/1 | 0/0 |
| TUR 7 | 0/0 | 0/0 | 0/1 | 0/0 | 0/0 | 0/1 | 0/1 |
| TUR 8 | 0/1 | ./. | 1/1 | 0/0 | 0/1 | 0/1 | 0/1 |
| TUR 9 | 0/1 | 0/0 | 1/1 | 0/0 | 0/1 | 1/1 | 0/0 |
| TUR 10 | 0/1 | 0/1 | 1/1 | 0/1 | 0/0 | 0/1 | 0/0 |
| TUR 11 | 0/1 | 0/0 | 0/1 | 0/0 | 0/0 | 1/1 | 0/0 |
| TUR 12 | 0/1 | 0/1 | 1/1 | 0/1 | 0/0 | 1/1 | 0/0 |
| TUR 13 | 0/1 | 0/0 | 0/1 | 0/0 | 0/0 | 1/1 | 0/0 |
| TUR 14 | 0/0 | 0/0 | 0/1 | 0/0 | 0/0 | 0/1 | 1/1 |
| TUR 15 | 0/0 | 0/1 | 1/1 | 0/1 | 0/0 | 1/1 | 0/0 |
| TUR 16 | 0/1 | 0/0 | 1/1 | 0/0 | 0/1 | 1/1 | 0/0 |
| TUR 17 | 0/0 | 0/1 | 1/1 | 0/1 | 0/0 | 1/1 | 0/0 |
| TUR 18 | 0/1 | 0/0 | 1/1 | 0/0 | 0/0 | 0/1 | 0/1 |
| TUR 19 | 0/0 | 0/0 | 1/1 | 0/0 | 0/0 | 1/1 | 0/0 |
| TUR 20 | 0/1 | 0/0 | 0/1 | 0/0 | 0/1 | 1/1 | 0/0 |
| TUR 21 | 0/1 | 0/0 | 1/1 | 0/0 | 0/1 | 0/0 | 0/0 |
| TUR 22 | 0/0 | 0/0 | 1/1 | 0/0 | 0/1 | 0/1 | 0/1 |
| TUR 23 | 0/1 | 0/0 | 0/1 | 0/0 | 0/0 | 0/0 | 0/1 |
| TUR 24 | 1/1 | 0/0 | 0/1 | 0/0 | 0/1 | 1/1 | 0/1 |
| TUR 25 | 1/1 | 0/1 | 1/1 | 0/1 | 0/0 | 1/1 | 0/0 |
| TUR 26 | 0/1 | 0/0 | 0/1 | 0/0 | 0/0 | 1/1 | 0/0 |
| TUR 27 | 0/1 | 0/0 | 0/1 | 0/0 | 0/0 | 0/0 | 0/0 |
| TUR 28 | 1/1 | 0/0 | 0/1 | 0/0 | 0/0 | 0/1 | 0/0 |
| TUR 29 | 0/1 | 0/0 | 1/1 | 0/0 | 0/1 | 1/1 | 0/0 |
| TUR 30 | 0/1 | 0/0 | 1/1 | 0/0 | 1/1 | 0/1 | 0/1 |
| TUR 31 | ./. | 0/0 | 1/1 | 0/0 | 1/1 | 0/1 | 0/1 |
| TUR 32 | 0/1 | 0/0 | 1/1 | 0/0 | 0/0 | 1/1 | 0/0 |
| TUR 33 | 0/1 | 0/0 | 1/1 | 0/0 | 0/0 | 1/1 | 0/0 |
| TUR 34 | 0/0 | 0/1 | 1/1 | 0/1 | 0/0 | 0/0 | 0/0 |
| TUR 35 | 0/1 | 0/0 | 1/1 | 0/0 | 0/0 | 0/1 | 0/0 |
| TUR 36 | 1/1 | 0/0 | 1/1 | 0/0 | 0/0 | 1/1 | 0/0 |
| TUR 37 | 0/1 | 0/0 | 1/1 | 0/0 | 0/0 | 0/0 | 0/1 |
| TUR 38 | 0/1 | 0/0 | 1/1 | 0/0 | 0/1 | 0/1 | 0/0 |
| TUR 39 | 0/1 | 0/0 | 0/0 | 0/0 | 0/0 | 1/1 | 0/0 |
| TUR 40 | 0/0 | 0/0 | 1/1 | 0/0 | 0/0 | 0/1 | 0/0 |
| TUR 41 | 0/0 | 0/0 | 1/1 | 0/0 | 0/1 | 0/1 | 0/0 |
| TUR 42 | 0/1 | 0/0 | 1/1 | 0/0 | 0/0 | 0/0 | 0/1 |
| TUR 43 | 0/0 | 0/0 | 0/1 | 0/0 | 0/0 | 1/1 | 0/0 |
| TUR 44 | 0/1 | 0/0 | 0/1 | 0/0 | 0/0 | 0/1 | 0/0 |
| TUR 45 | 0/0 | 0/0 | 1/1 | 0/0 | 0/1 | 0/1 | 0/0 |
| TUR 46 | 0/0 | 0/0 | 0/1 | 0/0 | 0/1 | 1/1 | 0/0 |
| TUR 47 | 1/1 | 0/0 | 1/1 | 0/0 | 0/0 | 1/1 | 0/0 |
| TUR 48 | 1/1 | 0/0 | 0/1 | 0/0 | 0/0 | 0/1 | 0/0 |
| TUR 49 | 0/0 | 0/0 | 1/1 | 0/0 | 0/1 | 0/1 | 0/1 |
| TUR 50 | 0/0 | 0/0 | 1/1 | 0/0 | 0/0 | 0/1 | 0/0 |
| TUR 51 | 0/0 | 0/0 | 1/1 | 0/0 | 0/1 | 0/1 | 0/1 |
| TUR 52 | 0/0 | 0/0 | 1/1 | 0/0 | 0/1 | 0/1 | 0/0 |
| TUR 53 | 0/0 | 0/0 | 0/1 | 0/0 | 0/0 | 1/1 | 0/0 |
| TUR 54 | 0/0 | 0/0 | 1/1 | 0/0 | 0/0 | 0/0 | 0/1 |
| TUR 55 | 0/1 | 0/0 | 0/1 | 0/0 | 0/0 | 0/1 | 0/0 |
| TUR 56 | 0/1 | 0/0 | 1/1 | 0/0 | 0/0 | 0/1 | 0/0 |
| TUR 57 | 0/1 | 0/0 | 1/1 | 0/0 | 0/0 | 0/1 | 0/0 |
| TUR 58 | 0/1 | 0/0 | 0/1 | 0/0 | 0/0 | 0/0 | 0/0 |
| HUMER 1 | 0/1 | 0/0 | 0/1 | 0/0 | 0/0 | 1/1 | 0/0 |
| HUMER 2 | 1/1 | 0/0 | 0/1 | 0/0 | 0/0 | 1/1 | 0/0 |
| HUMER 3 | 1/1 | 0/0 | 1/1 | 0/0 | 0/1 | 1/1 | 0/0 |
| HUMER 4 | 1/1 | 0/0 | 1/1 | 0/0 | 0/0 | 0/1 | 0/1 |
| HUMER 5 | 0/1 | 0/0 | 1/1 | 0/0 | 0/0 | 1/1 | 0/0 |
| HUMER 6 | 0/1 | 0/0 | 0/1 | 0/0 | 0/0 | 1/1 | 0/0 |
| HUMER 7 | 0/1 | 0/0 | 0/1 | 0/0 | 0/0 | 1/1 | 0/0 |
| HUMER 8 | 0/0 | 0/0 | 1/1 | 0/0 | 0/0 | 1/1 | 0/0 |
| HUMER 9 | 0/1 | 0/0 | 0/0 | 0/0 | 0/0 | 1/1 | 0/0 |
| HUMER 10 | 1/1 | 0/0 | 1/1 | 0/0 | 0/0 | 1/1 | 0/0 |
| HUMER 11 | 0/1 | 0/0 | 1/1 | 0/0 | 0/0 | 1/1 | 0/0 |
| HUMER 12 | 1/1 | 0/0 | 0/1 | 0/0 | 0/0 | 0/1 | 0/1 |
| HUMER 13 | 1/1 | 0/0 | 1/1 | 0/0 | 1/1 | 1/1 | 0/0 |
| HUMER 14 | 0/1 | 0/0 | ./. | 0/0 | 0/0 | 1/1 | 0/0 |
| HUMER 15 | 1/1 | 0/0 | 0/0 | 0/0 | 0/0 | 1/1 | 0/0 |
| HUMER 16 | 0/1 | 0/0 | 0/1 | 0/0 | 0/1 | 1/1 | 0/0 |
| HUMER 17 | 0/0 | 0/0 | 1/1 | 0/0 | 0/0 | 0/0 | 1/1 |
| HUMER 18 | 0/1 | 0/0 | 0/0 | 0/0 | 0/0 | 1/1 | 0/0 |
| HUMER 19 | 0/1 | 0/0 | 0/1 | 0/0 | 0/0 | 0/1 | 0/1 |
| HUMER 20 | 1/1 | 0/0 | 0/1 | 0/0 | 0/1 | 1/1 | 0/0 |
| HUMER 21 | 0/1 | 0/0 | 1/1 | 0/0 | 0/0 | 1/1 | 0/0 |
| HUMER 22 | 0/1 | 0/0 | 0/1 | 0/0 | 0/0 | 1/1 | 0/0 |
| HUMER 23 | 1/1 | 0/0 | 0/1 | 0/0 | 0/0 | 0/1 | 0/1 |
| HUMER 24 | 0/1 | 0/0 | 0/1 | 0/0 | 0/0 | 1/1 | 0/0 |
| HUMER 25 | 1/1 | 0/0 | 0/0 | 0/0 | 0/0 | 1/1 | 0/0 |
| HUMER 26 | 1/1 | 0/0 | 1/1 | 0/0 | 1/1 | 0/1 | 0/1 |
| HUMER 27 | 1/1 | 0/0 | 0/1 | 0/0 | 0/1 | 0/1 | 0/1 |
| HUMER 28 | 0/1 | 0/0 | 0/0 | 0/0 | 0/0 | 1/1 | 0/0 |
| HUMER 29 | 1/1 | 0/0 | 0/1 | 0/0 | 0/0 | 1/1 | 0/0 |
| HUMER 30 | 0/1 | 0/0 | 0/1 | 0/0 | 0/0 | 1/1 | 0/0 |
| HUMER 31 | 1/1 | 0/0 | 0/1 | 0/0 | 0/1 | 1/1 | 0/0 |
| HUMER 32 | 1/1 | 0/0 | 1/1 | 0/0 | 0/1 | 1/1 | 0/0 |
| HUMER 33 | 0/1 | 0/0 | 0/1 | 0/0 | 0/0 | 1/1 | 0/0 |
| HUMER 34 | 1/1 | 0/0 | 0/1 | 0/0 | 0/0 | 0/1 | 0/0 |
| HUMER 35 | 0/0 | 0/0 | 0/1 | 0/0 | 0/0 | 0/1 | 0/1 |
| AWAS 1 | 0/0 | 0/1 | 1/1 | 0/1 | 0/1 | 1/1 | 0/0 |
| AWAS 2 | 0/1 | 0/0 | 1/1 | 0/0 | 0/0 | 0/1 | 0/0 |
| AWAS 3 | 0/1 | 0/0 | 1/1 | 0/0 | 0/1 | 1/1 | 0/0 |
| AWAS 4 | 0/1 | 0/0 | 0/1 | 0/0 | 0/0 | 1/1 | 0/0 |
| AWAS 5 | 0/0 | 0/0 | 0/1 | 0/0 | 0/0 | 1/1 | 0/0 |
| AWAS 6 | 0/0 | 1/1 | 1/1 | 1/1 | 0/0 | 1/1 | 0/0 |
| AWAS 7 | 0/0 | 0/1 | ./. | 0/1 | 0/0 | 1/1 | 0/0 |
| AWAS 8 | 0/0 | 0/0 | 1/1 | 0/0 | 0/0 | 0/1 | 0/1 |
| AWAS 9 | 0/1 | 0/1 | 1/1 | 0/1 | 0/0 | 1/1 | 0/0 |
| AWAS 10 | 0/0 | 0/0 | 1/1 | 0/0 | 0/0 | 1/1 | 0/0 |
| AWAS 11 | 0/0 | 0/0 | 1/1 | 0/0 | 0/0 | 1/1 | 0/0 |
| AWAS 12 | 0/0 | 0/1 | 1/1 | 0/1 | 0/0 | 0/1 | 0/1 |
| AWAS 13 | 0/1 | 0/0 | 0/1 | 0/0 | 0/0 | 1/1 | 0/0 |
| AWAS 14 | 0/0 | 0/0 | 1/1 | 0/0 | 0/1 | 1/1 | 0/0 |
| AWAS 15 | 1/1 | 0/0 | 1/1 | 0/0 | 0/0 | 0/1 | 0/1 |
| AWAS 16 | 0/1 | 0/0 | 1/1 | 0/0 | 0/0 | 1/1 | 0/0 |
| AWAS 17 | 1/1 | 0/0 | 1/1 | 0/0 | 0/0 | 0/1 | 0/1 |
| AWAS 18 | 0/1 | 1/1 | 1/1 | 1/1 | 0/0 | 1/1 | 0/0 |
| AWAS 19 | 0/1 | 0/0 | 1/1 | 0/0 | 0/0 | 0/0 | 1/1 |
| AWAS 20 | 0/1 | 0/0 | 0/1 | 0/0 | 0/0 | 1/1 | 0/0 |
| AWAS 21 | 0/1 | 0/0 | 0/1 | 0/0 | 0/0 | 1/1 | 0/0 |
| AWAS 22 | 0/0 | 0/0 | 0/1 | 0/0 | 0/0 | 1/1 | 0/0 |
| AWAS 23 | 0/1 | 0/0 | 1/1 | 0/0 | 0/0 | 0/1 | 0/1 |
| AWAS 24 | 0/0 | 0/0 | 1/1 | 0/0 | 0/0 | 1/1 | 0/0 |
| AWAS 25 | 1/1 | 0/1 | 1/1 | 0/1 | 0/1 | 1/1 | 0/0 |
| AWAS 26 | 0/1 | 0/0 | 1/1 | 0/0 | 0/0 | 1/1 | 0/0 |
| AWAS 27 | 0/0 | 0/0 | 1/1 | 0/0 | 0/0 | 1/1 | 0/0 |
| AWAS 28 | 0/1 | 0/0 | 1/1 | 0/0 | 0/0 | 1/1 | 0/0 |
| AWAS 29 | 0/0 | 0/1 | 1/1 | 0/1 | 0/0 | 0/1 | 0/0 |
| AWAS 30 | 0/1 | 1/1 | 1/1 | 1/1 | 0/0 | 0/1 | 0/1 |
| AWAS 31 | 0/0 | 0/0 | 1/1 | 0/0 | 0/0 | 0/1 | 0/1 |
| AWAS 32 | 0/1 | 0/0 | 1/1 | 0/0 | 0/0 | 0/1 | 0/1 |
| AWAS 33 | 0/0 | 0/1 | 0/1 | 0/1 | 0/0 | 0/1 | 0/1 |
| AWAS 34 | 0/1 | 0/0 | 1/1 | 0/0 | 0/1 | 0/1 | 0/1 |
| AWAS 35 | 1/1 | 0/0 | 0/1 | 0/0 | 0/0 | 1/1 | 0/0 |
| AWAS 36 | 0/1 | 0/0 | 1/1 | 0/0 | 0/0 | 0/1 | ./. |
| AWAS 37 | 0/0 | 0/0 | 0/1 | 0/0 | 0/0 | 1/1 | 0/0 |
| AWAS 38 | 0/0 | 0/1 | 1/1 | 0/1 | 0/0 | 1/1 | 0/0 |
| AWAS 39 | 0/1 | 0/0 | 1/1 | 0/0 | 0/0 | 1/1 | 0/0 |
| AWAS 40 | 0/1 | 0/0 | 1/1 | 0/0 | 0/0 | 1/1 | 0/0 |


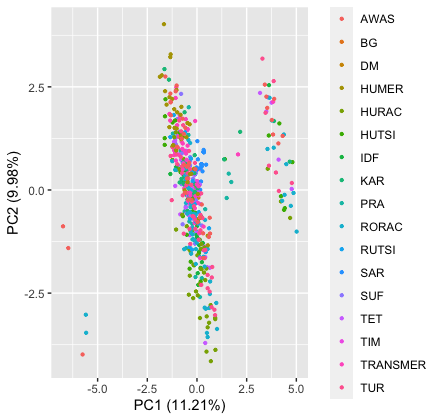


Figure S1. Score biplot of principal component analysis of the 17 SNPs for 17 sheep breeds. Individuals of different breeds are differently colored; (AWA: Hungarian Awassi, BG: Béni Guil, BTET: Babolna Tetra, DM: D’Man, HUME: Hungarian Merino, HUTS: Hungarian Tsigai, IDF: I de France, KAR: Botosani Karakul, PRA: Pramenka, TRANSMER: Transylvanian Merino, RORA: Romanian Racka, ROTSI: Romanian Tsigai, SAR: Sardi, SUF: Suffolk, TIM: Timahdite and TUR: Turcana).


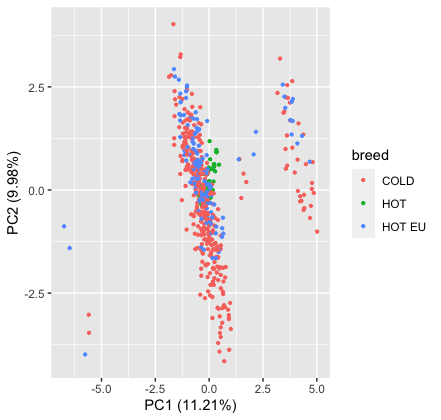


Figure S2. Score biplot of principal component analysis of the 17 SNPs for 17 sheep breeds. Breeds grouped by the characteristic; COLD: cold-tolerant breeds (Suffolk, Babolna Tetra, Ile de France, Hungarian Tsigai, Hungaria Racka, Hungarian Merino, Pramenka, Romanian Racka, and Turcana), HOT: heat tolerant breeds originated from Morocco (Béni Guil, D’Man, Timahdite and Sardi), HOT EU: Heat tolerant breeds reared in Europe (Hungarian Awassi, Botosani Karakul, Transylvanian Merino, and Romanian Tsigai).


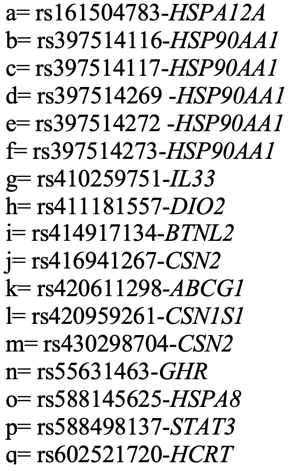

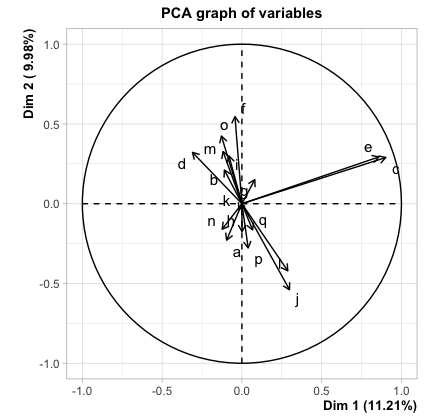


Figure S3. Loadings biplot of principal component analysis of the 17 SNPs for 17 sheep breeds.
